# SIRT7 links H3K36ac epigenetic regulation with genome maintenance in the mouse testis

**DOI:** 10.1038/s41467-026-72540-3

**Published:** 2026-04-28

**Authors:** Anna Guitart-Solanes, Mayra Romero, Andres Gamez-Garcia, Irene Fernández-Duran, Bryan A. Niedenberger, Cristina Madrid-Sandín, Norah Spears, Ignasi Roig, Christopher B. Geyer, Alejandro Vaquero, Karen Schindler, Berta N. Vazquez

**Affiliations:** 1 Chromatin Biology Laboratory, Josep Carreras Leukaemia Research Institute (IJC), 08916 Badalona, Spain; 2 Department of Genetics, Rutgers University, Piscataway, NJ 08854 USA; 3 Department of Anatomy and Cell Biology, Brody School of Medicine, Greenville, NC 27834 USA; 4 Cytology and Histology Unit, Department of Cell Biology, Physiology and Immunology, Universitat Autònoma de Barcelona (UAB), 08193 Cerdanyola del Vallès, Spain; 5 Genome Integrity and Instability Group, Institut de Biotecnologia i Biomedicina, Universitat Autònoma de Barcelona, 08913 Cerdanyola del Vallès, Spain; 6 Institute of Neuroscience and Cardiovascular Research, University of Edinburgh, Edinburgh, UK; 7 East Carolina Diabetes and Obesity Institute at East Carolina University, Greenville, NC 27834 USA

**Keywords:** Epigenetics, Ageing, Cell biology

## Abstract

Reproductive aging is an increasing health concern that affects family planning and overall well-being. While extensively studied in females, the mechanisms driving male reproductive aging remain largely unexamined. Here, we found that mammalian Sirtuin 7 sustains spermatogenesis in an age-dependent manner. Sirtuin 7 deficiency in mice increases histone H3 lysine 36 acetylation in spermatogonia and spermatocytes, a pattern also observed during natural aging, and leads to altered chromatin accessibility and increased vulnerability to genotoxic stress. Importantly, undifferentiated spermatogonia, required for continuous sperm production, become prematurely lost in Sirtuin 7 deficient mice and show increased genome damage accumulation during aging or environmental stress. These changes are concurrent with age-dependent defects in double-strand break repair and a meiotic delay. Taken together, our results indicate that Sirtuin 7 connects histone H3 lysine 36 acetylation epigenetic regulation to long-term genome stability in male germ cells, ensuring steady-state spermatogenesis during the lengthy male reproductive lifespan.

## Abstract (web summary)

Male reproductive aging is poorly understood. Here, the authors show that Sirtuin 7 preserves spermatogenesis by regulating H3K36 acetylation, chromatin accessibility, and DNA repair, protecting germline genome stability during aging and stress.

## Introduction

Reproductive potential declines with age in both female and male mammals. The repercussions of advanced maternal age on mammalian oocyte quality have been extensively studied^1^. However, the impact of aging on male germ cell development has not received comparable attention, as its impact on fertility rates is not as pronounced as it is in women^2–4^. Recent studies have revealed that aging substantially influences male fecundity, impairing testis function and reducing sperm quantity and quality^5–7^. Despite these important findings, the molecular mechanisms underlying age-related declines in male fertility remain poorly understood.

Spermatogenesis, the developmental program that continuously produces millions of sperm daily throughout the male reproductive lifespan, relies on functional spermatogonial stem cells (SSCs). SSCs closely regulate a balance between self-renewal, to maintain the stem cell pool, and the production of transit-amplifying undifferentiated spermatogonia that subsequently commit to differentiation before entering meiosis^8^. During meiosis, spermatocytes complete a series of unique events, including DNA double-strand break (DSB) formation, recruitment of DNA damage response proteins, homology searching, chromosome pairing, and the resolution of the DSBs as crossovers^9^. Epigenetic changes are critical for many aspects of meiotic progression, including gene expression regulation and chromatin organization^10,11^. Defects in epigenetic events can result in meiotic arrest, leading to spermatogenic failure and male infertility^12,13^.

Sirtuins are an evolutionarily conserved family of NAD^+^-dependent protein deacylases and ADP-ribosyltransferases with major roles in aging and age-related diseases, including cancer^14^, neurodegeneration^15^, and immunological disorders^16^. The mammalian genome encodes seven sirtuins (SIRT1–7), which have different cellular localizations and functions. SIRT1, SIRT6 and SIRT7 reside primarily in the nucleus, where they promote genome homeostasis through epigenetic regulation^17^. SIRT2 resides predominantly in the cytoplasm but also binds to chromosomes in mitotic cells to ensure correct chromosome segregation^14^. SIRT3, SIRT4, and SIRT5 localize to mitochondria, where they regulate cellular metabolism and antioxidant defenses.

Sirtuins play major roles in mammalian reproduction by regulating a myriad of processes, including germ cell development, meiotic recombination, chromosome segregation, and redox homeostasis^18,19^. Growing evidence links sirtuin function to reproductive aging, but the molecular mechanisms responsible for this association are poorly understood^19–21^. Female mice lacking *Sirt7* have reduced ovarian reserves and age-dependent subfertility characterized by compromised homologous chromosome synapsis, resulting in oocyte loss by birth and thereby a smaller primordial follicle pool^22^. Despite the importance of SIRT7 in normal early prophase I progression and reproductive longevity in females, its role in male gametogenesis has not been reported.

Here, we have examined the reproductive consequences of *Sirt7* deletion in male mice and discovered an age-dependent fertility decline associated with premature testis degeneration, altered germ cell development, and compromised sperm DNA quality. In the absence of SIRT7, male germ cells have a constitutive increase in histone H3 lysine 36 acetylation (H3K36ac), an epigenetic mark proposed to be major target of SIRT7 activity^23^. This increase affects mainly spermatogonia and spermatocytes, which displayed the biggest H3K36ac fold-change compared with WT controls. Remarkably, natural reproductive aging is also associated with an increase in H3K36ac levels. This correlation, particularly when considered in the context of our *Sirt7*^-/-^ phenotype, points to a causal connection between this epigenetic modification and germ cell aging. Interestingly, SIRT7-deficient spermatogonia display disturbed chromatin accessibility, which is directly linked to H3K36ac activity in a cellular model. Lack of SIRT7 causes increased genome damage accumulation in vitro and in vivo, coinciding with early loss of undifferentiated spermatogonia, and meiotic progression defects with aging. These results suggest an important role for SIRT7 in spermatogonia biology and early germ cell development. Taken together, our findings have major implications for male reproductive longevity and reveal a pivotal role for SIRT7 in safeguarding genome integrity by ensuring the correct establishment of epigenetic marks.

## Results

### Loss of SIRT7 causes premature male reproductive defects

**Fig. 1:**
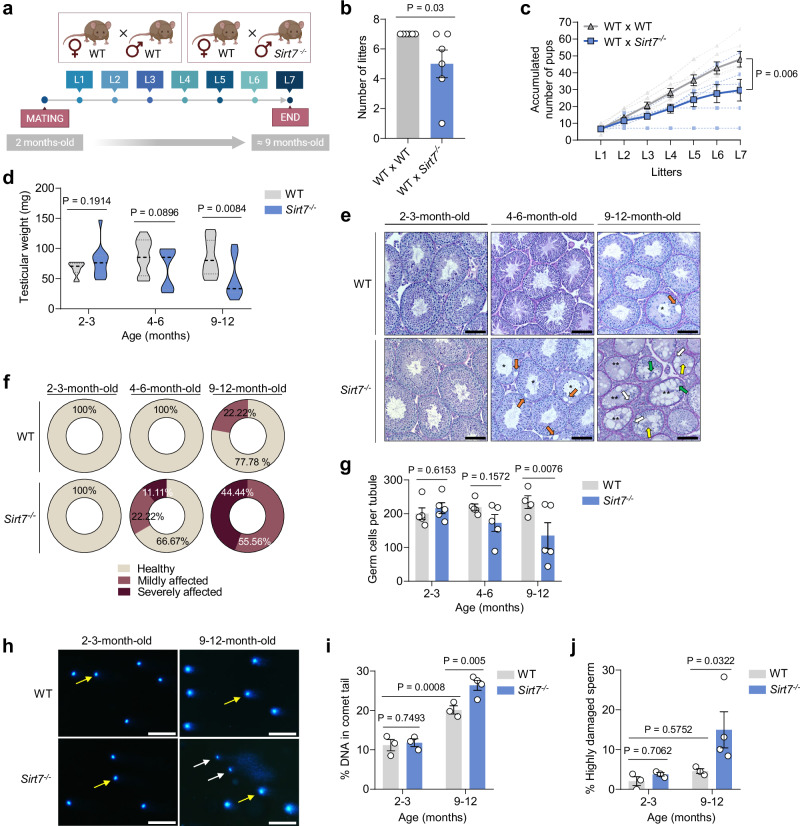
*Sirt7*^*-/-*^ male mice display premature reproductive decline. **a** Diagram of fertility trials. Single 2-month-old 129S1/Sv WT females were paired with single 129S1/Sv WT or *Sirt7*^*-/-*^ males; litter number and size were recorded until seven litters were produced by WT ×  WT crosses. Created in BioRender. Vazquez Prat, B. (2026) https://BioRender.com/pkt8lzk. **b** Number of litters produced by WT × WT and WT × *Sirt7*^*-/-*^ crosses. *n* = 6 matings/genotype. One-tailed unpaired Mann–Whitney test. **c** Cumulative pup production across seven litters (L1–L7) by WT × WT and WT × *Sirt7*^*-/-*^ crosses. Dotted lines represent individual matings (*n* = 6 matings/genotype); highlighted symbols and lines indicate mean ± SEM. Two-way repeated-measures ANOVA. **d** Testis weights of WT and *Sirt7*^*-/-*^ mice. 2-3-month-old: WT, *Sirt7*^*-/-*^ (*n* = 12); 4–6-month-old: WT (*n* = 15), *Sirt7*^*-/-*^ (*n* = 14); 9–12-month-old: WT (*n* = 13), *Sirt7*^*-/-*^ (*n* = 14). Two-tailed t-tests. **e** PAS-hematoxylin-stained testis sections. *n* = 9 independent testes/group. Asterisks indicate seminiferous tubules with isolated vacuolation (*) or complete spermatogenic disruption (**). Arrows indicate vacuolation initiation (orange), remaining spermatogonia (white), spermatocytes (green), and spermatids (yellow) in atrophic tubules. Scale bar, 100 µm. **f** Pie charts showing testicular phenotypes based on histological vacuolation and germ cell content. *n* = 9 independent testes/group. **g** Germ cell number per seminiferous tubule in each age group. *n* = 5 independent testes/group, except 9–12-month-old WT (*n* = 4). Two-way ANOVA with uncorrected Fisher’s LSD test. **h** Representative sperm comet assay images of samples quantified in (**i**, **j**). Yellow arrows indicate comets suitable for tail-DNA quantification. White arrows indicate highly fragmented “hedgehog” sperm scored separately. Scale bar, 100 µm. **i**, **j** Percentage of DNA in the comet tail (**i**) and hedgehog comets (**j**) in 2–3- and 9–12-month-old WT and *Sirt7*^*-/-*^ after comet assay. *n* = 3 biologically independent epididymal samples/group, except 9–12-month-old *Sirt7*^*-/-*^ group (*n* = 4). Each dot shows the mean of more than 200 analyzed comets per sample. Two-way ANOVA with uncorrected Fisher’s LSD test. In this figure, bar plots and error bars show mean values ± SEM. Source data are provided as a Source data file.

We previously reported an important role for SIRT7 in meiotic recombination in female mice, that is essential for maintaining a normal reproductive lifespan^22^. To assess SIRT7’s role in spermatogenesis and during aging, we analyzed reproductive characteristics in previously validated *Sirt7* knockout (*Sirt7*^*-/-*^) male mice^24^. First, fertility studies were conducted by crossing wild-type (WT) females with either WT or *Sirt7*^*-/-*^ males, then counting the number of litters and cumulative pup production (Fig. 1a–c). On average, despite variation in litter production among individuals, *Sirt7*^-/-^ males produced significantly fewer litters than did WT controls (Fig. 1b). Although there was no apparent difference in the size of the first two litters, *Sirt7*^*-/-*^ males produced fewer pups in subsequent litters, resulting in fewer pups being produced overall (Fig. 1c). In total, *Sirt7*^*-/-*^ males sired nearly half as many pups as did similarly aged WT control males. Taken together, our results reveal a significant age-dependent fertility decline in *Sirt7*^*-/-*^ males.

Physiological testis aging is accompanied by histopathological changes, including impaired spermatogenesis and vacuolation due to germ cell losses within the seminiferous epithelium^6,25^. Notably, in 9–12-month-old *Sirt7*^*-/-*^ male mice testis weights were significantly reduced (Fig. 1d). This phenotype, although not statistically significant, was already present in a proportion of 4–6-month-old male *Sirt7*^*-/-*^ testes, suggestive of seminiferous epithelium degeneration with age in *Sirt7*^*-/-*^ mice (Fig. 1d). To visualize histopathological changes, periodic acid-Schiff (PAS)-hematoxylin staining was performed on full testis sections from three age groups of WT and *Sirt7*^*-/-*^ mice (Fig. 1e and Supplementary Fig. 1a). Mice aged 2–3 months of both genotypes had normal-appearing spermatogenesis (Fig. 1e, f). However, aging resulted in the appearance of histological abnormalities in both WT and *Sirt7*^*-/-*^ testes, which were classified into three categories: healthy, if at least 80% of seminiferous tubules displayed normal spermatogenesis; mildly affected, if isolated vacuolation appeared in more than 20% of seminiferous tubules but spermatogenesis remained continuous; and severely affected, if more than 20% of tubules presented severe vacuolation with spermatogenesis disruption (Supplementary Fig. 1a). At 4–6 months of age, 22% of *Sirt7*^*-/-*^ testes were mildly affected, a phenotype absent in WT mice of the same age. Notably, these mild histological alterations were also observed in 22% of WT testes aged 9–12 months. At 4–6 months of age, 11% of *Sirt7*^*-/-*^ testes also presented a severely affected histological phenotype (Fig. 1e, f, and Supplementary Fig. 1a). These results revealed precocious degeneration and germ cell loss within seminiferous epithelia in *Sirt7*^*-/-*^ mice. Additionally, the age-related defects observed in WT testes paralleled the natural fertility decline occurring in WT males with aging, when comparing fecundity of 2- and 12-month-old animals (Supplementary Fig. 1b). Severe vacuolation affected ~45% of the testes of 9–12-month-old *Sirt7*^*-/-*^ individuals. This phenotype was also evident, albeit with a milder manifestation, in the remaining ~55% of the aged *Sirt7*^*-/-*^ testes (Fig. 1e, f, and Supplementary Fig. 1a). Consistently, the number of germ cells per seminiferous tubule was significantly reduced in testes of 9–12-month-old *Sirt7*^*-/-*^ mice (Fig. 1g). The abundance of Sertoli cells, which are key regulators of spermatogenesis^26^ and identified by SOX9 immunofluorescence, did not differ between WT and *Sirt7*^*-/-*^ testes in 9–12-month-old animals (Supplementary Fig. 1c, d). Collectively, these findings indicate that premature germ cell loss in *Sirt7*^*-/-*^ testes underlies the age-related decline in the production of offspring by *Sirt7*^*-/-*^ mice. Additionally, the variability in testis degeneration may explain the variation in the numbers of litters produced by *Sirt7*^*-/-*^ males.

Given that levels of DNA damage increase with age in human and mouse sperm^25,27^, we compared DSB levels in cauda epididymal sperm from WT and *Sirt7*^*-/-*^ mice of the 2–3- and 9–12-month-old groups using the comet assay (Fig. 1h–j). Consistent with previous studies, our results revealed significantly increased sperm DNA fragmentation associated with paternal aging, irrespective of genotype (Fig. 1i). Compared with WT controls, *Sirt7*^*-/-*^ sperm from 9 to 12-month-old mice tended to have a significantly higher percentage of DNA in comet tails (Fig. 1i). Importantly, ~15% of sperm from 9 to 12-month-old *Sirt7*^*-/-*^ mice presented a characteristic “hedgehog” comet morphology, indicative of a severely damaged DNA (Fig. 1h, j). Collectively, these results indicate that sperm from older *Sirt7*^*-/-*^ mice have increased DNA damage, thereby highlighting a critical role for SIRT7 in maintaining genomic integrity to ensure production of high-quality sperm during the aging process.

### SIRT7 regulates global H3K36ac levels in testis

**Fig. 2:**
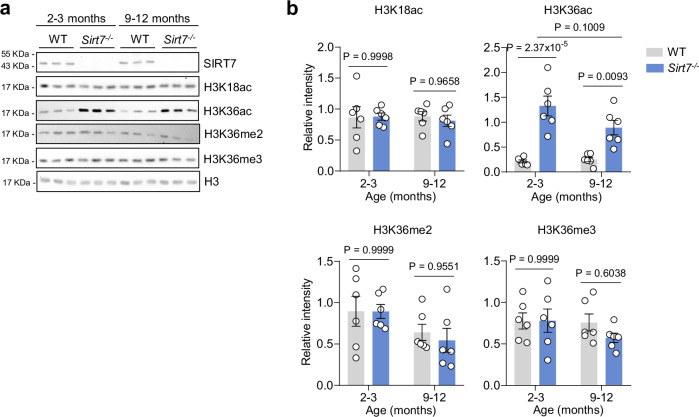
SIRT7 regulates H3K36ac in mouse testes. **a** Representative Western blot images of modified histones in whole-testis protein lysates from 2-3- and 9-12-month-old 129S1/Sv WT and *Sirt7*^*-/-*^ mice. Each lane represents an independent testis sample. Six biological replicates analyzed in two technical replicates, with similar results. **b** Densitometry-based quantifications of relative epigenetic mark levels in Western blots (**a**), normalized to H3. *n* = 6 biological replicates, each represented by one dot. Bar plots and error bars show mean ± SEM. Two-way ANOVA with Tukey’s multiple comparisons tests. Source data are provided as a Source data file.

Given that SIRT7 is a bona fide epigenetic enzyme^23,28^, we first conducted a broad epigenetic mark screen in testes from 2-3- and 9-12-month-old WT and *Sirt7*^*-/-*^ mice (Fig. 2 and Supplementary Fig. 2). This analysis allowed us to compare the chromatin landscape of young testes with that of older animals, where the phenotype was fully manifested in *Sirt7*^*-/-*^ testes. First, we conducted Western blots of whole testis lysates to analyze direct targets of SIRT7 deacetylase activity, including H3K18ac^28^ and H3K36ac^23,29^. Global levels of H3K18ac were similar between WT and *Sirt7*^*-/-*^ testes (Fig. 2a, b), consistent with previous reports suggesting a role for SIRT7 in regulating this epigenetic mark at specific loci and excluding global H3K18ac regulation^24,28^. In contrast, a dramatic increase in H3K36ac was detected in *Sirt7*^*-/-*^ testes when compared to levels in WT controls, irrespective of age.

Higher H3K36ac levels could preclude or limit deposition of H3K36 methylation^30^. However, despite substantial changes in H3K36ac levels in *Sirt7*^*-/-*^ testes, global H3K36me2/me3 levels did not differ between genotypes (Fig. 2a, b). This is consistent with a previous report suggesting no overlap between the genomic distributions of H3K36ac and H3K36me2/3^31^. Further assessment of global levels of epigenetic marks linked to spermatogenesis progression, such as H3K4 methylation^32^, or to sirtuin activity, such as H4K20 or H3K9 methylations^14,33^, revealed no significant differences in *Sirt7*^*-/-*^ testes (Supplementary Fig. 2a, b). Together, these results imply that SIRT7 is required to specifically deacetylate H3K36ac in the mouse testis.

### SIRT7 regulates H3K36ac in spermatogonia and spermatocytes

**Fig. 3:**
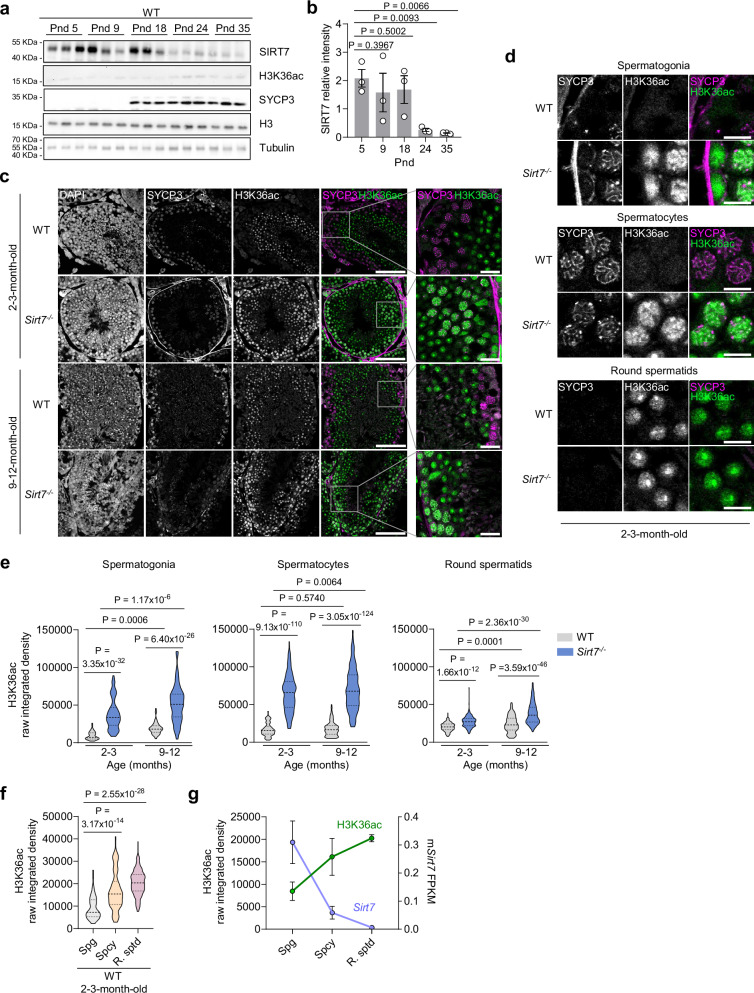
SIRT7 regulates H3K36ac in spermatogonia and spermatocytes. **a** Western blots of SIRT7 and H3K36ac in testes from 129S1/Sv WT mice at the indicated post-natal days (Pnd). SYCP3 was used as a spermatocyte marker, and H3 and tubulin as loading controls. Three biological replicates, analysed in technical duplicates with similar results. **b** Densitometry-based quantification of SIRT7 levels (**a**), normalized to tubulin (*n* = 3 independent testes/group). Bar plots and error bars indicate mean ± SEM. One-way ANOVA with uncorrected Fisher’s LSD test. **c** SYCP3 and H3K36ac immunostaining in testis sections of 2–3- and 9–12-month-old 129S1/Sv WT and *Sirt7*^*-/-*^ mice. Three biological replicates. Scale bars, 100 µm; zoom images, 20 µm. **d** H3K36ac immunostaining in 2–3-month-old WT and *Sirt7*^*-/-*^ samples in spermatogonia, spermatocytes, and round spermatids. Spermatogonia were identified by basal position in the tubule, SYCP3 absence, and morphology; spermatocytes by SYCP3 positivity; and round spermatids by SYCP3 absence, adluminal position, small size, and distinct DAPI-positive chromocenter. Three biological replicates. Scale bar, 10 µm. **e** Quantification of H3K36ac fluorescence intensity in the three spermatogenic cell types in 2–3- and 9–12-month-old WT and *Sirt7*^*-/-*^ samples. **f** Quantification of H3K36ac fluorescence intensity in the three spermatogenic cell types in 2–3-month-old WT samples. **g** Plot of H3K36ac fluorescence intensity in 2–3-month-old WT germ cells and mouse *Sirt7* transcript levels from public sc-RNAseq datasets^35^. Data are presented as mean ± SEM. **e**–**g** Density plots show individual cell measurements from 3 biological replicates. Spermatogonia: 2–3-month-old (m.o.) WT (*n* = 54 cells), *Sirt7*^*-/-*^ (*n* = 80); 9–12-m.o. WT (*n* = 87), *Sirt7*^*-/-*^ (*n* = 53). Spermatocytes: 2–3-m.o. WT (*n* = 212), *Sirt7*^*-/-*^ (*n* = 231); 9–12-m.o. WT (*n* = 224), *Sirt7*^*-/-*^ (*n* = 222). Spermatids: 2–3-m.o. WT (*n* = 234), *Sirt7*^*-/-*^ (*n* = 221); 9–12-m.o. WT (*n* = 219), *Sirt7*^*-/-*^ (*n* = 236). One-way ANOVA with uncorrected Fisher’s LSD tests. In this figure, Spg spermatogonia, Spcy spermatocytes, Sptd spermatids, R. Sptd round spermatids. Source data are provided as a Source data file.

Western blot analyses showed a clear deregulation of H3K36ac levels in SIRT7-deficient testes (Fig. 2). Analyses of publicly available scRNA-seq data from human^34^ and mouse^35^ testes indicated that *Sirt7* transcripts were abundant in spermatogonia and spermatocytes but substantially decreased in post-meiotic spermatids (Supplementary Fig. 3a–d). Consistent with these transcriptomic profiles, analysis of SIRT7 protein dynamics during the first wave of spermatogenesis^36^ (Fig. 3a, b) demonstrated a high level of SIRT7 expression on postnatal days (Pnd) 5 and 9, stages dominated by spermatogonia, and on Pnd 18, when all stages of meiotic prophase I spermatocytes were already present in the testis (Fig. 3a, b). SIRT7 protein levels declined markedly by Pnd 24 and Pnd 35, corresponding to the presence of post-meiotic round and elongated spermatids, respectively (Fig. 3a, b, and Supplementary Fig. 3e). Together, these results suggest a previously unrecognized role for SIRT7 in early spermatogenic populations. Notably, H3K36ac levels displayed an inverse association with SIRT7, being low at Pnd 5 and Pnd 9 and gradually increasing as spermatogenesis progressed (Fig. 3a). To further understand the contribution of SIRT7 and H3K36ac in spermatogenesis during testicular aging, we analyzed H3K36ac distribution in testicular sections from 2-3- and 9-12-month-old WT and *Sirt7*^*-/-*^ mice (Fig. 3c). Independently of age, we found that the absence of SIRT7 led to a marked increase in H3K36ac across all germ cell populations, with spermatogonia and spermatocytes displaying the greatest changes (Fig. 3c–e). Notably, we observed an inverse correlation between *Sirt7* and H3K36ac levels in WT testes, with H3K36ac levels barely detectable in spermatogonia and spermatocytes and increasing in round spermatids (Fig. 3d, f), while *Sirt7* mRNA levels exhibited the opposite trend (Fig. 3g). Levels of H3K36ac remained high in elongating spermatids, but as expected, were depleted in condensing spermatids, where most histones are replaced by protamines^11^ (Supplementary Fig. 3f). The increase in H3K36ac and the decline in *Sirt7* levels from spermatogonia to spermatids indicate an important role of SIRT7-dependent H3K36ac deacetylation in this developmental process. Notably, *Sirt7* transcript levels were the highest among all sirtuins in human spermatogonia and spermatocytes, and were also among the highest in these populations in mice (Supplementary Fig. 3a–d), reinforcing the potential role of SIRT7 in early spermatogenesis.

Notably, H3K36ac levels were significantly higher in spermatogonia from 9-12-month-old WT testis samples than those from 23-month-old WT controls (Fig. 3c, e), unveiling that natural aging is associated with an increase in this epigenetic mark in this testicular population. Immunostaining of testis sections did not allow us to observe age-related H3K36ac changes in spermatocytes, so instead we analyzed H3K36ac dynamics during prophase I in meiotic chromatin spreads from 2-3- and 9-12-month-old WT and *Sirt7*^*-/-*^ mouse testes. WT spermatocytes exhibited low H3K36ac levels in leptonema and zygonema that increased in pachynema and to a greater extent in diplonema (Supplementary Fig. 3g, h). This result aligns with publicly available scRNA-seq datasets showing *Sirt7* transcript levels consistently declining during meiosis (Supplementary Fig. 3i). Importantly, H3K36ac levels were higher in 9–12-month-old WT pachynema spermatocytes than those from 2-3-month-old WT controls (Supplementary Fig. 3j, k), indicating that H3K36ac deregulation also accompanied natural spermatocyte aging.

### Increased H3K36ac does not correlate with gene expression

**Fig. 4:**
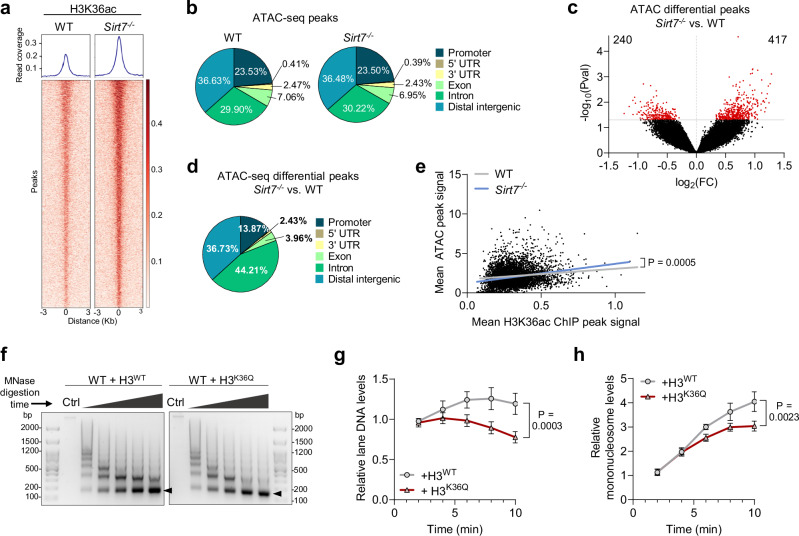
SIRT7-dependent H3K36ac deregulation is positively correlated with accessibility. **a** Heatmap of ChIP-seq data showing genomic occupancy of H3K36ac peaks in WT and *Sirt7*^*-/-*^ Pnd 5 spermatogonia. Representative heatmaps from 3 biological replicates. **b** Genomic distribution of WT and *Sirt7*^*-/-*^ consensus ATAC-seq peaks in Pnd 5 spermatogonia. Three biological replicates. **c** Volcano plot showing significantly upregulated and downregulated ATAC-seq peaks in *Sirt7*^*-/-*^ Pnd 5 spermatogonia relative to WT (in red, significant differential peaks defined using *P* < 0.05, calculated with DESeq2’s Wald test without corrections for multiple comparisons). **d** Genomic distribution of differential ATAC-seq peaks in *Sirt7*^*-/-*^ Pnd 5 spermatogonia relative to WT. **e** Dot plot showing the correlation between H3K36ac ChIP-seq peak signal and ATAC-seq peak signal measured within H3K36ac consensus peaks in Pnd 5 spermatogonia. Linear correlation is shown independently for each genotype. Linear regression analysis was used to assess the correlation between variables within each genotype (WT, *P* = 3.38 × 10e-6; *Sirt7*^*-/-*^, *P* = 1.66 × 10e-56). Slopes were compared within the linear regression analysis (*F*(1, 5413) = 12.04; *P* = 0.0005). **f** Agarose gel banding showing chromatin digestion of WT GC-2spd(ts) cells overexpressing H3^WT^ or H3^K36Q^ following MNase treatment for increasing times (0, 2, 4, 6, 8, and 10 min). Three biological replicates derived from the same cellular clone. Black arrowheads indicate bands corresponding to the mononucleosome fraction. **g** Densitometry-based quantification of chromatin digestion showing total amount of DNA at each time point (whole lane) normalized to the amount of DNA at *t* = 2 min. **h** Densitometry-based quantification of chromatin digestion showing mononucleosome fraction intensity normalized to the amount of DNA at *t* = 2 min. **g**, **h** *n* = 3 biological replicates derived from the same cellular clone. Data are presented as mean values ± SEM. Differences between WT and H3^K36Q^ curves were assessed by comparing quadratic nonlinear regression fits using extra-sum-of-squares F tests (**g**, *F*(3, 24) = 9.298; **h**, *F*(3, 24) = 6.450). Source data are provided as a Source data file.

SIRT7 can function as a transcriptional activator or repressor in a gene-context-dependent manner^37,38^, but it is unknown whether this involves regulation of H3K36ac. Since SIRT7 is highly expressed in spermatogonia (Fig. 3a, b, and Supplementary Fig. 3a–d), we used WT and *Sirt7*^*-/-*^ neonatal testes, which are primarily composed of this cell population^39^, and applied a previously described plating-based strategy to remove somatic cells and enrich samples in spermatogonia^40,41^. Using this approach, the majority of isolated cells were positive for PLZF, a well-established marker of undifferentiated spermatogonia^42^ (Supplementary Fig. 4a). WT and *Sirt7*^*-/-*^ spermatogonia-enriched samples were subsequently used to generate genome-wide H3K36ac maps and transcriptomic profiles using ChIP- and bulk RNA-sequencing. As expected, H3K36ac ChIP-seq intensity signal was markedly higher in *Sirt7*^*-/-*^ cells compared to WT controls (Fig. 4a). H3K36ac-enriched chromatin was located primarily at gene promoters in WT and *Sirt7*^*-/-*^ spermatogonia (Supplementary Fig. 4b), representing 98% and 92% of the total peaks, respectively (Supplementary Fig. 4b). These results are consistent with previous reports showing a preferential enrichment of H3K36ac at promoter regions^43^. In *Sirt7*^*-/-*^ cells, H3K36ac also localized to distal intergenic (4%) and introns (3%) (Supplementary Fig. 4b), indicative of SIRT7 deacetylase activity outside of canonical transcription start sites.

Despite the enrichment of H3K36ac at gene promoters (Supplementary Fig. 4b), transcriptomic analysis of differentially expressed genes identified 26 significantly upregulated and 22 significantly downregulated transcripts in *Sirt7*^*-/-*^ spermatogonia (Supplementary Fig. 4c), suggesting a modest role for SIRT7 in transcriptional regulation in this cell type. Notably, integrating H3K36ac ChIP-seq and RNA-seq datasets did not reveal a correlation between H3K36ac differential peaks and differential gene expression in *Sirt7*^*-/-*^ spermatogonia (Supplementary Fig. 4d, e), indicating that elevated H3K36ac is not correlated with gene expression changes in the absence of SIRT7.

### Lack of SIRT7 results in altered chromatin accessibility

Previous work showed that H3K36ac facilitates chromatin relaxation in yeast^30^, raising the possibility that SIRT7-dependent H3K36ac deacetylation in spermatogonia and spermatocytes regulates chromatin accessibility. To test this possibility, we conducted ATAC-seq in WT and *Sirt7*^*-/-*^ spermatogonia-enriched samples. Chromatin accessibility peaks were detected using peak calling on merged nucleosome-free fragment profiles. Consistent with previous reports^44^, most accessible regions were located at distal intergenic regions (37%), introns (30%), promoter regions (23%) and exons (7%) in both WT and *Sirt7*^*-/-*^ samples (Fig. 4b). Differential accessibility analysis identified 417 regions with increased chromatin accessibility and 249 regions with decreased accessibility (Fig. 4c) in *Sirt7*^-/-^ spermatogonia. Most of these regions were located within introns (44%), distal intergenic regions (37%), and gene promoters (14%) (Fig. 4d). Gene ontology analysis of the nearest associated genes revealed a broad range of biological functions and phenotypes (Supplementary Fig. 4f). Notably, regions with altered accessibility in distal intergenic and exonic regions were enriched for functions related to testis biology and male fertility. To test whether H3K36ac levels were associated with chromatin accessibility, we compared the mean signals of H3K36ac ChIP-seq and ATAC-seq peaks across genomic regions in WT and *Sirt7*^*-/-*^ cells (Fig. 4e). Both genotypes showed a significant positive correlation, indicating that regions enriched for H3K36ac tended to exhibit greater chromatin accessibility. Notably, linear regression analysis revealed a modest but significant difference in slope between the genotypes, suggesting that loss of SIRT7 enhances the coupling between H3K36ac and chromatin openness.

To test the direct involvement of the SIRT7-dependent H3K36ac regulation in chromatin accessibility, we switched to a readily manipulable cell line of germ cell origin. We introduced a CRISPR-Cas9-induced deletion of the *Sirt7* gene into the spermatocyte-derived immortalized mouse cell line GC-2spd(ts). This deletion led to high H3K36ac levels, as found in primary cells (Supplementary Fig. 4g). Micrococcal nuclease (MNase) assays were then conducted with WT and *Sirt7*^*-/-*^ cells, consisting of MNase treatment for increasing time periods, followed by DNA fragment analysis (Supplementary Fig. 4h). Notably, the total amount of DNA remaining after digestion was significantly lower in the absence of SIRT7 (Supplementary Fig. 4h, i). While chromatin treatment with MNase resulted in linker DNA digestion and mononucleosome accumulation in WT cells, this accumulation was markedly reduced in *Sirt7*^*-/-*^ cells (Supplementary Fig. 4h, j). Our results suggest that SIRT7 deficiency in GC-2spd(ts) cells disrupts chromatin structure, possibly affecting the nucleosome core organization.

To evaluate whether H3K36ac directly contributes to this phenotype, we generated a histone H3 mutant in which lysine 36 was replaced by glutamine (H3^K36Q^), a modification known to mimic constitutive acetylation^24^. GC-2spd(ts) cells were transduced with constructs expressing either WT H3 (H3^WT^) or the acetylation-mimic mutant (H3^K36Q^), followed by selection. Successful expression of WT and mutant histones was confirmed by Western blot analysis (Supplementary Fig. 4k). MNase assays revealed that expression of H3^K36Q^ resulted in a reduced quantity of DNA after digestion (Fig. 4f, g) and impaired mononucleosome accumulation (Fig. 4f, h), supporting a direct link between H3K36ac and altered chromatin accessibility in the absence of SIRT7.

### SIRT7 absence leads to increased DNA damage in spermatogonia

**Fig. 5:**
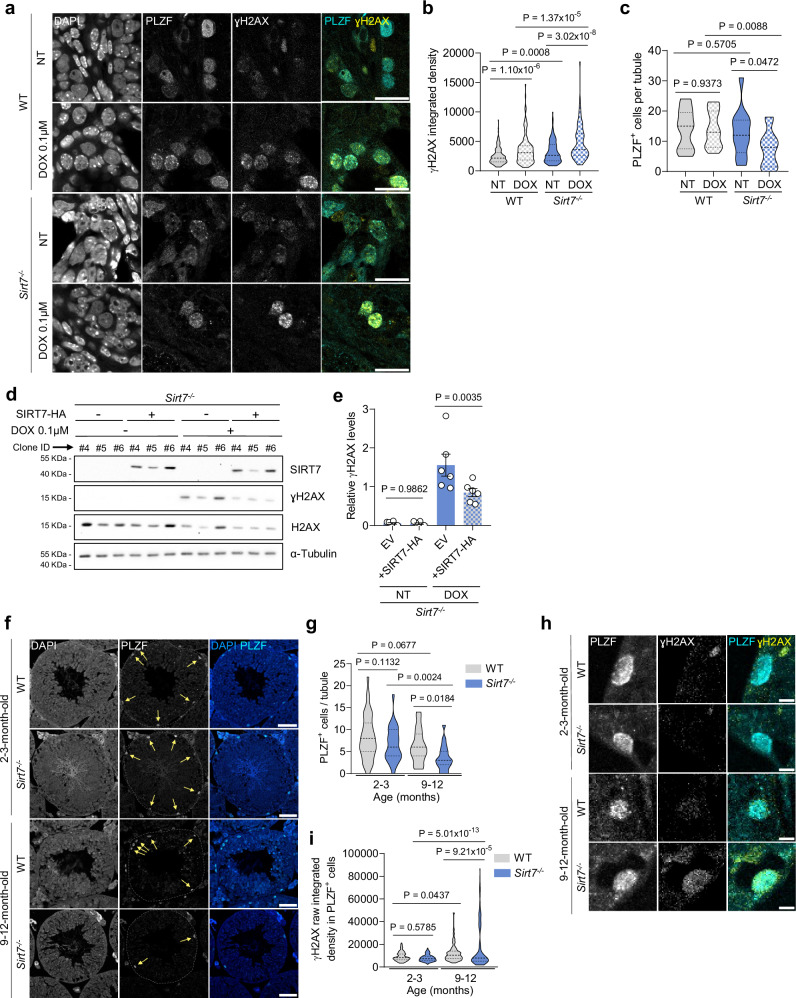
Environmental stress and aging exacerbate genome damage accumulation in *Sirt7*^*-/-*^ undifferentiated spermatogonia. **a** PLZF and γH2AX immunostaining in WT and *Sirt7*^*-/-*^ Pnd 5 cultured testicular tissue sections, non-treated (NT) or treated with 0.1 µM doxorubicin (DOX) for 16 h. Two biological replicates. Scale bar, 20 µm. **b** ɣH2AX fluorescence intensity quantification in PLZF^+^ spermatogonia (**a**). Two biological replicates, individual cell measurements shown. WT: NT (*n* = 207 cells), DOX (*n* = 161); *Sirt7*^*-/-*^: NT (*n* = 159), DOX (*n* = 93). Two‑way ANOVA with uncorrected Fisher’s LSD test. **c** Quantification of PLZF^+^ spermatogonia per seminiferous tubule in cultured WT and *Sirt7*^*-/-*^ testicular tissue. Two biological replicates, individual cell measurements shown. WT: NT, DOX (*n* = 17 tubules); *Sirt7*^*-/-*^: NT (*n* = 12), DOX (*n* = 13). Two-way ANOVA with uncorrected Fisher’s LSD test. **d** Western blot of SIRT7 and ɣH2AX in NT and 0.1 µM DOX-treated *Sirt7*^*-/-*^ GC-2spd(ts) cells after the reintroduction of empty vector (EV) or SIRT7-HA. Three independent clones. Two biological replicates. **e** Densitometry-based quantification of ɣH2AX relative to H2AX (**d**), *n* = 6 biological replicates. Bar plots indicate the mean value ± SEM. Two‑way ANOVA with uncorrected Fisher’s LSD test. **f** PLZF immunostaining in 2–3- and 9–12-month-old WT and *Sirt7*^*-/-*^ testis sections. Three biological replicates. Dotted lines delineate seminiferous tubules. Yellow arrows indicate PLZF^+^ spermatogonia. Scale bar, 50 µm. **g** Quantification of PLZF^+^ spermatogonia per seminiferous tubule. Three biological replicates, individual cell measurements shown. 2-3-month-old: WT (*n* = 37 tubules), *Sirt7*^*-/-*^ (*n* = 26); 9–12-month-old: WT (*n* = 19), *Sirt7*^*-/-*^ (*n* = 42). Two‑way ANOVA with uncorrected Fisher’s LSD test. **h** PLZF and ɣH2AX immunostaining in 2–3- and 9–12-month-old WT and *Sirt7*^*-/-*^ testis sections. Three biological replicates. The 9–12-month-old *Sirt7*^*-/-*^ image is representative of cells with high ɣH2AX levels in that group. Scale bar, 5 µm. **i** ɣH2AX fluorescence intensity quantification in PLZF^+^ spermatogonia. Three biological replicates, individual cell measurements shown. 2-3--month-old: WT (*n* = 212 cells), *Sirt7*^*-/-*^ (*n* = 180); 9–12-month-old: WT (*n* = 128), *Sirt7*^*-/-*^ (*n* = 162). Two-way ANOVA with Tukey’s multiple comparisons test. Source data are provided as a Source data file.

In yeast, H3K36ac couples chromatin accessibility to DNA damage repair^30^, suggesting that elevated H3K36ac in the absence of SIRT7 could compromise DNA repair pathways and promote genome instability. Knowing that SIRT7 levels are high in spermatogonia (Fig. 3a, b), we hypothesized that SIRT7 activity sustained genome integrity in this cell population. To test this, we used an acute genotoxic stress model in which testicular tissue from WT and *Sirt7*^*-/-*^ mice was cultured in the presence or absence of the genotoxic agent doxorubicin, a drug known to disturb the spermatogonial pool^39,45^. Analysis of γH2AX, a histone mark associated with DSB formation^46^, in PLZF^+^ spermatogonia, revealed a significant increase in γH2AX signal following doxorubicin treatment in WT samples (Fig. 5a, b). *Sirt7*^*-/-*^ testes exhibited elevated γH2AX levels even under basal conditions (Fig. 5a, b), suggesting impaired response to culture-induced stress. Upon doxorubicin exposure, γH2AX levels further increased in *Sirt7*^*-/-*^ testes and were significantly higher than in WT controls. Quantification of PLZF⁺ spermatogonia in individual seminiferous tubules showed a marked reduction in quantity after doxorubicin treatment in *Sirt7*^*-/-*^ testes, whereas there was no significant change in WT testes (Fig. 5c). Together, these results indicate that loss of SIRT7 leads to increased DNA damage and reduced spermatogonia survival under external genotoxic stress.

Treatment of GC-2spd(ts) cells with doxorubicin increased γH2AX signal accumulation in both genotypes, but, importantly, *Sirt7*^*-/-*^ cells showed higher levels of γH2AX compared with WT controls (Supplementary Fig. 5a, b). Notably, reintroduction of SIRT7 in *Sirt7*^*-/-*^ GC-2spd(ts) cells significantly reduced γH2AX signal (Fig. 5d, e), linking SIRT7 activity to the prevention of DSB accumulation upon environmental stress.

Genome damage accumulates during aging, with environmental stress being a major contributing factor^47^. Given the critical role of SIRT7 in limiting DSB formation, we hypothesized that SIRT7 sustains genome integrity in spermatogonia during aging. We analyzed spermatogonia in 2–3- and 9–12-month-old WT and *Sirt7*^*-/-*^ testes. Examining PLZF^+^ cells in 2–3-month-old WT and *Sirt7*^*-/-*^ testis sections revealed no significant changes in undifferentiated spermatogonia frequencies (Fig. 5f, g). In 9–12-month-old testes, however, there was a significant decline in the frequency of undifferentiated spermatogonia per tubule in *Sirt7*^*-/-*^ testes compared with WT controls (Fig. 5f, g). Further analysis of DSB accumulation in WT and *Sirt7*^*-/-*^ testis revealed significant accumulation of γH2AX in undifferentiated spermatogonia with age, and this was more pronounced in a fraction of *Sirt7*^*-/-*^ cells (Fig. 5h, i). Collectively, these findings highlight an important role for SIRT7 in maintaining the population of undifferentiated spermatogonia during aging.

### SIRT7 loss impairs meiosis in an age-dependent manner

Given the premature decline of undifferentiated spermatogonia in 9–12-month-old *Sirt7*^*-/-*^ testes, and the accompanying increased DSB accumulation, we investigated the extent to which successive spermatogenic stages were affected by the lack of SIRT7. Using co-immunostaining of γH2AX, SYCP3, a lateral element protein of the synaptonemal complex^48^, and H1T, a testis-specific linker histone that is expressed from mid-pachynema onwards^49^, we identified and quantified the distinct prophase I stages in WT and *Sirt7*^*-/-*^ testes (Supplementary Fig. 6a). In WT spermatocytes, the γH2AX signal is dispersed during leptonema and zygonema, coinciding with DSB formation and the onset of chromosome pairing. In early pachynema, autosomal SYCP3 axes are largely synapsed, although discrete γH2AX foci persist on some autosomes. At this stage, the sex chromosomes initiate synapsis through the pseudoautosomal region (PAR), which exhibit a strong γH2AX enrichment, marking the onset of the sex body formation^50^. The developing sex body frequently displays an elongated configuration at this point. In mid pachynema, H1T expression is detectable for the first time and is further increased in late pachynema. Additionally, in mid and late pachynema, γH2AX disappears from autosomes and remains only in the sex body, displaying a rounded configuration and promoting meiotic sex chromosome inactivation^51^. Finally, diplotene cells are identified by the appearance of discontinuous SYCP3 staining due to chromosome desynapsis, together with strong SYCP3 signal persisting at chromosome ends.

**Fig. 6:**
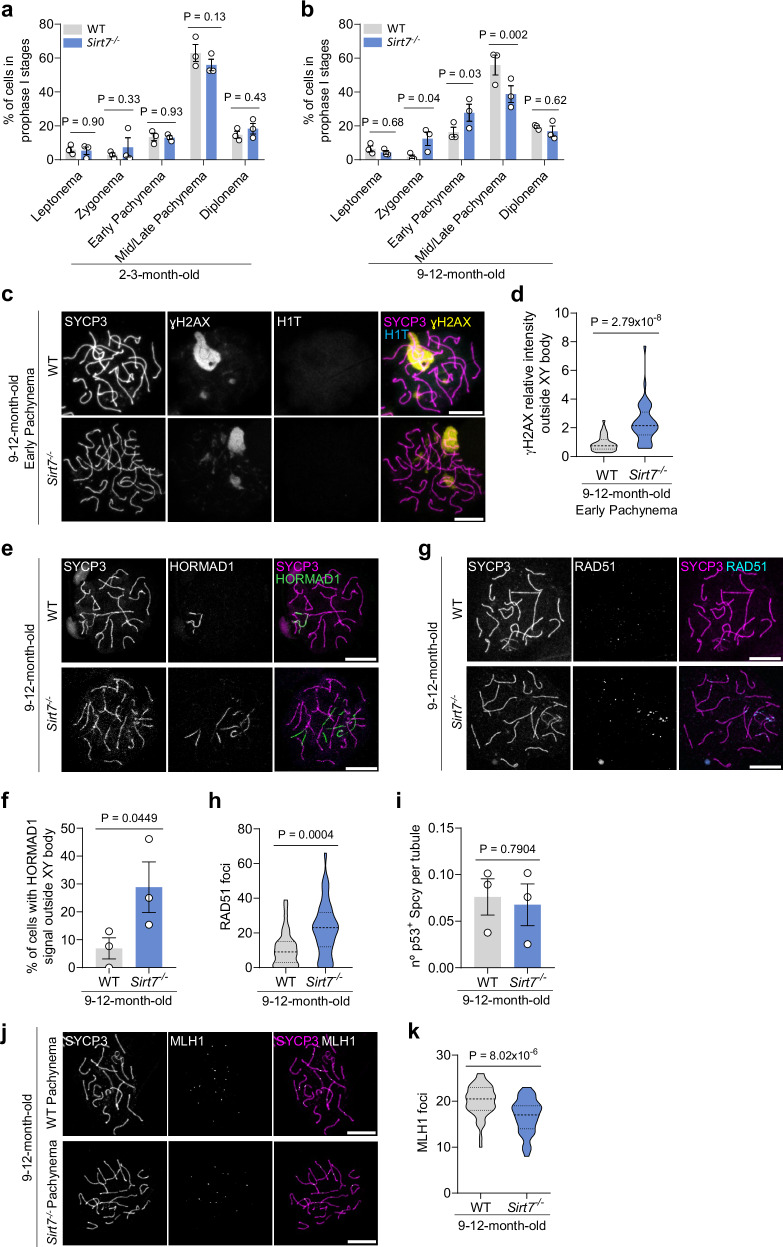
SIRT7 deficiency delays meiotic progression and reduces crossovers in older mice. **a**, **b** Percentages of cells in meiotic prophase I stages from 2–3- (**a**) and 9–12-month-old (**b**) 129S1/Sv WT and *Sirt7*^*-/-*^ testes determined by SYCP3, ɣH2AX, and H1T immunostaining. *n* = 3 biological replicates, 100 cells per sample were scored. Bar charts and error bars indicate mean ± SEM. Two-way ANOVA with uncorrected Fisher’s LSD tests. **c** SYCP3, ɣH2AX, and H1T immunostaining in 9–12-month-old WT and *Sirt7*^*-/-*^ early pachynemas. Scale bar, 10 µm. **d** Relative ɣH2AX signal intensity quantification outside the sex body in 9–12-month-old WT and *Sirt7*^*-/-*^ early pachynemas. ɣH2AX intensity of *Sirt7*^*-/-*^ samples was normalized to its WT control to eliminate technical bias. Three biological replicates, individual cell measurements shown. WT (*n* = 39 cells), *Sirt7*^*-/-*^ (*n* = 42). Two-tailed t-test. **e** SYCP3 and HORMAD1 immunostaining in 9–12-month-old WT and *Sirt7*^*-/-*^ pachynemas. Three biological replicates. Scale bar, 10 µm. **f** Percentage of pachynemas with HORMAD1 signal outside XY bivalent in 9–12-month-old WT and *Sirt7*^*-/-*^ samples. *n* = 3 biological replicates, more than 20 pachynemas were scored per sample. Bar plots show mean ± SEM. One-tailed t-test. **g** Representative images of SYCP3 and RAD51 immunostaining in 9–12-month-old WT and *Sirt7*^*-/-*^ pachynemas. Three biological replicates. Scale bar, 10 µm. **h** RAD51 foci quantification in 9–12-month-old WT and *Sirt7*^*-/-*^ pachynemas. Three biological replicates, individual cell measurements shown. WT (*n* = 35 cells), *Sirt7*^*-/-*^ (*n* = 40). Two-tailed t-test. **i** Mean number of p53^+^ spermatocytes per tubule in 9–12-month-old WT and *Sirt7*^*-/-*^ testicular sections. *n* = 3 biological replicates with more than 50 tubules per sample scored. Bar plots show mean ± SEM. Two-tailed t-test. **j** SYCP3 and MLH1 immunostaining in 9–12-month-old WT and *Sirt7*^*-/-*^ pachynemas. Three biological replicates. Scale bar, 10 µm. **k** Quantification of MLH1 foci in 9–12-month-old WT and *Sirt7*^*-/-*^ pachynemas. Three biological replicates, *n* = 50 cells/group. Two-tailed t-test. Source data are provided as a Source data file.

Chromatin spreads from 2-3-month-old WT and *Sirt7*^*-/-*^ testes had similar percentages of leptotene, zygotene, early and mid/late pachytene, and diplotene spermatocytes (Fig. 6a). In contrast, 9–12-month-old *Sirt7*^*-/-*^ testes had a significant increase in zygotene and early pachytene spermatocytes and a decrease in mid/late pachytene spermatocytes compared with WT controls, suggestive of a developmental delay in the absence of SIRT7 (Fig. 6b). Notably, while γH2AX intensity signal was similar between 9-12-month-old WT and *Sirt7*^*-/-*^ spermatocytes in zygonema, (Supplementary Fig. 6b), γH2AX intensity signal outside the sex body in early pachytene *Sirt7*^*-/-*^ spermatocytes was significantly increased compared to WT controls, suggesting defects in homologous chromosome synapsis or meiotic recombination (Fig. 6c, d). Consistently, further analyses of pachytene spermatocytes in aged *Sirt7*^*-/-*^ samples revealed increased levels of HORMAD1^48^, a protein that binds to unsynapsed chromosome axis (Fig. 6e, f), and an increase in RAD51 recombinase foci (Fig. 6g, h). The central element protein SYCE1^48^ localized along SYCP3-fully synapsed autosomes and within the PAR of sex chromosomes in both WT and *Sirt7*^*-/-*^ spermatocytes, indicating that the assembly of the synaptonemal complex was not compromised (Supplementary Fig. 6c). Spermatocytes harboring meiotic recombination defects may undergo a p53-dependent arrest in pachynema prior to the onset of H1T expression, resulting in the selective elimination of cells carrying persistent DSBs^52^. Analysis of p53 immunostaining in testis sections from 9-12-month-old WT and *Sirt7*^*-/-*^ mice showed similarly low numbers of p53-positive cells (Fig. 6i and Supplementary Fig. 6d), indicating that the meiotic recombination checkpoint is not activated in the absence of SIRT7. Indeed, the number of spermatocytes in diplonema was similar in the two genotypes (Fig. 6b), suggesting that *Sirt7*^*-/-*^ spermatocytes undergo a developmental delay during late zygonema and early pachynema but eventually progress to later prophase I stages. Intriguingly, MLH1 immunostaining revealed fewer crossing-over foci in 9–12-month-old *Sirt7*^*-/-*^ cells (Fig. 6 j, k). Overall, our results suggest that the absence of SIRT7 results in a prophase I developmental delay and reveal an impairment in the resolution of DSBs as crossovers in aged *Sirt7*^*-/-*^ spermatocytes. Additionally, our results suggest that SIRT7 is a key regulator of chromatin structure and genome stability in both spermatogonia and spermatocytes, with important consequences for male reproductive longevity.

## Discussion

In this study, we analyzed the effects of *Sirt7* deletion on mouse spermatogenesis. Our results demonstrate that *Sirt7*^*-/-*^ male mice exhibited an age-dependent subfertility characterized by early onset of defects typically occurring during natural aging in mice^25^. These defects included age-dependent decreased fecundity (fewer and smaller litters), increased histological abnormalities in seminiferous tubules, and impaired spermatogenesis. Further investigation revealed constitutive H3K36ac epigenetic abnormalities in spermatogonia and spermatocytes associated with changes in chromatin structure and an increase in DSB accumulation.

Our work helps fill the gap of knowledge on the roles of sirtuins in spermatogenesis. Our analysis of publicly available transcriptomic data on human^34,53^ and mouse^35^ testes revealed distinct sirtuin expression patterns that suggest important roles during spermatogenesis. In human testes, *Sirt7* transcripts were the most abundant of all sirtuins in both spermatogonia and spermatocytes (Supplementary Fig. 3a, b). In mice, *Sirt7* was amongst the highest expressed sirtuins in undifferentiated spermatogonia together with *Sirt1*, *Sirt2,* and *Sirt3,* suggesting different requirements for sirtuin activity between humans and mice.

We found that SIRT7 was highly expressed in spermatogonia and spermatocytes during the first wave of spermatogenesis and then sharply declined as spermatids appeared (Fig.3a, b, and Supplementary Fig. 3e), a pattern also observed in adult testes scRNA‑seq data (Supplementary Fig. 3a–c). Whether SIRT7 protein levels fluctuate in early germ cells during the first and subsequent waves of spermatogenesis will require further investigation. SIRT7 directly deacetylates H3K36ac and H3K18ac in somatic cells^23,28^. Although global levels of H3K18ac did not change upon SIRT7 deletion in testes, we observed a significant increase in H3K36ac regardless of age (Fig. 2). Further analysis of testicular sections enabled us to identify SIRT7‑dependent H3K36ac changes in specific germ cell populations (Fig. 3c–e) which were not detectable in bulk whole‑testis lysates (Fig. 2). Consistent with H3K36ac being a bona fide substrate of SIRT7^23^, H3K36ac levels were inversely correlated with SIRT7 levels, being low in spermatogonia and early spermatocytes, including in leptonema and zygonema of prophase I, showing a mild increase during pachynema and diplonema, and being high in round spermatids, where SIRT7 protein levels were the lowest (Fig. 3a). Notably, aging in WT testes led to an increase in H3K36ac in spermatogonia and pachytene spermatocytes (Fig. 3e and Supplementary Fig. 3i, j). Further research should directly investigate the role of SIRT7-dependent H3K36ac deacetylation in the testes of naturally aged mice. Because previous work showed stage-specific changes in H3K27ac in male germ cells during aging^54^, our results suggest age-related histone acetylation alterations vary between different histone lysine residues.

In somatic cells, H3K36ac is prominently enriched at gene promoters and has been proposed to modulate transcription^43^. Here we also found a strong enrichment of H3K36ac in the promoters of WT and *Sirt7*^*-/-*^ undifferentiated spermatogonia (Supplementary Fig. 4b). Despite dramatically higher H3K36ac levels, analyses of the transcriptomes did not indicate a preference for gene expression upregulation in *Sirt7*^*-/-*^ spermatogonia (Supplementary Fig. 4c), which suggests that this epigenetic mark plays a role beyond transcriptional regulation. Previous work in *Saccharomyces cerevisiae* established a positive correlation between H3K36ac and chromatin digestibility after genotoxic damage^30^. Consistent with this finding, our data identify altered chromatin accessibility in primary spermatogonia and in a germ cell-derived cell line lacking SIRT7 (Fig. 4c, d, and Supplementary Fig. 4g–j), which raises the possibility that SIRT7 loss compromises chromatin dynamics in early-stage germ cells. Notably, forced expression of a constitutively H3K36 acetylated mutant (H3^K36Q^) resulted in enhanced chromatin digestibility, establishing a direct link between this epigenetic modification and chromatin openness (Fig. 4f–h). Our results also suggest that H3K36ac influences nucleosome stability, but future experiments are necessary to identify the molecular mechanisms by which this histone mark affects chromatin structure. Studies using human mesenchymal stem cells showed that SIRT7 promotes chromatin compaction through H3K9me3 regulation, indicating that SIRT7 may regulate chromatin accessibility through various epigenetic mechanisms^55^.

We found that testis aging in WT animals was associated with increased DNA damage in undifferentiated spermatogonia, and that this effect was exacerbated in *Sirt7*^*-/-*^ testes (Fig. 5). Since undifferentiated spermatogonia include the SSC pool^8^, which sustains steady-state spermatogenesis, the premature reproductive decline in *Sirt7*^*-/-*^ males may be linked to defects in this population. Indeed, our findings align with previous studies in somatic tissues, which describe an association of cumulative stem cell genome damage with age-related physiological decline^56^. In support of this concept, SIRT7 is described as playing key roles in hematopoietic^57^ and hair follicle^58^ stem cells, suggesting a broader role for SIRT7 in safeguarding stem cell homeostasis. SIRT7 deficiency also results in age-dependent spermatogenic defects (Fig. 6) and DNA-damaged sperm (Fig. 1h–j). However, further investigations will be essential to dissect the distinct contributions of H3K36ac deregulation and genome instability arising specifically in spermatogonia versus spermatocytes to the overall aging phenotype. Additionally, despite Sertoli cell numbers being normal in the absence of SIRT7 (Supplementary Fig. 1c, d), whether functional defects in this cell population may contribute to the described phenotype is currently unknown.

Although no differences in undifferentiated spermatogonia were observed in 2–3-month-old WT and *Sirt7*^*-/-*^ male mice (Fig. 5f, g), acute exposure to doxorubicin resulted in increased genome damage accumulation in *Sirt7*^*-/-*^ neonatal spermatogonia, suggesting that SIRT7 has an important role in preventing genome damage upon environmental stress in this cell population. Notably, reintroduction of SIRT7 into *Sirt7*^*-/-*^ GC-2spd(ts) cells reduced γH2AX formation in the presence of external genotoxins (Fig. 5d, e, and Supplementary Fig. 5), reinforcing the direct link between SIRT7 in genome maintenance. In somatic cells, SIRT7 participates in DSB repair through H3K18ac deacetylation^24^, which favors the recruitment of DNA repair factors at break sites. In yeast, H3K36ac favors homology recombination-mediated repair^30^, but whether it plays a similar role in mammalian cells is unknown. Previous studies indicate that timely regulation of chromatin accessibility is critical for efficient DSB repair^59^. In our work, SIRT7 deficiency resulted in elevated H3K36ac levels (Figs. 2 and 3) and altered chromatin accessibility (Fig. 4). However, additional studies will be required to determine how H3K36ac‑associated chromatin opening influences DSB repair efficiency and genome stability in spermatogonia. Additionally, because this study relied on neonatal spermatogonia for mechanistic analyses, future studies should investigate whether SIRT7-dependent H3K36ac regulation differs in adult spermatogonia.

Our results show an increase in DSBs in the sperm of aged *Sirt7*^*-/-*^ mice (Fig. 1h–j). Mature spermatozoa lack the capacity to repair DNA damage as a result of the progressive transcriptional decline in spermiogenesis and the particular structural arrangement of the nucleus^60^. Therefore, the damage observed in aged *Sirt7*^*-/-*^ spermatozoa may derive from genomic instability due to defective meiotic crossover formation, or potential impairments in chromatin remodeling or DNA damage repair pathways in spermatids where H3K36ac is slightly deregulated in *Sirt7*^*-/-*^ testis (Fig. 3d, e). In this regard, studies in humans suggest that the epididymal environment might promote DSB in sperm, particularly if the chromatin has not been properly remodeled and compacted during spermatogenesis^61^. Understanding how SIRT7 and H3K36ac might be involved in the later steps of spermatogenesis will need further study.

Considered as a whole, our results show that SIRT7 is an important epigenetic factor in regulating testicular aging. Our findings indicate that SIRT7 has crucial roles in spermatogonia and spermatocytes, where it regulates H3K36ac and genome maintenance, thereby ensuring a sustained supply of high-quality gametes as an individual age. Our results have important implications for potential interventions aimed at mitigating the age-related decline of male reproductive capacity.

## Methods

### Ethics statement

Our research complies with relevant ethical regulations. All animal experiments were conducted in accordance with institutional and national guidelines and regulations and were approved by the Ethics Committee on Animal Experimentation of the Catalan Government (protocol #10472) and the Rutgers Institutional Animal Care and Use Committee (IACUC; protocol #201702497). No additional ethical approval was required for other aspects of this study.

### Animals and sample collection

The generation of *Sirt7*^*-/-*^ mice on a 129S1/Sv genetic background has been previously described^24^. Animals were bred in the animal facilities of the Comparative Medicine and Bioimage Center (CMCiB) of the Germans Trias i Pujol Research Institute (IGTP) and Rutgers University, respectively. Mice were housed under controlled conditions with a temperature maintained at 21–25 °C, relative humidity of 40–70%, and a 12-h light/dark cycle, including a 15-min gradual light intensity ramp to simulate sunrise and sunset. All animal procedures were approved by the Animal Care and Ethics Committee of the institutions where they were carried out. Experiments were performed on male mice aged 5, 9, 18, 24, and 35 post-natal days, and 2–3, 4–6, and 9–12 months. They were humanely euthanized by CO_2_ inhalation followed by cervical dislocation and reproductive tissues dissection. As this study focuses on spermatogenesis, the majority of experiments were conducted using male mice, except for fertility trials, in which both sexes were used.

### Fertility trials

For fertility assessments in *Sirt7*^*-/-*^ males, 129S1/Sv WT and *Sirt7*^*-/-*^ males were caged singly with 129S1/Sv WT single females and co-housed until the end of the experiment. Animals were 2 months old when the study started. The total number of litters and pups per litter were recorded until the control matings produced seven litters. To assess the changes in WT male fertility with age, either 2- or 12-month-old WT males were caged with 2-month-old WT females and co-housed until the young controls had produced three litters.

### Histological analyses

Testes were dissected, rinsed in 1X PBS, and fixed in Bouin’s Solution (Sigma-Aldrich, HT10132) overnight at 4 °C. The following day, samples were washed and dehydrated before paraffin embedding using standard methods. Five-micrometer sections of paraffin-embedded testis were stained with PAS-hematoxylin using standard methods and coverslips mounted with DPX Mountant (Sigma-Aldrich, 06522). Images were acquired on an Olympus BX53 microscope equipped with an Olympus SC180 camera. Testis were classified according to the degree of affectation as follows: healthy, > 80% of with normal progression of spermatogenesis; mildly affected, > 20% of seminiferous tubules with isolated vacuolation in seminiferous epithelia (without depletion of germ cells); severely affected, >  20% of seminiferous tubules with severe vacuolation and/or complete abrogation of spermatogenesis.

### Germ cell counting

Whole testis cross-sectional PAS-H-stained images were analyzed with QuPath (version 0.5.1) as previously described^62^. Seminiferous tubules were manually outlined using the brush tool. Default cell detection settings were applied, except for the threshold, which was adjusted between 0.06 and 0.17. Shape features, including minimum and maximum diameters, were calculated. To correct for variation in tubule shape, the number of cells per tubule was normalized by multiplying the raw value by the ratio of the minimum to maximum tubule diameter.

### Sperm comet assay

Neutral comet assays were performed to detect DSBs, as previously described^14^, with technical adaptations for sperm. Briefly, sperm suspensions obtained from the cauda epididymis were spun for 4 min at 600 × *g* and pellets were washed in 1X PBS and resuspended at a concentration of 2.5 × 10^6^ cells/ml. Ten microliters of this suspension were diluted into 100 µl of 0.05% low melting point (LMP) agarose (Lonza, #50081) and spread in a uniform layer on a Superfrost Plus glass slide (Epredia J1800AMNZ) previously coated with a layer of 1.75% agarose (Lonza, 50004). Cells were lysed in two steps. First, slides were incubated in lysis buffer (2.5 M NaCl, 100 mM EDTA, 10 mM Tris, 1% Sarkosyl, 1% Triton X-100, pH 10) supplemented with 40 mM DTT for 1 h at room temperature (RT), and then in lysis buffer supplemented with 0.2 mg/ml proteinase K (Apollo Scientific, BIP4205) at 37 °C overnight. The slides were then washed in TBE buffer (90 mM Tris, 90 mM boric acid, 2 mM EDTA, pH 8.5) and subjected to electrophoresis (0.6 V/cm) in TBE for 25 min. Slides were stained with Hoechst (Thermo Scientific, 62249) before microscopic analyses. Comet images were captured on an Olympus BX51 fluorescence microscope equipped with an Olympus DP73 camera and analyzed using CometScore software (TriTek Corp.). As recommended^63^, comets with “hedgehog morphology”, indicative of sperm nuclei with highly damaged DNA, were not included in the determination of “% DNA in comet tail” and were quantified separately. The presence of these cells was represented as the percentage of the total sperm nuclei analyzed and labeled as “% highly damaged sperm”.

### Spermatogonia enrichment

Testes from 129S1/Sv WT and *Sirt7*^*-/-*^ Pnd 5 mice were decapsulated before proceeding to a two-step enzymatic digestion. Briefly, decapsulated testes were incubated in 1 mg/ml collagenase I (Millipore, SCR103), α-MEM (Gibco, 22571) for 10 min at 37 °C in a rocker to obtain individual testis cords. After precipitating testes cords, the supernatant containing interstitial cells was removed and the cords were resuspended in α-MEM containing 1 mg/ml collagenase I, 6 µg/ml trypsin (ThermoFisher, 25200056), 2 mM EDTA, 16 μg/ml hyaluronidase (Sigma, H3506), 0.4 μg/ml DNAse I (Sigma, DN25) and 0.2 μM NaPyr (Biowest, L0642) and incubated in a rocker for 10 min at 37 °C. Digestion was terminated by adding KSR (ThermoFisher, 10828010) to 4%. Differential plating is a common procedure for enriching for spermatogonia, as germ cells and somatic cells have differential adhesion rates^40,41^. Testicular cells were plated in 10 cm cell culture dishes in α-MEM, 10% KSR. Spermatogonia-enriched supernatant was collected after 3 h at 37 °C in an atmosphere of 5% CO_2_. The purity of the spermatogonia-enriched cell suspension was confirmed by flow cytometry detection of PLZF. Cells were counted and used for subsequent applications.

### Flow cytometry

Fifty thousand cells were washed once with ice-cold staining buffer (4% FBS, 2 mM EDTA in PBS). Cell fixation and permeabilization were performed with FoxP3 fixation/permeabilization buffer (eBioscience, 00-5523-00) following the manufacturer’s instructions. Briefly, cells were fixed for 30 min at room temperature and washed once in permeabilization buffer. Cells were blocked by supplementing permeabilization buffer with 1% FBS and stained with anti-PLZF antibody (Abcam, ab189849 1:1400). Cells were washed once and further incubated with an anti-IgG (H + L) secondary antibody (Invitrogen, 1:1200). Cells were washed once and analyzed in a FACSymphony A1 cytometer (BD Biosciences). Data analysis was performed using FlowJo Software (BD Life Sciences).

### Spermatogonial RNA isolation, sequencing, and analysis

Two hundred thousand Pnd 5 spermatogonia pellets were kept at −80 °C until all samples were available. RNA was isolated with a Maxwell RSC simplyRNA Tissue Kit (Promega, AS1340) following the manufacturer’s instructions. Samples were submitted to BGI for library construction and sequencing using the DNBSEQ Eukaryotic Strand-specific mRNA Library Preparation Kit, followed by sequencing on the DNBSEQ platform to generate paired-end 150 bp reads (PE150). Raw sequencing data were processed by using BGI’s standard filtering pipeline to remove adapter sequences, potential contaminants, and low-quality reads. High-quality clean reads were aligned to the mouse reference genome (GRCm39) using HISAT2, and gene-level counts were quantified with featureCounts. Differential expression was analyzed using DESeq2, applying the Benjamini–Hochberg correction to control the false discovery rate.

### ChIP-seq

Fifty thousand cells were processed using the truChIP Chromatin Shearing kit (Covaris, 520154) with modifications. Cells were washed (4500 × *g*, 1 min, 4 °C) once in PBS and fixed in 400 µL 1X buffer A containing 1% methanol-free formaldehyde for 10 min with gentle rotation. The reaction was stopped with 21 µL of quenching buffer E and 100 µL PBS-0.5% BSA, followed by a 5-min incubation in a rotator. Fixed cells were centrifuged and washed once with 1.5 mL ice-cold PBS-0.5% BSA. Fixed cell pellets were stored at −80 °C until ready for use. Frozen pellets were thawed at room temperature, and cytoplasms were lysed in 300 µL ice-cold lysis buffer containing protease inhibitors. After 10 min, nuclei were centrifuged (4500 × *g*, 3 min, 4 °C) and washed once with D3 shearing buffer with protease inhibitors. Nuclei were then resuspended in 130 µL D3 buffer and sonicated for 4 min in a Covaris M220 sonicator, following the manufacturer instructions. Sheared chromatin samples were transferred to a chilled tube and diluted with 130 µL 2X dilution buffer containing protease inhibitors and 0.1% SDS. Samples were then precleared with 7 µL Pierce ChIP-grade protein A/G magnetic beads (Thermo Fisher, 26162) for 30 min with gentle rotation at 4 °C and clarified by centrifugation (10,000 × *g*, 10 min, 4 °C). Before immunoprecipitation, SNAP-ChIP K-AcylStat panel spike-in (EpiCypher, 19-3001) was added to each sample. Chromatin immunoprecipitation was performed overnight with 13 µL magnetic beads, which had been blocked beforehand with PBS, 2% BSA (10 min at RT with rotation) and incubated for 2–4 h at 4 °C with 1 µL H3K36ac antibody (230 ng/µL) (Cell Signaling, 27683) per immunoprecipitation. ChIP samples were washed twice serially with low salt wash buffer (0.1% SDS, 1% Triton, 2 mM EDTA, 20 mM Tris-HCl pH 8.0, 150 mM NaCl), high salt wash buffer (0.1% SDS, 1% Triton, 2 mM EDTA, 20 mM Tris-HCl pH 8.0, 500 mM NaCl), LiCl wash buffer (10 mM Tris-HCl pH 8.0, 250 mM LiCl, 1% NP-40, 1% sodium deoxycholate and 1 mM EDTA), low EDTA TE (10 mM Tris-HCl pH 8.0, 0.1 mM EDTA). Crosslinking was reversed in 0.1 M NaHCO_3_, 1% SDS buffer by incubating with 10 µg RNase A (ThermoFisher, EN0531) for 30 min at 37 °C, followed by 6 h at 65 °C with 50 µg proteinase K (Apollo Scientific, 39450-01-6). DNA was cleaned up with DNA Clean & Concentrator-5 (Zymo Research, D4014) and stored at −20 °C before library preparation. ChIP-Seq libraries were prepared using the NEBNext Ultra II DNA Library Prep Kit for Illumina and multiplexed NEBNext Oligos (New England Biolabs, NEB E7103), purified with SPRISelect beads (Beckman Coulter, B23317) and sequenced on a DNBSEQ-G400 platform.

### ATAC-seq

For ATAC-seq, samples were processed following a previously published protocol^64^. Briefly, transposition was conducted on 50,000 fresh Pnd 5 spermatogonia using an in-house synthesized Tn5 transposase pre-loaded with sequencing adapters compatible with the Illumina platform. The reaction was incubated for 30 min at 37 °C. Libraries were generated using NEBNext Ultra II Q5 2x kit (New England Biolabs, NEB E7103) and cleaned and size-selected with DNA Clean and Concentrator-5 Kit (Zymo Research, D4014). Library quality was assessed using Qubit fluorometry and Agilent TapeStation before sequencing.

### ChIP-seq and ATAC-seq analysis

ChIP-Seq and ATAC-Seq files were processed similarly unless otherwise specified. FASTQ files were quality-controlled with FastQC 0.12.1 and trimmed with Trim Galore 0.6.6. Trimmed reads were aligned onto the mm10 mouse reference genome using Bowtie2 2.4.4.1^65^ before sorting and filtering to retain non-duplicated, uniquely mapped reads using SAMtools 1.19.2 (MAPQ ≥ 30)^66^ and Sambamba 0.8.2^67^ ([XS] = null and not unmapped and not duplicate). BAM files were indexed using Sambamba. ChIP-Seq spike-in reads were quantified by aligning trimmed reads onto a custom mini-genome containing SNAP-ChIP K-Ac panel barcodes with Bowtie2. After sorting and indexing, barcode counts were extracted with SAMtools. Barcode counts were comparable among samples, so spike-in normalization factors were not applied in the downstream analysis. ATAC-Seq BAM files were further processed by removing mitochondrial DNA, correcting Tn5 insertions with alignmentSieve (--ATACshift) and filtered by fragment size (retaining fragments between 150 and 1000 bp). Heatmaps and bigwig files were generated using deepTools 3.5.1 “bamCoverage”. Peaks were called with MACS2 2.2.5 (broadpeaks, broad-cutoff 0.1) and annotated with ChIPseeker 1.42.1 in the R environment (4.4.2). For comparing ATAC and H3K36ac peak intensities, H3K36ac consensus peaks were computed by merging the peaks from all replicates with bedtools 2.27.1 “merge”^68^ and used as a reference to quantify ATAC and H3K36ac signal intensities with bwtool 1.0^69^ “summary”. Differential ATAC peaks were identified with DiffBind 3.16 in R.

### Testis culture

Testes from 129S1/Sv WT and *Sirt7*^*-/-*^ Pnd 5 mice were cultured as described elsewhere^45^ with minor modifications. Briefly, testes were decapsulated and cut into 1 mm^3^ pieces while kept in Leibowitz media (Gibco, 11415) supplemented with 3 mg/ml BSA (Sigma, A7906) at 37 °C. Each piece was placed on a filter disc (Merck, WHA10417301) in α-MEM, 10% KSR and kept at 37 °C, in 5% CO_2_ atmosphere. On day 2 of culture, media was supplemented with 0.1 µM doxorubicin (DOX) (Shelleckchem, S1208) and treated for 16 h in the same culture conditions. These conditions were previously reported to induce measurable DNA damage while preserving spermatogonia survival in WT tissues^39^. After treatment, pieces of testis were fixed in NBF (Sigma, HT501128) for 2 h, embedded in 3% agarose blocks and processed for further paraffin embedding.

### CRISPR Cas9-mediated knock-out of SIRT7 and clone generation

Guide crRNAs were designed using the IDT Alt-R CRISPR design tool (Supplementary Table 1). TracrRNA (IDT, 1072532), crRNAs and Cas9 nuclease (IDT, 1081058) were nucleofected into spermatocyte-derived GC-2spd(ts) cells (ATCC CRL-2196) using a Nucleofector System (Lonza, VCA-1001) following the manufacturer’s protocols. Nucleofected single cells were sorted to generate clones. WT clones were also generated by sorting single cells from the original pool. SIRT7 levels were assessed by Western blotting, and three clones of each genotype were chosen to perform experiments.

### MNase assay

Two million WT and *Sirt7*^*-/-*^ GC-2spd(ts) were pelleted, resuspended in 1 ml RSB buffer (10 mM Tris pH 7.8, 10 mM NaCl, 3 mM MgCl2, 1% NP-40, 0.5 mM DTT, 1X complete protease inhibitor (Roche, 11836170001) and incubated for 10 min at 4 °C. Nuclei were pelleted by centrifuging 30 s at maximum speed and resuspended in 400 μl of nuclear buffer (20 mM KCl, 20 mM Tris pH 8, 70 mM NaCl, 3 mM CaCl2, 1X complete protease inhibitor). Thirty units of micrococcal nuclease (MNase) (Thermo Scientific, 88216) were added to the suspension and incubated at RT. To stop the reaction at each digestion time, 50 μl of the suspension were retrieved, mixed with 3 μl EDTA 0.5 μM and cooled on ice. Digested DNA was purified, quantified, and loaded onto a 1.8% agarose gel for electrophoresis and analysis. Images were obtained with the G-BOX system (Syngene) and analysed with FIJI v.2.3.0^70^.

### Cell transfections and treatments

WT and *Sirt7*^*-/-*^ GC-2spd(ts) (ATCC CRL-2196) were cultured in Dulbecco’s modified Eagle’s medium (DMEM) (Gibco, 31966-021) supplemented with 10% FBS (Gibco, 10270-106) and 1% PenStrep (BioWest, l0022-100) at 37 °C, in 5% CO_2_. Cells were transiently transfected with empty vector (EV) or SIRT7 pcDNA4T0 plasmids^24^ using 4 μg polyethylenimine (Polysciences, 23966) per μg of DNA, harvested 48 h post-transfection. For experiments with H3^WT^ and H3^K36Q^ mutant, platinum A cells (Cell Biolabs, RV-102) were transiently transfected with a pVSV-G vector encoding the viral envelope and pMSCV vectors encoding for H3^WT^ or H3^K36Q^ using PEI. Retroviral media was used to infect GC-2spd(ts) for 48 h following selection with 2 μg/ml Puromycin. For genotoxic treatments, culture media was supplemented with 0.1 µM DOX. In the case of transfected cells, treatments were added 24 h after transfection. In all cases, cells were washed and pelleted for protein extraction or processed for immunostaining 24 h after.

### Protein extraction

To obtain testicular protein lysates, testes were dissected, snap-frozen in liquid nitrogen, and stored at −80 °C until processing. Frozen testes were mechanically homogenized using a polytron homogenizer (Inycom, PT 1200 E) in cold RIPA buffer (50 mM Tris pH 7.8, 150 mM NaCl, 0.5% deoxycholic acid, 0.1% SDS, 1% NP-40, 1X complete protease inhibitor (Roche, 11836170001) and sonicated. For GC-2spd(ts) protein lysates, cellular pellets were lysed in cold RIPA buffer and sonicated.

### SDS-PAGE and Western blot

Protein lysates were mixed with 5X Laemmli sample buffer (2% SDS, 10% glycerol, 60 mM Tris, pH 6.8, 0.01% bromophenol blue) supplemented with 10% 2-mercaptoethanol (Sigma-Aldrich, M3148), and boiled at 95 °C for 5 min. Protein extracts were separated by SDS-PAGE (15% polyacrylamide) and then transferred to nitrocellulose membrane in transfer buffer (200 mM glycine, 25 mM Tris-HCl, 0.1% SDS, 20% methanol). Membranes were blocked with 5% (w/v) non-fat milk (in 1X PBS, 0.1% Triton X-100) and then incubated with primary antibodies (Supplementary Table 2) diluted in the same blocking solution overnight at 4 °C. Membranes were washed three times for 10 min with 1X PBS + 0.1% Tween-20 and incubated for 1 h at RT with secondary antibodies (Supplementary Table 2). After washing the membranes thrice for 10 min, antibody binding was visualized with an ECL chemiluminescence system (Thermo Fisher Scientific, SuperSignal detection kit) and exposure of the membrane to iBright Imaging Systems (Thermo Fisher Scientific). For proteins of similar molecular weight, samples were equally loaded into independent gels and each transferred to an independent membrane. Quantification of band intensity was performed using FIJI^70^. The signal intensities of the proteins of interest were normalized to those of appropriate loading controls, as detailed in the figure legends.

### Immunostaining of testicular sections

Testes were dissected and fixed overnight in 4% paraformaldehyde (PFA) at 4 °C. After three 15-min washes in H_2_O, samples were incubated overnight in 30% sucrose. Testes were then embedded in O.C.T. Compound (Tissue Tek, 4583) and frozen in dry ice. Five-micrometer sections were cut with a cryostat, placed in a Superfrost Plus slide (Epredia, J1800AMNZ) and kept at –80 °C until use. Paraffin-embedded testis pieces from testicular culture were cut in 5-µm sections and processed as follows. Antigen retrieval was performed in 10 mM sodium citrate, 0.05% Tween-20, pH 6, at 95 °C for 20 min. Slides were allowed to cool before permeabilization in 0.1% TritonX-100, 1X PBS for 10 min and further blocking in blocking buffer (0.3% BSA, 0.03% glycine, 0.01% Tween-20, 0.02% TritonX-100, 1X PBS) for 1 hr. Primary antibodies (Supplementary Table 3) were diluted in blocking buffer and incubated overnight at 4 °C. Secondary antibodies (Supplementary Table 3) were incubated for 1 h at 37 °C. Samples were counterstained with DAPI and mounted with Vectashield VIBRANCE mounting media (VectorLabs). Images were acquired with a STELLARIS 8 confocal microscope (Leica). Image analyses were performed using FIJI v.2.3.0^70^.

### Preparation of meiotic chromosome spreads

5 × 5 mm pieces of frozen testes were minced on a cold surface with a scalpel to obtain a homogeneous cell suspension. Cells were resuspended in 200 µl cold 1X PBS, and 1X complete protease inhibitor (Roche, 11836170001). Twenty-five microliters of cell suspension were placed on Superfrost Plus slides, and 80 µl of 1% lipsol detergent solution were added and distributed along the slide surface. Slides were incubated in a humid chamber for 17 min at RT. Next, 150 µl of fixative solution (1% paraformaldehyde (PFA), 0.15% Triton X-100, 1X complete protease inhibitor, pH 9.3) were added to the slides. Samples were fixed in the humid chamber for 2 h at RT and air-dried for 30 min. Finally, slides were washed twice for 2 min in 0.4% Photo-Flo (Kodak) in 1X PBS and twice for 2 min in 0.4% Photo-Flo in H_2_O. Slides were air-dried and stored at −80 °C until use.

### Immunostaining of chromosome spreads

Chromosome spreads were immunostained as previously described^22^. Briefly, slides were blocked for 40 min in blocking buffer (1X PBS, 0.5% BSA 0.1%, Tween-20). Primary antibodies (Supplementary Table 3) were diluted in blocking buffer and incubated in a humid chamber overnight at 4 °C. Slides were washed thrice for 15 min in 1X PBS, 0.1% Tween and incubated with secondary antibodies (Supplementary Table 3) diluted in blocking buffer for 1 h at 37 °C. After three 15-min washes, slides were counterstained with DAPI and mounted with Vectashield VIBRANCE mounting media (VectorLabs). Images were acquired with a STELLARIS 8 confocal microscope (Leica). Image analyses were performed using FIJI v.2.3.0^70^.

### Statistics and reproducibility

All statistical analyses were performed with GraphPad Prism 8.0.1. For datasets with less than 10 values, bar plots with overlaid individual data points indicate the mean value ± SEM (error bars). For datasets with more than 10 values, violin plots are used to represent data distribution. The mean value, first and third quartiles are indicated by dotted lines. The statistical tests performed on each dataset are specified in the corresponding figure legends. All statistical tests were performed using a 95% confidence level. Significance is shown as individual *P* values in the corresponding graphs. No statistical methods were used to predetermine sample sizes. Normal distribution of data was assumed. The number of samples analyzed, or technical replicates performed for each result, is specified in the corresponding figure legends. Briefly, fertility trials with WT and *Sirt7*^*-/-*^ were conducted with six independent matings per group. Analyses of testicular tissue (histology or immunofluorescence), immunostaining of chromosomal spreads or experiments with epididymal fluid were performed with samples from at least three independent mice per genotype and age group. In this case, individual cells were analyzed for each experimental group and were used to represent the results and perform statistical tests. For experiments in cell lines, three biological replicates were conducted from one or more independent cells clones as indicated in the figure legends. For tissue culture, individual cells were analyzed from two independent replicates. RNA-seq was performed in two independent biological replicates, and ChIP- and ATAC-seq were performed in three independent biological replicates.

### Reporting summary

Further information on research design is available in the Nature Portfolio Reporting Summary linked to this article.

## Supplementary information

Supplementary Information

Reporting Summary

Transparent Peer Review File

## Source data

Source Data

## Data Availability

The raw and processed ChIP-seq data generated in this study have been deposited in the Sequence Read Archive (SRA) and Gene Expression Omnibus (GEO) repositories under accession numbers SRP656109 and GSE314213, respectively (https://www.ncbi.nlm.nih.gov/geo/query/acc.cgi?acc=GSE314213). The ATAC-seq data have been deposited in SRA and GEO repositories under accession numbers SRP656712 and GSE314480, respectively (https://www.ncbi.nlm.nih.gov/geo/query/acc.cgi?acc=GSE314480), and the RNA-seq data have been deposited in SRA and GEO repositories under accession numbers SRP654471 and GSE313468, respectively (https://www.ncbi.nlm.nih.gov/geo/query/acc.cgi?acc=GSE313468). Sirtuin expression levels in the various testis cell populations were obtained from publicly available scRNA-seq data from young adult humans^34^ and adult mice^35^. Processed data files from adult mice were obtained from the GEO accession number GSE112393. Expression plots from human datasets were retrieved from the public online resource “Human testis Atlas” (https://humantestisatlas.shinyapps.io/humantestisatlas1/)^53^. Source data are provided with this paper.

## References

[1] Park, S. U., Walsh, L. & Berkowitz, K. M. Mechanisms of ovarian aging. *Reproduction* **162**, R19–R33 (2021). 33999842 10.1530/REP-21-0022 PMC9354567

[2] Kong, A. et al. Rate of de novo mutations and the importance of father’s age to disease risk. *Nature* **488**, 471–475 (2012). 22914163 10.1038/nature11396 PMC3548427

[3] Sharma, R. et al. Effects of increased paternal age on sperm quality, reproductive outcome and associated epigenetic risks to offspring. *Reprod. Biol. Endocrinol.* **13**, 35 (2015). 25928123 10.1186/s12958-015-0028-x PMC4455614

[4] Chico-Sordo, L. et al. Reproductive aging and telomeres: are women and men equally affected? *Mech. Ageing Dev.* **198**, 111541 (2021). 34245740 10.1016/j.mad.2021.111541

[5] Aitken, R. J. Male reproductive ageing: a radical road to ruin. *Hum. Reprod.* **38**, 1861–1871 (2023). 37568254 10.1093/humrep/dead157 PMC10546083

[6] Dong, S. et al. Testicular aging, male fertility and beyond. *Front. Endocrinol.* **13**, 1012119 (2022). 10.3389/fendo.2022.1012119 PMC9606211 36313743

[7] Ashapkin, V., Suvorov, A., Pilsner, J. R., Krawetz, S. A. & Sergeyev, O. Age-associated epigenetic changes in mammalian sperm: implications for offspring health and development. *Hum. Reprod. Update* **29**, 24–44 (2023). 36066418 10.1093/humupd/dmac033 PMC9825272

[8] Jan, S. Z. et al. Molecular control of rodent spermatogenesis. *Biochim. Biophys. Acta* **1822**, 1838–1850 (2012). 22366765 10.1016/j.bbadis.2012.02.008

[9] Huang, Y. & Roig, I. Genetic control of meiosis surveillance mechanisms in mammals. *Front. Cell Dev. Biol.* **11**, 1127440 (2023). 36910159 10.3389/fcell.2023.1127440 PMC9996228

[10] de la Fuente, R. et al. Epigenetic dysregulation of mammalian male meiosis caused by interference of recombination and synapsis. *Cells* **10**, 10.3390/cells10092311 (2021). 10.3390/cells10092311 PMC8467405 34571960

[11] Kota, S. K. & Feil, R. Epigenetic transitions in germ cell development and meiosis. *Dev. Cell* **19**, 675–686 (2010). 21074718 10.1016/j.devcel.2010.10.009

[12] Peters, A. H. et al. Loss of the Suv39h histone methyltransferases impairs mammalian heterochromatin and genome stability. *Cell* **107**, 323–337 (2001). 11701123 10.1016/s0092-8674(01)00542-6

[13] Nitahara, K. et al. Chromatin remodeler CHD8 is required for spermatogonial proliferation and early meiotic progression. *Nucleic Acids Res.*10.1093/nar/gkad1256 (2024). 10.1093/nar/gkad1256 PMC11014243 38224953

[14] Serrano, L. et al. The tumor suppressor SirT2 regulates cell cycle progression and genome stability by modulating the mitotic deposition of H4K20 methylation. *Genes Dev.* **27**, 639–653 (2013). 23468428 10.1101/gad.211342.112 PMC3613611

[15] Smirnov, D. et al. SIRT6 is a key regulator of mitochondrial function in the brain. *Cell Death Dis.* **14**, 35 (2023). 36653345 10.1038/s41419-022-05542-w PMC9849342

[16] Gamez-Garcia, A. & Vazquez, B. N. Nuclear sirtuins and the aging of the immune system. *Genes* **12**, 10.3390/genes12121856 (2021). 10.3390/genes12121856 PMC8701065 34946805

[17] Vazquez, B. N., Thackray, J. K. & Serrano, L. Sirtuins and DNA damage repair: SIRT7 comes to play. *Nucleus* **8**, 107–115 (2017). 28406750 10.1080/19491034.2016.1264552 PMC5403131

[18] Tatone, C. et al. Sirtuins in gamete biology and reproductive physiology: emerging roles and therapeutic potential in female and male infertility. *Hum. Reprod. Update* **24**, 267–289 (2018). 29447380 10.1093/humupd/dmy003

[19] Vazquez, B. N., Vaquero, A. & Schindler, K. Sirtuins in female meiosis and in reproductive longevity. *Mol. Reprod. Dev.* **87**, 1175–1187 (2020). 33184962 10.1002/mrd.23437 PMC7775317

[20] Bertoldo, M. J. et al. NAD^(+)^ repletion rescues female fertility during reproductive aging. *Cell Rep.* **30**, 1670–1681 e1677 (2020). 32049001 10.1016/j.celrep.2020.01.058 PMC7063679

[21] Isola, J. V. V. et al. A single-cell atlas of the aging mouse ovary. *Nat. Aging* **4**, 145–162 (2024). 38200272 10.1038/s43587-023-00552-5 PMC10798902

[22] Vazquez, B. N., Blengini, C. S., Hernandez, Y., Serrano, L. & Schindler, K. SIRT7 promotes chromosome synapsis during prophase I of female meiosis. *Chromosoma* **128**, 369–383 (2019). 31256246 10.1007/s00412-019-00713-9 PMC8494110

[23] Wang, W. W. et al. A click chemistry approach reveals the chromatin-dependent histone H3K36 deacylase nature of SIRT7. *J. Am. Chem. Soc.* **141**, 2462–2473 (2019). 30653310 10.1021/jacs.8b12083 PMC6812484

[24] Vazquez, B. N. et al. SIRT7 promotes genome integrity and modulates non-homologous end joining DNA repair. *EMBO J.* **35**, 1488–1503 (2016). 27225932 10.15252/embj.201593499 PMC4884211

[25] Endo, T. et al. Multiple ageing effects on testicular/epididymal germ cells lead to decreased male fertility in mice. *Commun. Biol.* **7**, 16 (2024). 38177279 10.1038/s42003-023-05685-2 PMC10766604

[26] O’Donnell, L., Smith, L. B. & Rebourcet, D. Sertoli cells as key drivers of testis function. *Semin. Cell Dev. Biol.* **121**, 2–9 (2022). 34229950 10.1016/j.semcdb.2021.06.016

[27] Petersen, C. G. et al. The effects of male age on sperm DNA damage: an evaluation of 2,178 semen samples. *JBRA Assist. Reprod.* **22**, 323–330 (2018). 30106542 10.5935/1518-0557.20180047 PMC6210622

[28] Barber, M. F. et al. SIRT7 links H3K18 deacetylation to maintenance of oncogenic transformation. *Nature* **487**, 114–118 (2012). 22722849 10.1038/nature11043 PMC3412143

[29] Moreno-Yruela, C. et al. Structural basis of SIRT7 nucleosome engagement and substrate specificity. *Nat. Commun.* **16**, 1328 (2025). 39900593 10.1038/s41467-025-56529-y PMC11790868

[30] Pai, C. C. et al. A histone H3K36 chromatin switch coordinates DNA double-strand break repair pathway choice. *Nat. Commun.* **5**, 4091 (2014). 24909977 10.1038/ncomms5091 PMC4535359

[31] Mahrez, W. et al. H3K36ac is an evolutionary conserved plant histone modification that marks active genes. *Plant Physiol.* **170**, 1566–1577 (2016). 26764380 10.1104/pp.15.01744 PMC4775133

[32] Baudat, F. et al. PRDM9 is a major determinant of meiotic recombination hotspots in humans and mice. *Science* **327**, 836–840 (2010). 20044539 10.1126/science.1183439 PMC4295902

[33] Vaquero, A. et al. SIRT1 regulates the histone methyl-transferase SUV39H1 during heterochromatin formation. *Nature* **450**, 440–444 (2007). 18004385 10.1038/nature06268

[34] Guo, J. et al. The adult human testis transcriptional cell atlas. *Cell Res.* **28**, 1141–1157 (2018). 30315278 10.1038/s41422-018-0099-2 PMC6274646

[35] Green, C. D. et al. A comprehensive roadmap of murine spermatogenesis defined by single-cell RNA-Seq. *Dev. Cell* **46**, 651–667 (2018). 30146481 10.1016/j.devcel.2018.07.025 PMC6713459

[36] Bellve, A. R. et al. Spermatogenic cells of the prepuberal mouse. Isolation and morphological characterization. *J. Cell Biol.* **74**, 68–85 (1977). 874003 10.1083/jcb.74.1.68 PMC2109873

[37] Blank, M. F. et al. SIRT7-dependent deacetylation of CDK9 activates RNA polymerase II transcription. *Nucleic Acids Res.* **45**, 2675–2686 (2017). 28426094 10.1093/nar/gkx053 PMC5389538

[38] Vazquez, B. N. et al. SIRT7 mediates L1 elements transcriptional repression and their association with the nuclear lamina. *Nucleic Acids Res.* **47**, 7870–7885 (2019). 31226208 10.1093/nar/gkz519 PMC6735864

[39] Smart, E. et al. Chemotherapy drugs cyclophosphamide, cisplatin and doxorubicin induce germ cell loss in an in vitro model of the prepubertal testis. *Sci. Rep.* **8**, 1773 (2018). 29379115 10.1038/s41598-018-19761-9 PMC5788858

[40] Xi, H. M. et al. Recent advances in isolation, identification, and culture of mammalian spermatogonial stem cells. *Asian J. Androl.* **24**, 5–14 (2022). 34135169 10.4103/aja.aja_41_21 PMC8788607

[41] Khanehzad, M. et al. MicroRNA-30a-5p promotes differentiation in neonatal mouse spermatogonial stem cells (SSCs). *Reprod. Biol. Endocrinol.* **19**, 85 (2021). 34108007 10.1186/s12958-021-00758-5 PMC8188658

[42] Costoya, J. A. et al. Essential role of Plzf in maintenance of spermatogonial stem cells. *Nat. Genet.* **36**, 653–659 (2004). 15156143 10.1038/ng1367

[43] Lu, X. et al. Loss of LOXL2 promotes uterine hypertrophy and tumor progression by enhancing H3K36ac-dependent gene expression. *Cancer Res.* **82**, 4400–4413 (2022). 36197797 10.1158/0008-5472.CAN-22-0848

[44] Lazar-Contes, I. et al. Dynamics of transcriptional programs and chromatin accessibility in mouse spermatogonial cells from early postnatal to adult life. *Elife* **12**, 10.7554/eLife.91528 (2025). 10.7554/eLife.91528 PMC11999699 40231607

[45] Lopes, F., Smith, R., Nash, S., Mitchell, R. T. & Spears, N. Irinotecan metabolite SN38 results in germ cell loss in the testis but not in the ovary of prepubertal mice. *Mol. Hum. Reprod.* **22**, 745–755 (2016). 27470502 10.1093/molehr/gaw051 PMC5099998

[46] Lobrich, M. et al. gammaH2AX foci analysis for monitoring DNA double-strand break repair: strengths, limitations and optimization. *Cell Cycle* **9**, 662–669 (2010). 20139725 10.4161/cc.9.4.10764

[47] Lopez-Otin, C., Blasco, M. A., Partridge, L., Serrano, M. & Kroemer, G. Hallmarks of aging: an expanding universe. *Cell* **186**, 243–278 (2023). 36599349 10.1016/j.cell.2022.11.001

[48] Llano, E. & Pendas, A. M. Synaptonemal complex in human biology and disease. *Cells* **12**, 10.3390/cells12131718 (2023). 10.3390/cells12131718 PMC10341275 37443752

[49] Inselman, A., Eaker, S. & Handel, M. A. Temporal expression of cell cycle-related proteins during spermatogenesis: establishing a timeline for onset of the meiotic divisions. *Cytogenet. Genome Res.* **103**, 277–284 (2003). 15051948 10.1159/000076813

[50] Page, J. et al. Inactivation or non-reactivation: what accounts better for the silence of sex chromosomes during mammalian male meiosis? *Chromosoma* **121**, 307–326 (2012). 22366883 10.1007/s00412-012-0364-y

[51] Fernandez-Capetillo, O. et al. H2AX is required for chromatin remodeling and inactivation of sex chromosomes in male mouse meiosis. *Dev. Cell* **4**, 497–508 (2003). 12689589 10.1016/s1534-5807(03)00093-5

[52] Marcet-Ortega, M. et al. p53 and TAp63 participate in the recombination-dependent pachytene arrest in mouse spermatocytes. *PLoS Genet.* **13**, e1006845 (2017). 28617799 10.1371/journal.pgen.1006845 PMC5491309

[53] Guo, J. et al. The dynamic transcriptional cell atlas of testis development during human puberty. *Cell Stem Cell* **26**, 262–276 (2020). 31928944 10.1016/j.stem.2019.12.005 PMC7298616

[54] Liao, J. et al. Transcriptomic and epigenomic profiling of young and aged spermatogonial stem cells reveals molecular targets regulating differentiation. *PLoS Genet.* **17**, e1009369 (2021). 34237055 10.1371/journal.pgen.1009369 PMC8291634

[55] Bi, S. et al. SIRT7 antagonizes human stem cell aging as a heterochromatin stabilizer. *Protein Cell* **11**, 483–504 (2020). 32504224 10.1007/s13238-020-00728-4 PMC7305295

[56] Behrens, A., van Deursen, J. M., Rudolph, K. L. & Schumacher, B. Impact of genomic damage and ageing on stem cell function. *Nat. Cell Biol.* **16**, 201–207 (2014). 24576896 10.1038/ncb2928 PMC4214082

[57] Mohrin, M. et al. Stem cell aging. A mitochondrial UPR-mediated metabolic checkpoint regulates hematopoietic stem cell aging. *Science* **347**, 1374–1377 (2015). 25792330 10.1126/science.aaa2361 PMC4447312

[58] Li, G. et al. SIRT7 activates quiescent hair follicle stem cells to ensure hair growth in mice. *EMBO J.* **39**, e104365 (2020). 32696520 10.15252/embj.2019104365 PMC7507325

[59] Burgess, R. C., Burman, B., Kruhlak, M. J. & Misteli, T. Activation of DNA damage response signaling by condensed chromatin. *Cell Rep.* **9**, 1703–1717 (2014). 25464843 10.1016/j.celrep.2014.10.060 PMC4267891

[60] Aitken, R. J. & Lewis, S. E. M. DNA damage in testicular germ cells and spermatozoa. When and how is it induced? How should we measure it? What does it mean? *Andrology* **11**, 1545–1557 (2023). 36604857 10.1111/andr.13375

[61] De Iuliis, G. N. et al. DNA damage in human spermatozoa is highly correlated with the efficiency of chromatin remodeling and the formation of 8-hydroxy-2’-deoxyguanosine, a marker of oxidative stress. *Biol. Reprod.* **81**, 517–524 (2009). 19494251 10.1095/biolreprod.109.076836

[62] Niedenberger, B. A., Belcher, H. A., Gilbert, E. A., Thomas, M. A. & Geyer, C. B. Utilization of the QuPath open-source software platform for analysis of mammalian spermatogenesis†. *Biol. Reprod.* **112**, 583–599 (2025). 39817641 10.1093/biolre/ioaf011 PMC11911557

[63] Moller, P. et al. Visual comet scoring revisited: a guide to scoring comet assay slides and obtaining reliable results. *Mutagenesis* **38**, 253–263 (2023). 37233347 10.1093/mutage/gead015

[64] Grandi, F. C., Modi, H., Kampman, L. & Corces, M. R. Chromatin accessibility profiling by ATAC-seq. *Nat. Protoc.* **17**, 1518–1552 (2022). 35478247 10.1038/s41596-022-00692-9 PMC9189070

[65] Langmead, B. & Salzberg, S. L. Fast gapped-read alignment with Bowtie 2. *Nat. Methods* **9**, 357–359 (2012). 22388286 10.1038/nmeth.1923 PMC3322381

[66] Li, H. et al. The sequence alignment/map format and SAMtools. *Bioinformatics* **25**, 2078–2079 (2009). 19505943 10.1093/bioinformatics/btp352 PMC2723002

[67] Tarasov, A., Vilella, A. J., Cuppen, E., Nijman, I. J. & Prins, P. Sambamba: fast processing of NGS alignment formats. *Bioinformatics* **31**, 2032–2034 (2015). 25697820 10.1093/bioinformatics/btv098 PMC4765878

[68] Quinlan, A. R. & Hall, I. M. BEDTools: a flexible suite of utilities for comparing genomic features. *Bioinformatics* **26**, 841–842 (2010). 20110278 10.1093/bioinformatics/btq033 PMC2832824

[69] Pohl, A. & Beato, M. bwtool: a tool for bigWig files. *Bioinformatics* **30**, 1618–1619 (2014). 24489365 10.1093/bioinformatics/btu056 PMC4029031

[70] Schindelin, J. et al. Fiji: an open-source platform for biological-image analysis. *Nat. Methods* **9**, 676–682 (2012). 22743772 10.1038/nmeth.2019 PMC3855844

