# Supplementary material for: SIRT7 links H3K36ac epigenetic regulation with genome maintenance in the mouse testis
Source: Nat Commun. 2026 Apr 28;17:5809. doi: 10.1038/s41467-026-72540-3 (PMC13328743; doi:10.1038/s41467-026-72540-3)

SUPPLEMENTARY INFORMATION

SIRT7 links H3K36ac epigenetic regulation with genome maintenance in the mouse testis

Anna Guitart-Solanes[1], Mayra Romero[2], Andres Gamez-Garcia[1], Irene Fernández-Duran[1], Bryan A. Niedenberger[3], Cristina Madrid-Sandín[4,5], Norah Spears[6], Ignasi Roig[4,5], Christopher B. Geyer[3,7], Alejandro Vaquero[1,*], Karen Schindler[2,*] and Berta N. Vazquez[1,4,*]

[1]Chromatin Biology Laboratory, Josep Carreras Leukaemia Research Institute (IJC), 08916 Badalona, Spain

[2]Department of Genetics, Rutgers University, Piscataway, NJ 08854, USA

[3]Department of Anatomy and Cell Biology, Brody School of Medicine, Greenville, NC 27834, USA

[4]Cytology and Histology Unit, Department of Cell Biology, Physiology and Immunology, Universitat Autònoma de Barcelona (UAB), 08193 Cerdanyola del Vallès, Spain

[5]Genome Integrity and Instability Group, Institut de Biotecnologia i Biomedicina, Universitat Autònoma de Barcelona, 08193 Cerdanyola del Vallès, Spain

[6]Institute of Neuroscience and Cardiovascular Research, University of Edinburgh, Edinburgh, UK

[7]East Carolina Diabetes and Obesity Institute at East Carolina University, Greenville, NC 27834 USA

***Corresponding authors:**

Berta N. Vazquez; Berta.Vazquez@uab.cat

Karen Schindler; schindler@dls.rutgers.edu

Alejandro Vaquero; avaquero@carrerasresearch.org

Table of contents:

Supplementary Figures 1-6

Supplementary Tables 1-3

Uncropped blots for Supplementary Figs. 2-5

SUPPLEMENTARY FIGURES

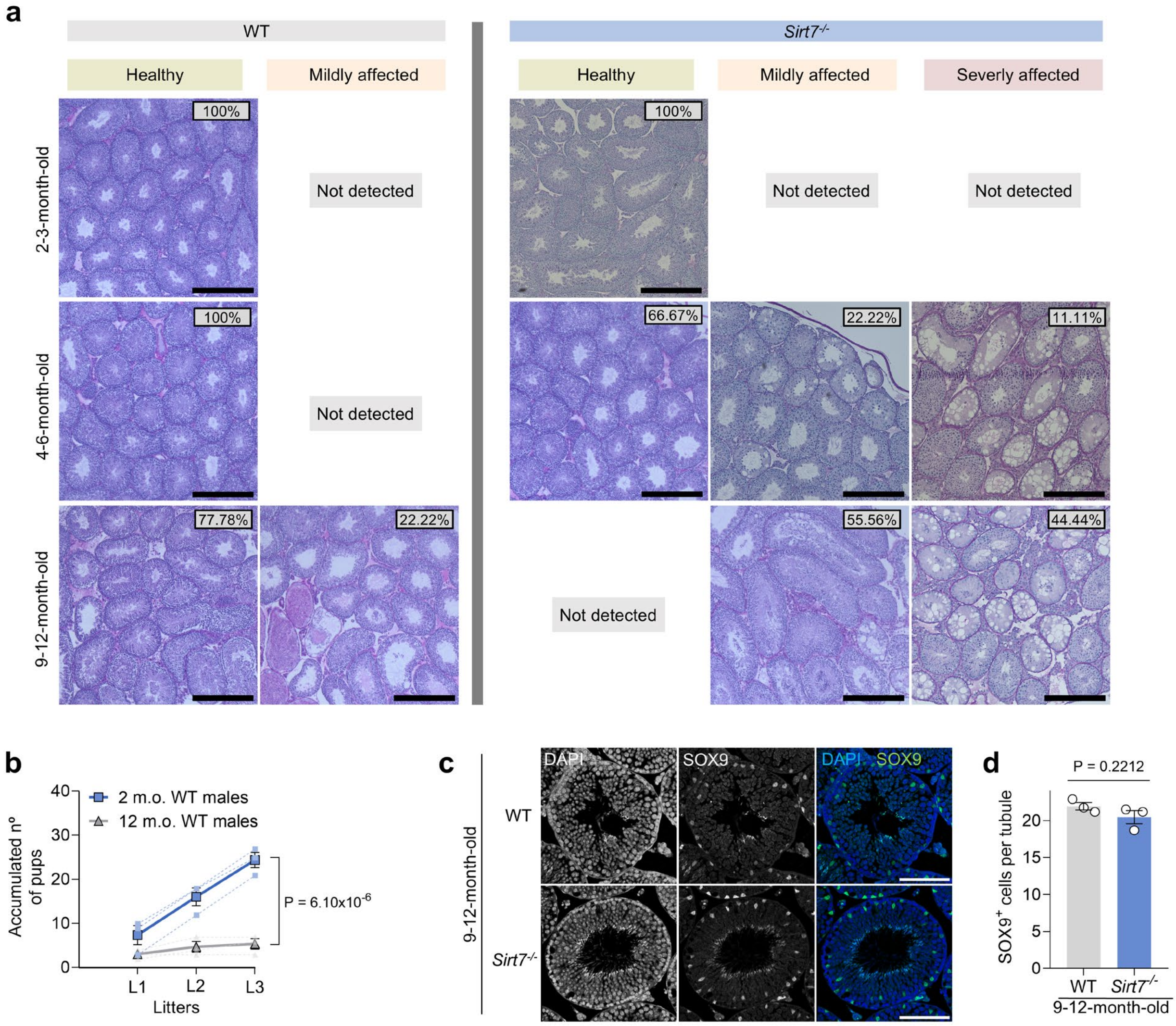

Supplementary Figure 1. Natural aging affects fertility in WT mice and to a higher extent in *Sirt7$^{-/-}$* mice

a PAS-hematoxylin-stained testis sections from WT and *Sirt7$^{-/-}$* mice at different ages. Representative images are shown for each histological category defined in the 'Methods' (healthy, mildly affected, severely affected). *n* = 9 independent testes/group. No severely affected testis was found in WT samples at any age. Scale bar, 400 μm. **b** Cumulative pup production across 3 litters (L1-L3) by WT x WT matings with 2- or 12-month-old males (*n* = 3 matings/age group). Dotted lines represent individual matings; highlighted symbols and lines indicate mean +/- SEM. Two-way repeated measures ANOVA. **c** Immunostaining for SOX9 in testis sections from 9-12-month-old WT and *Sirt7$^{-/-}$* mice. Representative images from 3 biologically independent testes/group. Scale bar, 50 μm. **d** Quantification of SOX9^{+} cells per tubule in 9-12-month-old WT and *Sirt7$^{-/-}$* testis samples (*n* = 3

independent testes/group). Each dot represents the mean of twenty analyzed tubules in each testis. Bar plots indicate mean +/- SEM. Two-tailed t-test. Source data are provided as a Source Data file.

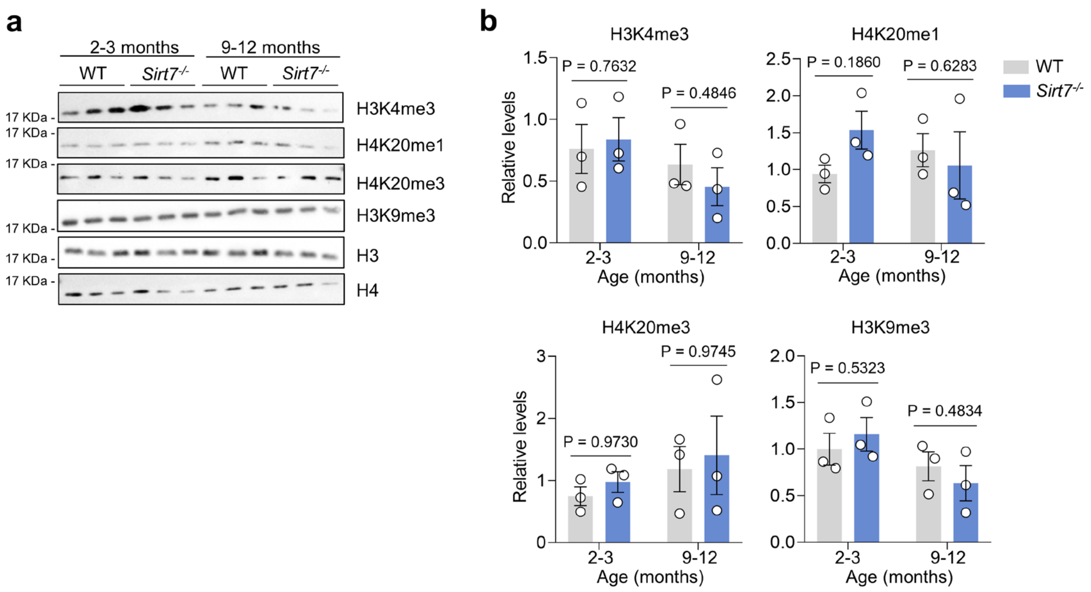

Supplementary Figure 2. Spermatogenesis and Sirtuin-linked epigenetic marks in WT and *Sirt7*$^{-/-}$ samples.

a Representative Western blot images of modified histones in whole-testis protein lysates from 2-3- and 9-12-month-old WT and *Sirt7*$^{-/-}$ mice. Each lane represents an independent testis sample. Three biologically independent testes/group were analyzed in two technical replicates, with similar results. **b)**Densitometry-based quantifications of relative epigenetic mark levels in Western blots (a), normalized to H3. n = 3 biological replicates, each represented by one dot. Bar plots and error bars show mean +/- SEM. Two-way ANOVA with uncorrected Fisher's post hoc tests. Source data are provided as a Source Data file.

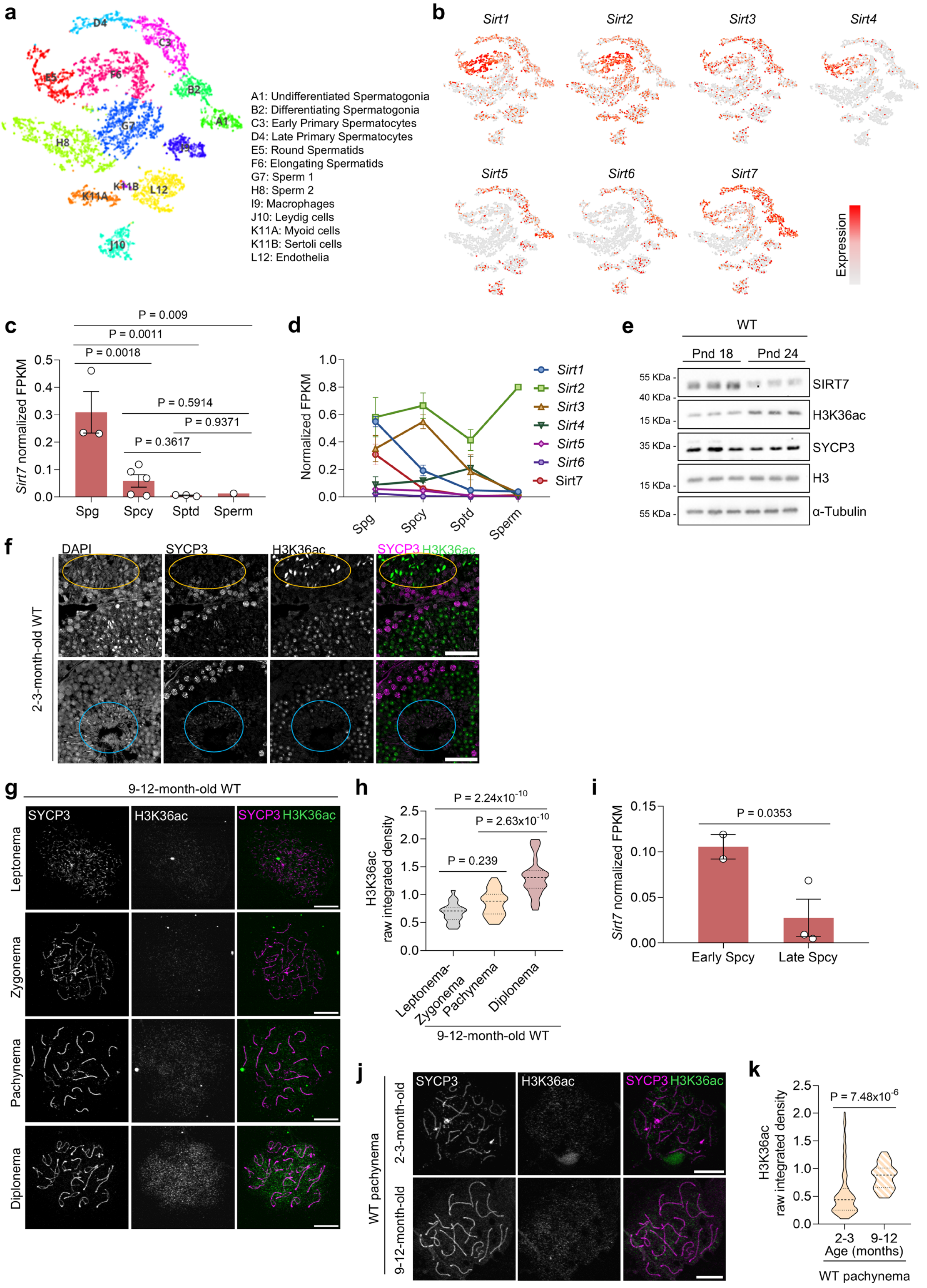

a
A1: Undifferentiated Spermatogonia
B2: Differentiating Spermatogonia
C3: Early Primary Spermatocytes
D4: Late Primary Spermatocytes
E5: Round Spermatids
F6: Elongating Spermatids
G7: Sperm 1
H8: Sperm 2
I9: Macrophages
J10: Leydig cells
K11A: Myoid cells
K11B: Sertoli cells
L12: Endothelia
b
Sirt1
Sirt2
Sirt3
Sirt4
Sirt5
Sirt6
Sirt7
Expression
c
Sirt7 normalized FPKM
P = 0.009
P = 0.0011
P = 0.0018
P = 0.5914
P = 0.9371
P = 0.3617
Spg
Spcy
Sptd
Sperm
d
Normalized FPKM
Sirt1
Sirt2
Sirt3
Sirt4
Sirt5
Sirt6
Sirt7
Spg
Spcy
Sptd
Sperm
e
WT
Pnd 18
Pnd 24
55 KDa
40 KDa
15 KDa
35 KDa
15 KDa
55 KDa
SIRT7
H3K36ac
SYCP3
H3
α-Tubulin
f
DAPI
SYCP3
H3K36ac
SYCP3 H3K36ac
2-3-month-old WT
g
9-12-month-old WT
SYCP3
H3K36ac
SYCP3 H3K36ac
Leptonema
Zygonema
Pachynema
Diplonema
h
P = 2.24x10^-10
P = 2.63x10^-10
P = 0.239
H3K36ac raw integrated density
Leptonema-Zygonema
Pachynema
Diplonema
9-12-month-old WT
i
P = 0.0353
Sirt7 normalized FPKM
Early Spcy
Late Spcy
j
SYCP3
H3K36ac
SYCP3 H3K36ac
WT pachynema
2-3-month-old
9-12-month-old
k
P = 7.48x10^-6
H3K36ac raw integrated density
2-3
9-12
Age (months)
WT pachynema

Supplementary Figure 3. H3K36ac levels in germ cells are correlated with SIRT7 levels and change with age.

a T-distributed stochastic neighbor embedding (t-SNE) plot of public scRNA-seq data showing young human testicular populations. Data obtained from https://humantestisatlas.shinyapps.io/humantestisatlas1/[34,53]. **b** tSNE plots showing sirtuin expression in human testicular populations (a). **c, d** Expression of *Sirt7* (c) and all sirtuins (d) in adult mouse spermatogenic cells. Data obtained from public scRNA-seq datasets[35]. Dots represent independent testes. Spermatogonia, Spermatids (n = 3); Spermatocytes (n = 5); Sperm (n = 1). In (c), bar plots show mean +/- SEM. One-way ANOVA with Tukey's multiple comparisons test. In (d), data shown as mean +/- SEM. **e** Western blot of SIRT7 and H3K36ac in testes from Pnd 18 and Pnd 24 WT mice. SYCP3 was used as a spermatocyte marker, and H3 and tubulin as loading controls. 3 biological replicates (independent to those in Fig. 3a), analyzed in technical duplicates with similar results. **f** SYCP3 and H3K36ac immunostaining in 2-3-month-old WT testis sections. 3 biological replicates. Orange and blue circles indicate elongating and elongated spermatids, respectively. Scale bar, 25 µm. **g** SYCP3 and H3K36ac immunostaining of prophase I chromosome spreads from 9-12-month-old WT testes. 3 biological replicates. Scale bar, 10 µm. **h** Quantification of H3K36ac fluorescence intensity in 9-12-month-old WT prophase I spermatocytes. 3 biological replicates, individual cells shown. Leptonema/Zygonema (n = 20 cells), Pachynema (n = 56), Diplonema (n = 26). One-way ANOVA with Tukey's multiple comparisons test. **i** *Sirt7* expression in early (n = 2) and late spermatocytes (n = 3) from independent adult mouse testes. Data obtained from public scRNA-seq datasets[35]. Bar plots show mean +/- SEM. One-tailed t-test. **j** SYCP3 and H3K36ac immunostaining of 2-3- and 9-12-month-old WT pachynemas. 3 biological replicates. Scale bar, 10 µm. **k** Quantification of H3K36ac fluorescence intensity in 2-3- and 9-12-month-old WT pachynemas. 3 biological replicates, individual cells shown. 2-3-month-old (n = 98 cells), 9-12-month-old (n = 56). Two-tailed t-test. In this figure: Spg, Spermatogonia; Spermatocytes, Spcy; Spermatids, Sptd. Source data are provided as a Source Data file.

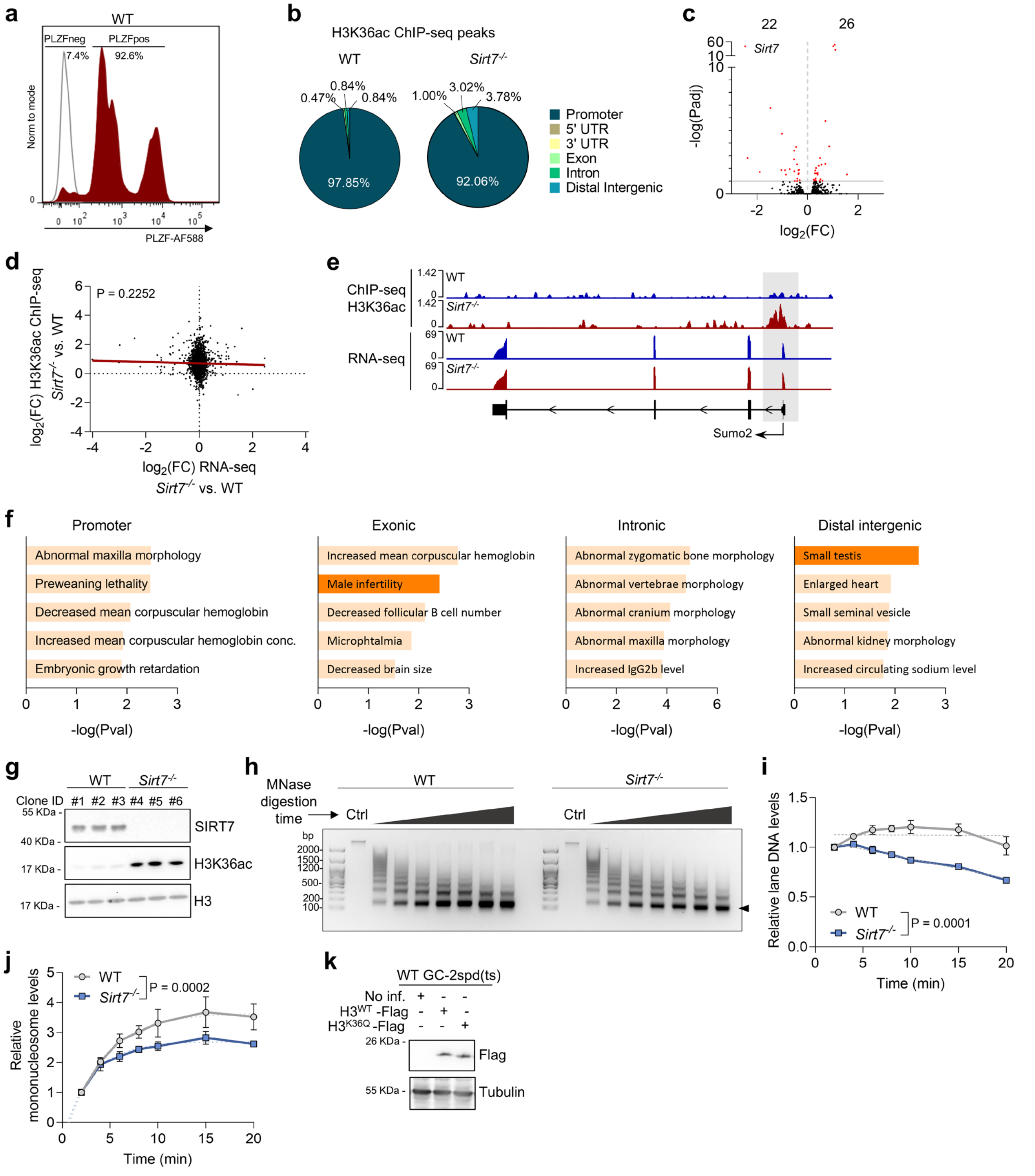

Supplementary Figure 4. *Sirt7^-/-^*-mediated H3K36ac increase affects chromatin accessibility without direct impact on transcription

a FACS histogram showing the percentage of PLZF^{+} cells after differential plating of WT Pnd 5 testicular cells, in red (PLZFpos). In white, cells stained only with secondary antibody (PLZFneg). **b** Pie chart showing genomic distribution of H3K36ac peaks from ChIP-seq in WT and *Sirt7*$^{-/-}$. **c** Volcano-plot showing differentially expressed genes (DEGs) from RNA-seq in *Sirt7*$^{-/-}$ Pnd 5 spermatogonia

relative to WT. DEGs defined using Padj < 0.1 calculated with DESeq2's Wald test without corrections for multiple comparisons. **d** Dot plot showing the correlation between gene expression changes from RNA-seq and changes in H3K36ac ChIP-seq signal in *Sirt7*$^{-/-}$ vs. WT Pnd 5 spermatogonia. Each dot represents a gene detected by RNA-seq that was associated with a differential H3K36ac peak in *Sirt7*$^{-/-}$ (defined using P < 0.05, calculated with DESeq2's Wald test without multiple comparisons correction). **e** Browser view of a representative locus (Sumo2). Top, H3K36ac ChIP-seq signal. Bottom, RNA-seq expression levels. **f** GO signatures of genes with differential ATAC signal in *Sirt7*$^{-/-}$ Pnd 5 spermatogonia sorted by genomic distribution. **g** Western blot of SIRT7 and H3K36ac levels in three WT and three *Sirt7*$^{-/-}$ GC-2spd(ts) CRISPR-Cas9-generated clones. Two technical replicates with similar results. **h** Agarose gel banding showing chromatin digestion of WT and *Sirt7*$^{-/-}$ GC-2spd(ts) cells following MNase treatment for increasing times (0, 2, 4, 6, 8, 10, 15 and 20 min). 3 replicates using independent cell clones, with similar results. Black arrowhead indicates mononucleosome-fraction bands. **i, j** Densitometry-based quantifications of chromatin digestion showing total amount of DNA at each time point (whole lane) (a) or intensity of the mononucleosome fraction (b) normalized to the amount of DNA at t = 2 min (*n* = 3 biological replicates with independent cell clones). Data shown as mean values +/- SEM. (i) Linear regression analysis with comparison of slopes between genotypes (F(1,38) = 17.96). (j) one-phase association non-linear regression analysis with comparison of fits (F(3,36) = 8.366). **k** Western blot of Flag-tag in GC-2spd(ts) WT samples control (No inf.) or infected with $H3^{WT}$-flag or $H3^{K36Q}$-flag. Source data are provided as a Source Data file.

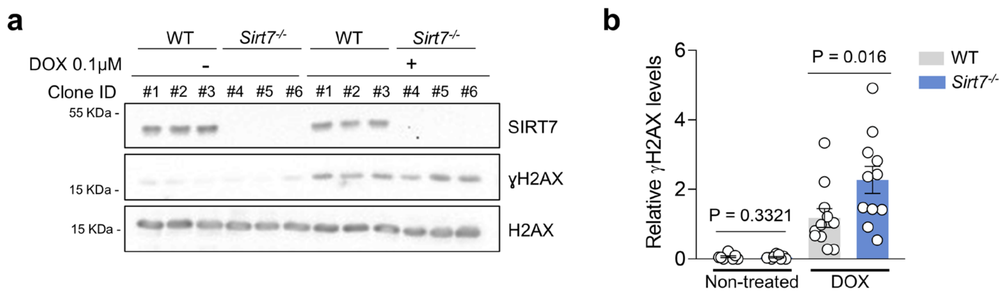

Supplementary Figure 5. DNA damage after genotoxic stress in WT and *Sirt7$^{-/-}$* GC-2spd(ts)

a Western blot of *SIRT7* and ɣH2AX in WT and *Sirt7$^{-/-}$* GC-2spd(ts) under non-treated (NT) and 0.1 µM Doxorubicin (DOX)-treated conditions. 3 biologically independent cell clones per genotype were analyzed in triplicate with similar experimental results. **b** Densitometry-based quantification of ɣH2AX levels of (a) relative to H2AX levels in Western blot. NT (*n* = 9 experimental replicates); DOX (*n* = 11). Bar plots indicate the mean value +/- SEM. One-tailed t-tests. Source data are provided as a Source Data file.

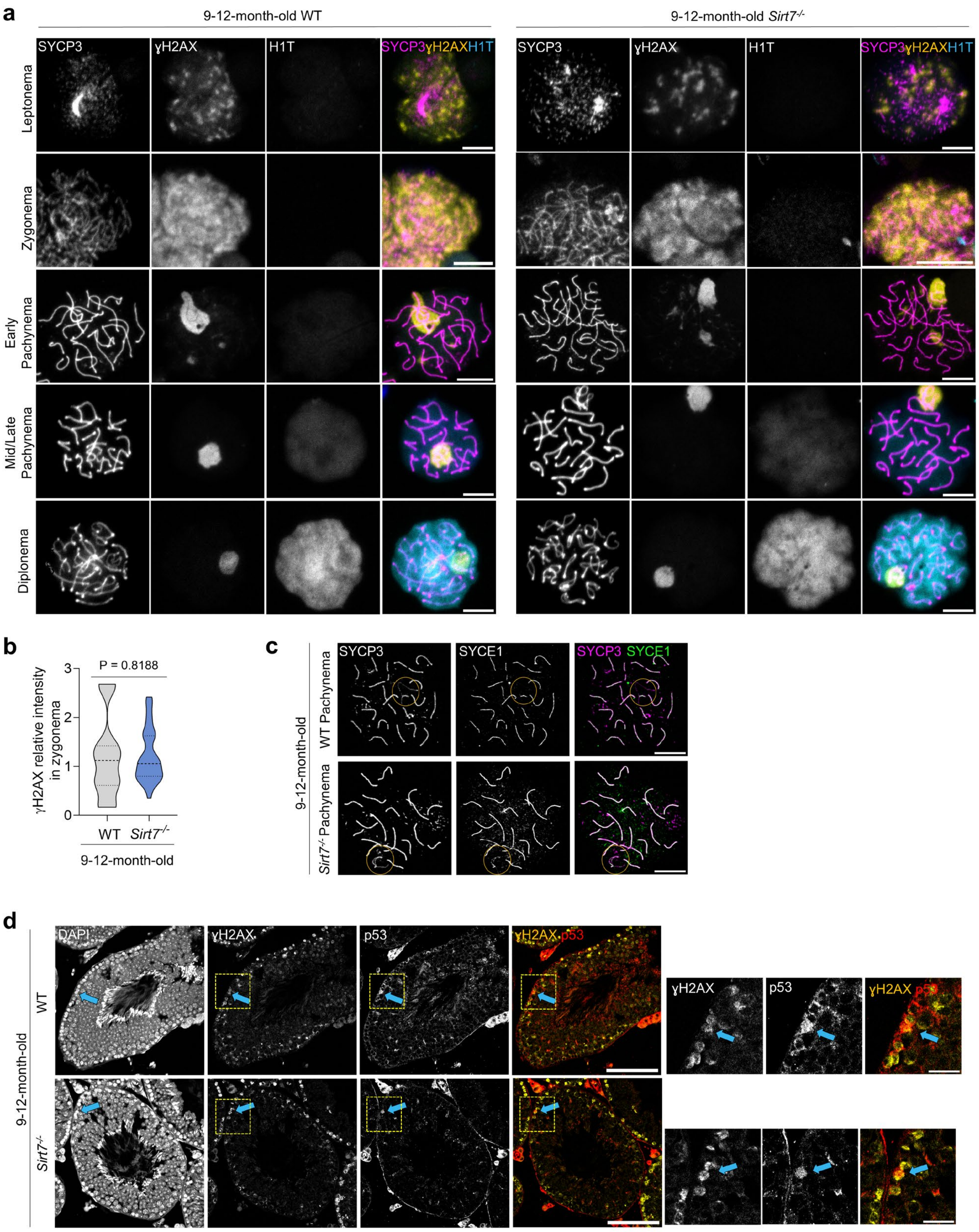

a
9-12-month-old WT
9-12-month-old Sirt7-/-
SYCP3
γH2AX
H1T
SYCP3γH2AXH1T
Leptonema
Zygonema
Early Pachynema
Mid/Late Pachynema
Diplonema
b
P = 0.8188
γH2AX relative intensity in zygonema
0
1
2
3
WT
Sirt7-/-
9-12-month-old
c
SYCP3
SYCE1
SYCP3 SYCE1
9-12-month-old
WT Pachynema
Sirt7-/- Pachynema
d
DAPI
γH2AX
p53
γH2AX p53
9-12-month-old
WT
Sirt7-/-

Supplementary Figure 6. Meiotic prophase I spermatocytes in 9-12-month-old WT and *Sirt7*$^{-/-}$ testes

a Immunostaining of SYCP3, ɣH2AX and H1T in meiotic chromosome spreads from 9-12-month-old WT and *Sirt7*$^{-/-}$ mice at different stages of the meiotic prophase I. Representative images from 3 biological replicates per genotype. Scale bar, 10 µm. **b** Quantification of the relative ɣH2AX signal intensity outside of the sex body of zygonemas from 9-12-month-old WT and *Sirt7*$^{-/-}$ mice. 3 biological replicates, individual cell measurements shown. WT (*n* = 12 cells); *Sirt7*$^{-/-}$ (*n* = 47). ɣH2AX intensity of *Sirt7*$^{-/-}$ samples was normalized to its WT control to remove technical bias. Two-tailed t-test. **c** Immunostaining of SYCP3 and SYCE1 in SYCP3-fully synapsed synaptonemal axes in 9-12-month-old WT and *Sirt7*$^{-/-}$ pachynemas showing no differences in SYCE1 deposition. Representative images from 3 biological replicates per genotype. Scale bar, 10 µm. **d** Immunostaining images of ɣH2AX and p53 in testicular sections of 9-12-month-old WT and *Sirt7*$^{-/-}$ mice. Representative images from 3 independent testis samples per genotype. ɣH2AX staining and nuclear morphology were used to identify spermatocytes. Blue arrows mark spermatocytes with nuclear p53 signal. Scale bar, 100 µm in full tubule images; 20 µm in zoom images. Source data are provided as a Source Data file.

SUPPLEMENTARY TABLES

Supplementary Table 1. *Sirt7* knockout CRISPR crRNA guide sequences

Gene	Guide	Exon	Sequence (5'->3')
Sirt7	1	1	/AltR1/rCrCrA rUrUrA rGrGrA rCrCrC rCrGrA rUrArA rUrCrG rUrUrU rUrArG rArGrC rUrArU rGrCrU /AltR2/
	2	1	/AltR1/rCrGrG rArGrC rGrCrA rArArG rCrUrG rCrUrG rArGrG rUrUrU rUrArG rArGrC rUrArU rGrCrU /AltR2/

Supplementary Table 2. List of antibodies for Western Blot

Primary antibodies	Host	Dilution	Source	Catalogue number	Lot #
H3K36ac (D9T5Q)	Rabbit	1:1000	Cell Signaling Technology	27683	1
H3K9me3	Rabbit	1:1000	Abcam LTD.	ab8898	GR285802-1
H3K18ac	Rabbit	1:1000	Abcam LTD.	ab1191	GR34489371-1
H3K36me2 (C75H12)	Rabbit	1:1000	Cell Signaling Technology	2901	5
H3K36me3 (D5A7)	Rabbit	1:1000	Cell Signaling Technology	4909S	7
H3K4me3	Rabbit	1:1000	Abcam LTD.	ab8580	GR3425199-1
H4K20me1	Rabbit	1:1000	Abcam LTD.	ab9051	
H4K20me3	Rabbit	1:1000	Abcam LTD.	ab9053	GR3429886-1
ɣH2AX	Rabbit	1:500	Abcam LTD.	ab2893	1079433-9
H3	Rabbit	1:10.000	Abcam LTD.	ab1791	GR3198176-1
H4 (L64C1)	Rabbit	1:1000	Cell Signaling Technology	2935	6
H2AX	Rabbit	1:1000	Abcam LTD.	ab11175	GR3263061-6
SIRT7 (C-3)	Mouse	1:1000	Santa Cruz Biotechnology	sc-365344	C0124
SYCP3 (D-1)	Mouse	1:1000	Santa Cruz Biotechnology	sc-74569	G1019
Flag	Rabbit	1:1000	Sigma-Aldrich	F7425	395474
α-Tubulin	Mouse	1:10000	Sigma-Aldrich	T6074	117M4846V
**Secondary antibodies**	**Host**	**Dilution**	**Source**	**Catalogue number**	**Lot #**
Anti-Mouse IgG-HRP	Rabbit	1:10.000	Sigma-Aldrich	A9044	291320
Anti-Rabbit IgG-HRP	Goat	1:10.000	Sigma-Aldrich	A0545	403410

Supplementary Table 3. List of antibodies for immunostaining

Primary antibodies	Host species	Dilution for chromosome spreads	Dilution for testicular sections	Source	Catalogue number	Lot #
SYCP3 (D-1)	Mouse	1:500	1:200	Santa Cruz Biotechnology	sc-74569	G1019
SYCP3	Rabbit	1:500	-	Novus Biologicals	NB300-232	D172625-4
SYCP3	Rabbit	1:200	-	Abcam	ab15093	GR3225426-1
ɣH2AX (JBW301)	Mouse	1:2000	1:1000	Sigma Aldrich	05-636-I	3153259
RAD51	Rabbit	1:250	-	Sigma Aldrich	PC-130	4238440
HORMAD1	Rabbit	1:200	-	Proteintech	13917-1-AP	78471
H3K36ac (D9T5Q)	Rabbit	1:1000	1:500	Cell Signaling Technology	27683	1
PLZF	Rabbit	-	1:1000	Abcam LTD.	ab189849	GR3445406-2
SOX9	Rabbit	-	1:200	Sigma Aldrich	AB5535	4266532
H1T	Guinea pig	1:500	-	Gift from M.A. Handel		
SYCE1	Rabbit	1:200	-	Proteintech	17406-1-AP	10274
MLH1 (G168-15)	Mouse	1:100	-	BD Pharmigen	550838	3124138
p53 (1C12)	Mouse	-	1:100	Cell Signaling	2524	11
**Secondary antibodies**	**Host species**	**Dilution for chromosome spreads**	**Dilution for testicular sections**	**Source**	**Catalogue number**	**Lot #**
Alexa Fluor-488 anti-rabbit IgG	Goat	1:500	1:500	Thermo Fisher	A-11034	2380031
Alexa Fluor-488 anti-guinea pig IgG	Goat	1:200	-	Thermo Fisher	A-11073	2892451
Alexa Fluor-555 anti-mouse IgG	Goat	1:500	1:500	Thermo Fisher	A-32727	UL287768
Alexa Fluor-568 anti-rabbit IgG	Donkey	1:200	-	Thermo Fisher	A-10042	2941306
Alexa Fluor-647 anti-rabbit IgG	Goat	1:500	1:500	Thermo Fisher	A-21245	1845042
Alexa Fluor-647 anti-rabbit IgG	Donkey	1:200	-	Thermo Fisher	A-31571	2098544

UNCROPPED BLOTS FOR SUPPLEMENTARY FIGURES

Uncropped blots for Supplementary Fig. 2a

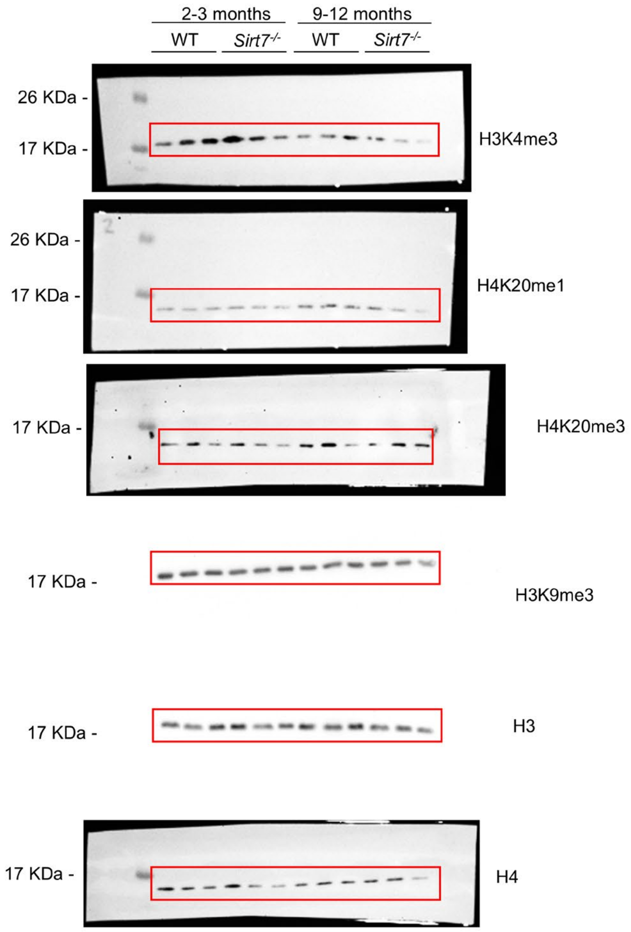

Uncropped blots for Supplementary Fig. 3e

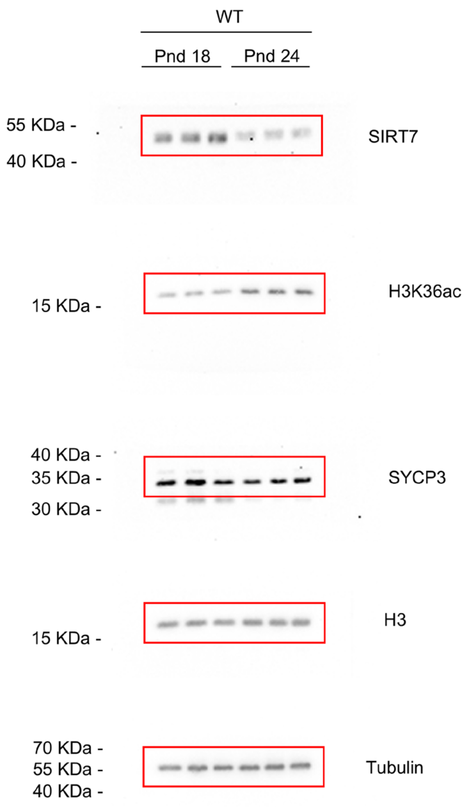

Uncropped blots for Supplementary Fig. 4

g

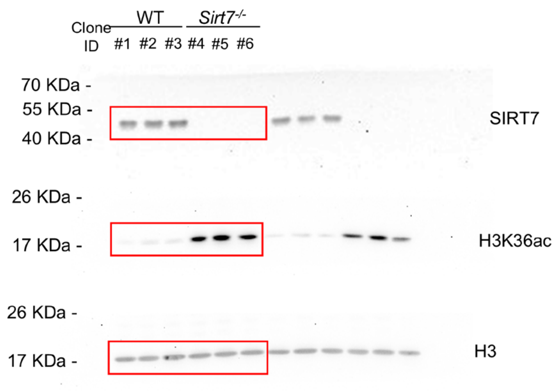

h

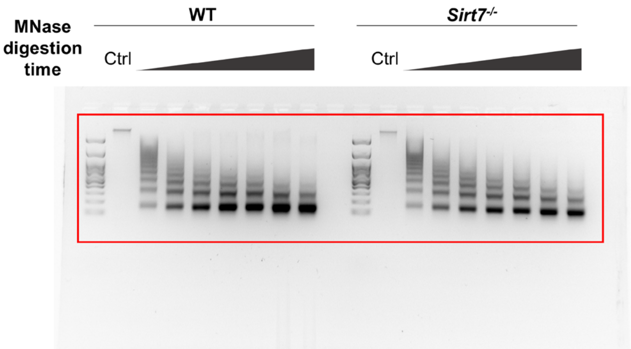

k

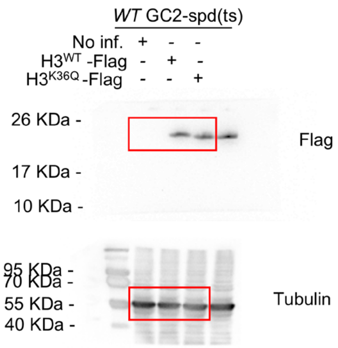

Uncropped blots for Supplementary Fig. 5a

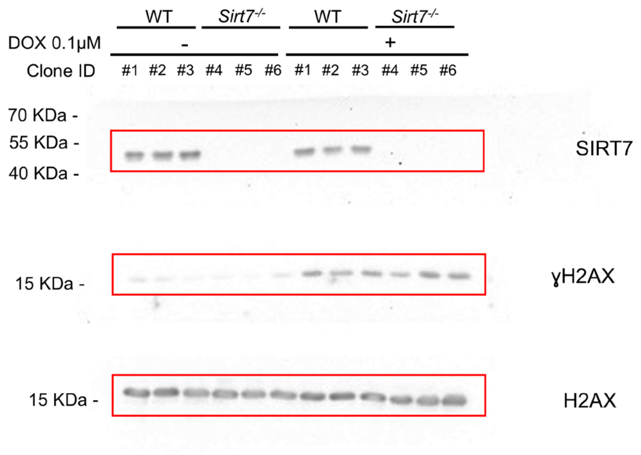

Supplement: Supplementary file 1 — Supplementary Information [file 41467_2026_72540_MOESM1_ESM.pdf]
