# Supplementary material for: SIRT7 links H3K36ac epigenetic regulation with genome maintenance in the mouse testis
Source: Nat Commun. 2026 Apr 28;17:5809. doi: 10.1038/s41467-026-72540-3 (PMC13328743; doi:10.1038/s41467-026-72540-3)
Supplement: Supplementary file 2 — Reporting Summary [file 41467_2026_72540_MOESM2_ESM.pdf]

nature portfolio

Corresponding author(s): Berta N Vazquez
Karen Schindler
Alejandro Vaquero

Last updated by author(s): Apr 7, 2026

Reporting Summary

Nature Portfolio wishes to improve the reproducibility of the work that we publish. This form provides structure for consistency and transparency in reporting. For further information on Nature Portfolio policies, see our Editorial Policies and the Editorial Policy Checklist.

Statistics

For all statistical analyses, confirm that the following items are present in the figure legend, table legend, main text, or Methods section.

n/a	Confirmed	
☐	☒	The exact sample size (n) for each experimental group/condition, given as a discrete number and unit of measurement
☐	☒	A statement on whether measurements were taken from distinct samples or whether the same sample was measured repeatedly
☐	☒	The statistical test(s) used AND whether they are one- or two-sided<br>*Only common tests should be described solely by name; describe more complex techniques in the Methods section.*
☐	☒	A description of all covariates tested
☐	☒	A description of any assumptions or corrections, such as tests of normality and adjustment for multiple comparisons
☐	☒	A full description of the statistical parameters including central tendency (e.g. means) or other basic estimates (e.g. regression coefficient) AND variation (e.g. standard deviation) or associated estimates of uncertainty (e.g. confidence intervals)
☐	☒	For null hypothesis testing, the test statistic (e.g. F, t, r) with confidence intervals, effect sizes, degrees of freedom and P value noted<br>*Give P values as exact values whenever suitable.*
☒	☐	For Bayesian analysis, information on the choice of priors and Markov chain Monte Carlo settings
☒	☐	For hierarchical and complex designs, identification of the appropriate level for tests and full reporting of outcomes
☐	☒	Estimates of effect sizes (e.g. Cohen's d, Pearson's r), indicating how they were calculated

Our web collection on statistics for biologists contains articles on many of the points above.

Software and code

Policy information about availability of computer code

Data collection	Flow cytometry samples were run in a FACS Canto II (BD Biosciences). Data were collected with BD FACS Diva software 6.1.3.<br>Western blot images were obtained with an iBright 1500 (Invitrogen)<br>Chromatin shearing for ChIP experiments was performed with a Covaris M220 system<br>Immunofluorescence images were acquired in a Stellaris 8 confocal microscope (Leica).<br>Histological images were acquired in a Olympus BX53 microscope equipped with an Olympus SC180 camera.<br>Comet images were captured on an Olympus BX51 fluorescence microscope equipped with an Olympus DP73 camera.<br>ChIP-Seq, ATAC-Seq and RNA-Seq data were acquired on a DNBSEQ-G400 instrument.

Data analysis	Data presentation and statistical analyses: GraphPad Prism 8.0.1.<br>Flow cytometry data analysis: FlowJo 7.6.<br>Western blot and agarose gels densitometric estimation, and immunofluorescence image analysis: Fiji 2.3.0 (Schindelin J et al. 2012. Nat Methods)<br>Brightfield image analysis: QuPath (version 0.5.1)<br>Comet analysis: CometScore software v.1.5 (TriTek Corp.)<br>Heatmap visualization and clustering: Morpheus software (Broad Institute).<br>Gene ontolgy terms: Enrichr tool.<br>Visualization of ChIP-Seq and RNA-Seq tracks: Integrated Genome Viewer 2.7.2.<br>Differential Expression Analysis: DESeq2

For manuscripts utilizing custom algorithms or software that are central to the research but not yet described in published literature, software must be made available to editors and reviewers. We strongly encourage code deposition in a community repository (e.g. GitHub). See the Nature Portfolio guidelines for submitting code & software for further information.

Data

Policy information about availability of data

All manuscripts must include a data availability statement. This statement should provide the following information, where applicable:

- Accession codes, unique identifiers, or web links for publicly available datasets
- A description of any restrictions on data availability
- For clinical datasets or third party data, please ensure that the statement adheres to our policy

The raw and processed ChIP–seq data generated in this study have been deposited in the Sequence Read Archive (SRA) and Gene Expression Omnibus (GEO) repositories under accession numbers SRP656109 and GSE314213, respectively. The ATAC–seq data have been deposited in SRA and GEO repositories under accession numbers SRP656712 and GSE314480, respectively. and the RNA–seq data have been deposited in SRA and GEO repositories under accession numbers SRP654471 and GSE313468, respectively.

Sirtuin expression levels in the various testis cell populations were obtained from publicly available scRNA-seq data from young adult humans (Guo, J. et al. 2018 cell res) and adult mice (Green, C. D. et al. 2018 Dev cell). Processed data files from adult mice were obtained from Gene Omnibus Expression (GEO) accession code GSE112393. Expression plots from human datasets were retrieved from the public online resource 'Human testis Atlas' (https://humantestisatlas.shinyapps.io/humantestisatlas1/) (Guo, J. et al. 2020 Cell Stem Cell).

Research involving human participants, their data, or biological material

Policy information about studies with human participants or human data. See also policy information about sex, gender (identity/presentation), and sexual orientation and race, ethnicity and racism.

Reporting on sex and gender	N/A
Reporting on race, ethnicity, or other socially relevant groupings	N/A
Population characteristics	N/A
Recruitment	N/A
Ethics oversight	N/A

Note that full information on the approval of the study protocol must also be provided in the manuscript.

Field-specific reporting

Please select the one below that is the best fit for your research. If you are not sure, read the appropriate sections before making your selection.

[x] Life sciences [ ] Behavioural & social sciences [ ] Ecological, evolutionary & environmental sciences

For a reference copy of the document with all sections, see nature.com/documents/nr-reporting-summary-flat.pdf

Life sciences study design

All studies must disclose on these points even when the disclosure is negative.

Sample size	No sample size calculation was performed, as experiments were performed on genetically identical mice or cell lines. Sample size was based on previous experiments, as well as on the 3R principles to reduce the number of animal used. Sample sizes were sufficient to detect differences between experimental groups.
Data exclusions	No data was excluded from the manuscript.
Replication	All replicates were performed under the same conditions and are described in the manuscript. Results were similarly replicated in at least two

	independent experiments, and results pooled from independent experiments are indicated in the corresponding figure legends.
Randomization	Experiments were performed with genetically identical mice, so randomization was not required.
Blinding	Investigators were not blinded, as most experiments were performed by a single person. In the case of sequencing, the bioinformatic analysis was performed by independent researchers that were not involved in the preparation of the samples.

Reporting for specific materials, systems and methods

We require information from authors about some types of materials, experimental systems and methods used in many studies. Here, indicate whether each material, system or method listed is relevant to your study. If you are not sure if a list item applies to your research, read the appropriate section before selecting a response.

Materials & experimental systems

n/a	Involved in the study
☐	☒ Antibodies
☐	☒ Eukaryotic cell lines
☒	☐ Palaeontology and archaeology
☐	☒ Animals and other organisms
☒	☐ Clinical data
☒	☐ Dual use research of concern
☒	☐ Plants

Methods

n/a	Involved in the study
☐	☒ ChIP-seq
☐	☒ Flow cytometry
☒	☐ MRI-based neuroimaging

Antibodies

Antibodies used

For Western blotting:
H3K36ac (D9T5Q)(1:1000 Cell Signaling Technology, 27683, lot:1)
H3K9me3 (1:1000, Abcam LTD., ab8898, lot: GR285802-1)
H3K18ac (1:1000, Abcam LTD., ab1191, lot: GR34489371-1)
H3K36me2 (C75H12)(1:1000, Cell Signaling Technology, 2901, lot: 5)
H3K36me3 (D5A7)(1:1000, Cell Signaling Technology, 4909S, lot: 7)
H3K4me3 (1:1000, Abcam LTD., ab8580, lot: GR3425199-1)
H4K20me1 (1:1000, Abcam LTD., ab9051)
H4K20me3 (5E10-D8)(1:1000, Novus Biologicals, NBP130091SS, lot: GR3429886-1)
ɣH2AX (1:500, Abcam LTD. , ab2893, lot: 1079433-9)
H3 (1:10.000, Abcam LTD. , ab1791, lot: GR3198176-1)
H4 (L64C1) (1:1000, Cell Signaling Technology, 2935, lot: 6)
H2AX (1:1000, Abcam LTD. , ab11175, lot: GR3263061-6)
SIRT7 (C-3) (1:1000, Santa Cruz Biotechnology, sc-365344, lot: C0124)
SYCP3 (D-1)(1:1000, Santa Cruz Biotechnology, sc-74569, lot: G1019)
Flag (1:1000, Sigma-Aldrich, F7425, lot: 395474)
alpha-tubulin (1:10000, Sigma-Aldrich, T6074, lot: 117M4846V)
Anti-Mouse IgG-HRP (1:10.000, Sigma-Aldrich, A9044, lot: 291320)
Anti-Rabbit IgG- HRP (1:10.000, Sigma-Aldrich, A0545, lot: 403410)

For immunostaining:
SYCP3 (D-1)(1:500, Santa Cruz Biotechnology, sc-74569, lot: G1019)
SYCP3 (1:500, Novus Biologicals, NB300-232, lot: D172625-4)
SYCP3 (1:200, Abcam, ab15093, lot: GR3225426-1)
ɣH2AX (JBW301) (1:2000, Sigma Aldrich, 05-636-I, lot: 3153259)
RAD51 (1:250, Sigma Aldrich, PC-130, lot: 4238440)
HORMAD1 (1:200, Proteintech, 13917-1-AP, lot: 78471)
H3K36ac (D9T5Q)(1:1000, Cell Signaling Technology, 27683, lot: 1)
PLZF (1:1000, Abcam LTD., ab189849, lot: GR3445406-2)
SOX9 (1:200, Sigma Aldrich, AB5535, lot: 4266532)
H1T (1:500, Gift from M.A. Handel)
SYCE1 (1:200, Proteintech, 17406-1-AP, lot: 10274)
MLH1 (G168-15) (1:100, BD,Pharmigen, 550838, lot: 3124138)
p53 (1C12) (1:100, Cell Signaling, 2524, lot: 11)
Alexa Fluor-488 anti-rabbit IgG (1:500, Thermo Fisher, A-11034, lot: 2380031)
Alexa Fluor-488 anti-guinea pig IgG (1:200, Thermo Fisher, A-11073, lot: 2892451)
Alexa Fluor-555 anti-mouse IgG (1:500, Thermo Fisher, A-32727, lot: UL287768)
Alexa Fluor-568 anti-rabbit IgG (1:200, Thermo Fisher, A-10042, lot: 2941306)
Alexa Fluor-647 anti-rabbit IgG (1:500, Thermo Fisher, A-21245, lot: 1845042)
Alexa Fluor-647 anti-rabbit IgG (1:200, Thermo Fisher, A-31571, lot: 2098544)

	For Flow Cytometry: PLZF (1:1400, Abcam LTD., ab189849, lot: GR3445406-2)<br><br>For ChIP: H3K36ac (D9T5Q)(Cell Signaling Technology, 27683)
Validation	All the antibodies used are commercially available and most of them were validated by the manufacturer for the intended applications and species (validation information available at vendor's website). Exceptions include: anti-H1T antibody, which was produced in M.A. Handel lab where they validated it. Its use for immunofluorescence is supported by many publications of the meiosis field. Anti-MLH1 antibody (550838) use for IF is not validated by manufacturer but its reactivity against mouse protein is supported by multiple publications as also stated in the vendor's website. The anti-PLZF antibody (ab189849) has not been reported for flow cytometry or IF in the vendor's website, but is a well-established antibody used for immunofluorescence in the publications of the field and in our hands shows clear specificity for PLZF+ cells in testicular sections. We further validated its specificity for flow cytometry using a Hoechst costaining in a pool of testicular cells.

Eukaryotic cell lines

Policy information about cell lines and Sex and Gender in Research

Cell line source(s)	The mouse spermatocyte cell line GC-2spd(ts) was purchased from ATCC (CRL-2196). Platinum A cells were purchased from Cell Biolabs (RV-102).
Authentication	The cell lines were not authenticated
Mycoplasma contamination	Cells were not tested for mycoplasma contamination
Commonly misidentified lines<br>(See ICLAC register)	None of the cell lines used were found in the Commonly misidentified lines database

Animals and other research organisms

Policy information about studies involving animals; ARRIVE guidelines recommended for reporting animal research, and Sex and Gender in Research

Laboratory animals	Wild-type and Sirt7-/- mice were on a 129S1/Sv background. Mice from three different age groups (2-3, 4-6, 9-12 month-old) were used for aging studies. Mice were housed under controlled conditions with a temperature maintained at 21–25°C, relative humidity of 40–70%, and a 12h light/dark cycle, including a 15min gradual light intensity ramp to simulate sunrise and sunset.
Wild animals	Study did not involve wild animals.
Reporting on sex	This work is focused on the study of spermatogenic cells. Therefore, only male mice were used in the study. Female mice were used for breeding studies, but they were not analyzed.
Field-collected samples	The study did not involve field-collected samples.
Ethics oversight	Animals were bred in the animal facilities of the Comparative Medicine and Bioimage Centre (CMCiB) of the Gemans Trias i Pujol Research Institute (IGTP) and Rutgers University, respectively. All animal procedures were approved by the Animal Care and Ethics Committee of the institutions where they were carried out. All animal experiments performed in this study were approved by the Rutgers IACUC (protocol #201702497) and the Catalan Government (protocol #10472).

Note that full information on the approval of the study protocol must also be provided in the manuscript.

Plants

Seed stocks	N/A
Novel plant genotypes	N/A
Authentication	N/A

ChIP-seq

Data deposition

- [x] Confirm that both raw and final processed data have been deposited in a public database such as GEO.
- [x] Confirm that you have deposited or provided access to graph files (e.g. BED files) for the called peaks.

Data access links<br>*May remain private before publication.*	The ChIP-seq raw and processed data generated in this study has been deposited in the GEO under the accession number GSE314213 and in the SRA under the accession number SRP656109.
Files in database submission	ChIP_S7KO2_L1.bw<br>ChIP_S7KO3_L1.bw<br>ChIP_S7KO4_L1.bw<br>ChIP_WT2_L1.bw<br>ChIP_WT3_L1.bw<br>ChIP_WT4_L1.bw<br>Input_S7KO2_L1.bw<br>Input_S7KO3_L1.bw<br>Input_S7KO4_L1.bw<br>Input_WT2_L1.bw<br>Input_WT3_L1.bw<br>Input_WT4_L1.bw<br>S7KO2_H3K36ac.broadPeak<br>S7KO3_H3K36ac.broadPeak<br>S7KO4_H3K36ac.broadPeak<br>WT2_H3K36ac.broadPeak<br>WT3_H3K36ac.broadPeak<br>WT4_H3K36ac.broadPeak
Genome browser session<br>(e.g. UCSC)	No longer applicable

Methodology

Replicates	Three biological replicates for each sample. Each replicate represents spermatogonial cells obtained from a different mouse.
Sequencing depth	Clean reads per sample, after removing adapters with Trim Galore (PE100 sequencing):<br><br>Input_WT2: 27391107<br>Input_WT3: 31485376<br>Input_WT4: 25602471<br>Input_S7KO2: 29840107<br>Input_S7KO3: 32524505<br>Input_S7KO4: 29450886<br>ChIP_WT2: 33709038<br>ChIP_WT3: 30713963<br>ChIP_WT4: 19618573<br>ChIP_S7KO2: 32310762<br>ChIP_S7KO3: 21605595<br>ChIP_S7KO4: 31006252
Antibodies	Cell signaling D9T5Q (Cat #27683). 1 µL (230 ng/µL) per sample.
Peak calling parameters	Reads were mapped with Bowtie2. Non-duplicated & uniquely mapped reads were filtered with SAMtools (samtools view -bS -q 30) and Sambamba (sambamba view -F "[XS] == null and not unmapped and not duplicate). Reads were sorted and indexed with sambamba. Peaks were called with MACS2 (macs2 callpeak -f BAMPE --broad --broad-cutoff 0.1 -g mm), taking into account both ChIP (-t) and input control (-i) for each samples
Data quality	FastQC was employed to check data quality of raw fastq files. Adapters were removed with Trim Galore. Aligned reads were filter to remove low quality reads (MAPQ > 30) and keep only uniquely mapped and non-duplicated reads.
Software	FastQC 0.12.1<br>Trim Galore 0.6.6<br>Bowtie2 2.4.4.1<br>SAMtools 1.19.2<br>Sambamba 0.8.2<br>MACS2 2.2.5<br>ChIPseeker 1.42.1<br>bedtools 2.27.1

bwtool 1.0
DiffBind 3.16

Flow Cytometry

Plots

Confirm that:

- [x] The axis labels state the marker and fluorochrome used (e.g. CD4-FITC).
- [x] The axis scales are clearly visible. Include numbers along axes only for bottom left plot of group (a 'group' is an analysis of identical markers).
- [ ] All plots are contour plots with outliers or pseudocolor plots.
- [ ] A numerical value for number of cells or percentage (with statistics) is provided.

Methodology

Sample preparation	50000 cells were washed once with ice-cold staining buffer (4% FBS, 2 mM EDTA in PBS) and incubated for 10 min with 3% FBS PBS blocking buffer in ice. Cell fixation and permeabilization were performed with FoxP3 fixation/permeabilization buffer (eBioscience, 00-5523-00) following the manufacturer's instructions. Briefly, cells were fixed for 30 min at room temperature and washed once in permeabilization buffer. Cells were blocked by supplementing permeabilization buffer with 1% FBS and stained with anti-PLZF antibody (Abcam, ab189849 1:1400). Cells were washed once and further incubated with an anti-IgG (H+L) secondary antibody (Invitrogen, 1:1200)
Instrument	FACSymphony A1 cytometer (BD Biosciences)
Software	Data were collected with BD FACS Diva software 6.1.3. and analyzed with FlowJo 7.6. sofware.
Cell population abundance	N/A
Gating strategy	In all samples, singlets were gated first by FSC-H and FSC-A

- [x] Tick this box to confirm that a figure exemplifying the gating strategy is provided in the Supplementary Information.
