# Supplementary material for: SIRT7 links H3K36ac epigenetic regulation with genome maintenance in the mouse testis
Source: Nat Commun. 2026 Apr 28;17:5809. doi: 10.1038/s41467-026-72540-3 (PMC13328743; doi:10.1038/s41467-026-72540-3)
Supplement: Supplementary file 3 — Transparent Peer Review File [file 41467_2026_72540_MOESM3_ESM.pdf]

nature portfolio

Peer Review File

SIRT7 links H3K36ac epigenetic regulation with genome maintenance in the mouse testis

Corresponding Author: Dr Berta Vazquez

This file contains all reviewer reports in order by version, followed by all author rebuttals in order by version. Parts of this Peer Review File have been redacted as indicated to maintain the confidentiality of unpublished data.

Version 0:

Reviewer comments:

Reviewer #1

(Remarks to the Author)
In the present study, the authors conducted a systematic evaluation of the impact of Sirt7 gene knockout on spermatogenesis in mice. The results revealed that Sirt7-/- male mice exhibited an infertility phenotype associated with accelerated physiological aging. These mice exhibited a significant decrease in reproductive capacity, characterized by reduced litter size and decreased testicular size, alongside increased pathological changes in the seminiferous tubules and abnormalities in spermatogenesis. Consistent with the observed infertility, the Sirt7-/- mice exhibited an increased incidence of pathological alterations in the seminiferous tubules, along with histologically evident disruptions in the normal progression of spermatogenesis. To further characterize the molecular basis of these observations, the authors performed comprehensive transcriptomic profiling of the testes. The transcriptional landscape of the Sirt7-/- testes exhibited striking similarities to that of aged wild-type mice, indicative of premature testicular aging in the knockout mice. Of particular note, the researchers identified persistent aberrations in the histone modification H3K36ac (H3K36 acetylation) in both spermatogonia and spermatocytes of the Sirt7-/- mice. These findings collectively suggest that Sirt7 plays a critical role in maintaining the integrity and functionality of the spermatogenesis process. This study provides novel insights into the mechanisms underlying Sirt7's function in spermatogenesis and opens new avenues for understanding the molecular basis of male fertility and age-related reproductive decline.
In the peer-review process, I identified the following major issues in the manuscript:
1. Imprecise and Incoherent Writing: The manuscript suffers from issues of unclear expression and lack of coherence, which hinder the reader's ability to grasp the authors' intentions and create reading obstacles.
2. Fragmented Experimental Results: The presentation of experimental results is disjointed, lacking logical flow and cohesion. This results in a fragmented narrative, making it difficult to follow the progression of the study.
3. Lack of Experimental Rigor: The experimental design and interpretation are overly speculative and insufficiently supported by rigorous logical reasoning and empirical evidence. Critical details, such as experimental conditions, sample sizes, and statistical methods, are inadequately described, undermining the reproducibility and robustness of the study.
4. Insufficient Mechanistic Analysis: The discussion of underlying mechanisms is inadequate, leaving the observed experimental phenomena poorly explained. This weakens the study's persuasiveness and scientific rigor.
Based on these issues, the manuscript does not currently meet the acceptance criteria for this journal. To improve the manuscript for further review, the authors are strongly advised to address the following points:
1. Enhance Writing Quality: Improve the clarity and logical structure of the text to enhance readability and ensure that the scientific rationale is effectively communicated.
2. Refine Experimental Details: Provide detailed and precise descriptions of experimental conditions, sample sizes, and statistical methodologies to ensure the reproducibility of the findings and strengthen the validity of the study.
3. Deepen Mechanistic Insights: Conduct a more thorough investigation of the mechanisms underlying the observed experimental phenomena, with a focus on functional experiments and mechanistic analysis, to improve the study's scientific persuasiveness.
Only after substantial improvements in these areas will the manuscript be deemed suitable for further evaluation.
In the peer-review process, I found that the manuscript still has some room for improvement in terms of experimental design and data analysis, particularly in the aspects of confirmatory experiments and mechanism exploration. Based on the current study's shortcomings, it is recommended that the authors supplement the following experiments.
1. A systematic comparison of in vitro and in vivo models: Based on the in vitro culture of GC2 cells, design in vivo experiments to validate the observed changes in H3K36ac and their effects on chromatin accessibility and DNA damage repair, ensuring consistency in results between in vitro and in vivo settings.

2. Analysis of downstream target genes of H3K36ac: Employ techniques such as ChIP-seq to identify the target genes associated with H3K36ac-enriched regions, followed by functional experiments to validate the roles of these genes in spermatogenesis and reproductive aging in male germ cells. Additionally, investigate the direct connection between H3K36ac and the Sirt7 gene.
Specific suggestions and questions are listed below:
1. Revise the entire manuscript to enhance language clarity and precision, ensuring that all expressions are unambiguous and aligned with academic standards. Review the terminology to confirm its appropriateness and consistency with established academic norms. Optimize sentence structure to improve logical coherence and readability, ensuring seamless transitions between paragraphs to facilitate readers' understanding of the research logic.
(a) The issue of tense consistency.
In the Introduction section, the tense usage in the following sentence is inconsistent:
Original sentence: "This lack of attention is due to the long-held view that aging exerted a minimal impact on male fertility."
Issue: The previous sentence uses the present tense ("the impact of aging on male germ cell development have not received comparable attention"), while the following sentence uses the past tense ("exerted"), which is inconsistent with the context.
Suggestion: Change "exerted" to "exerts" to maintain tense consistency and better align with the common way of expressing ongoing phenomena in scientific fields.
(b) Improper use of articles and terminology.
Original sentence: "During meiosis, spermatocytes complete a series of unique events, including double-strand break (DSB) formation, recruitment of DNA damage response (DDR) proteins, homology search, chromosome pairing, and, in some cases, resolution of DSB as crossover events."
Issue: The definite article "the" is missing, and "DSB" should be in the plural form "DSBs."
Suggestion: Change to "the resolution of DSBs as crossover events" to ensure grammatical accuracy and proper terminology.
2. The manuscript mentions that "aging exerted a minimal impact on male fertility.," but this viewpoint lacks sufficient scientific evidence or citation support.
Issue: Although the decline in male reproductive ability is slower, male fertility is indeed significantly affected with age. Stating that "aging has a minimal impact on male fertility" may not be entirely accurate and lacks supporting references.
Suggestion: Please provide relevant scientific evidence or cite authoritative literature to support this claim. Alternatively, you could rephrase it, such as stating "compared to women, male fertility is less affected by age, but it is not unaffected," and cite related references.
3. Inquiries regarding experimental design and data analysis:
(a) The bar chart of "litter size" in Figure 1b remains unclear regarding the specific data represented by each circle. The authors are requested to provide detailed explanations, particularly clarifying whether the circles correspond to the litter-specific data shown in Figure 1c (L1-L7). Notably, the WT x WT group in Figure 1b shows 4 points, while the WT x Sirt7-/- group shows 6 points, which appears inconsistent with the data presented in Figure 1c. The authors are asked to explicitly explain the reasons for the sample size discrepancy between these two groups, whether it results from experimental design differences, data collection omissions, or other potential factors that may influence data presentation. Additionally, it is crucial to ensure that the annotation method of data points aligns with the experimental design logic. Therefore, the authors are requested to thoroughly review whether Figure 1b and Figure 1c present data with logical inconsistencies. It is strongly recommended to clearly explain the relationship between the two figures and ensure the accuracy and consistency of the data to avoid potential misunderstandings by readers due to the apparent disparity in the number of points between the WT x WT and WT x Sirt7-/- groups in Figure 1b.
(b) Issues with Figure 1f: Sample Size and Replicates. In Figure 1f, the number of samples and replicates for each group and time point is not clearly specified. To ensure the scientific rigor and reliability of the experiment, it is strongly recommended that the authors explicitly state the sample sizes for each group and time point. For example, if the WT group used 9 testes while the Sirt7-/- group only used 3 testes, this discrepancy in sample size may impact the accuracy of the experimental results. It is essential for the authors to explain the reasons behind this sample size imbalance and assess whether it could introduce potential biases in the statistical analysis outcomes. Additionally, based on the statistical results in Figure 1f, 33.33% of mice in the 9-12 months Sirt7-/- group were categorized as "Mildly affected," whereas 40% of mice in the 4-6 months Sirt7-/- group fell into the same category. However, the morphological results presented in the corresponding supplementary figure do not fully align with these statistical findings. The authors are requested to provide a detailed explanation for this observed inconsistency.
(c) Issues with Figure 1g: Statistical Details and Sample Size Discrepancy. In Figure 1g, the specific types of reproductive cells (e.g., spermatogonia, spermatocytes, sperm, etc.) being counted and the specific details of the statistical methods are not clearly stated by the authors. Additionally, there remains an inconsistency in the sample sizes between the WT group and the Sirt7-/- group. It is strongly recommended that the authors provide a detailed explanation of the statistical methods used and clarify the potential reasons for the observed sample size discrepancy.
(d) In Figure 1i, it is unclear whether the statistical analysis refers to the number of bright spots marked by white arrows in Figure 1h. Additionally, the authors are requested to clarify the criteria used to determine whether a sperm has "high DNA damage" and provide detailed information about the statistical approach applied in this analysis.
4. The basis for selecting the time points for transcriptome sequencing.
In Figure 2, the authors performed transcriptome sequencing on testes from WT and Sirt7-/- mice, selecting the 2–3 months and 4–6 months points. However, based on the results shown in Figure 1, no significant differences were observed between the WT and Sirt7-/- groups at the 2–3 months time point, whereas significant differences were evident at the 4–6 month and 9–12 months time points. Therefore, we request the authors to explain why these earlier time points (2–3 months and 4–6 months) were selected for transcriptome sequencing instead of the later time points (4–6 months and 9–12 months), which might better reflect the early features of reproductive aging in Sirt7-/- mice. Additionally, in later experiments, the authors analyzed acetylation levels at the 2–3 months and 9–12 months points. We kindly ask the authors to provide the rationale for

selecting these specific time points and to clarify whether they considered age-related changes in testicular function when making these selections.
5. The contradiction between the number of differentially expressed genes in transcriptome analysis and the morphological phenotype differences.
In Figure 2b, the authors performed GSEA (Gene Set Enrichment Analysis) on the upregulated and downregulated genes between the WT and Sirt7-/- groups at the 2-3 months time point. However, no GSEA analysis was conducted on the differentially expressed genes in the 4-6 months group. Furthermore, the supplementary figures indicate that the number of differentially expressed genes in the 4-6 months group is fewer than in the 2-3 months group, despite the fact that the histomorphological phenotypic differences are more pronounced in the 4-6 months group. The authors are requested to provide a detailed explanation for why GSEA analysis was not performed on the 4-6 months group and to clarify how they interpret the phenomenon of decreasing numbers of differentially expressed genes with advancing age, particularly in light of the fact that knockout mice typically exhibit more pronounced phenotypes after reaching adulthood.
6. Discrepancy between acetylation levels and transcriptomic analysis results.
In the manuscript, it is suggested that Sirt7 knockout leads to increased acetylation levels, yet in the transcriptomic analysis, no significant enrichment of pathways related to acetylation was observed. The authors are requested to explain this discrepancy. Furthermore, in the transcriptomic analysis, the chromatin-associated factors in the testes of Sirt7-/- mice at the 2-3 months time point were primarily associated with methylation rather than acetylation, despite the subsequent WB (Western Blot) experiments showing no significant changes in methylation levels. The authors are kindly asked to provide a detailed explanation for this inconsistency and to explore potential underlying mechanisms, as well as to consider whether alternative regulatory mechanisms might be at play.
7. Impact of Sirt7 knockout on somatic cells.
In the transcriptomic analysis, the Leydig Cell Dysfunction pathway showed significant enrichment. The authors are kindly requested to address whether they considered the potential impact of Sirt7 knockout on testicular somatic cells, such as Leydig cells, and to explore whether the changes in these somatic cells might indirectly affect spermatogenesis.
8. Mechanism of Sirt7 in Spermatogonia and Spermatocyte Function.
The authors observed that Sirt7 exhibits high expression levels in spermatogonia and spermatocytes and identified its critical role in maintaining the population of undifferentiated spermatogonia cells during aging. Notwithstanding these observations, the study does not provide a detailed elucidation of the specific molecular mechanisms by which the Sirt7 gene maintains the population of undifferentiated spermatogonia. Nevertheless, the study fails to provide a detailed explanation of the specific molecular mechanisms of the Sirt7 gene in maintaining the population of undifferentiated spermatogonia, nor does it reveal its potential role in the events of spermatocyte meiosis during prophase.

Reviewer #2

(Remarks to the Author)
In this manuscript, Guitart-Solanes et al. investigated effects of depletion of Sirtuin 7 (SIRT7) gene in spermatogenesis. First, they found that loss of SIRT7 causes premature male reproductive defects and results in transcriptomic changes resembling premature aging. Furthermore, SIRT7 regulates H3K36ac in spermatogonia and spermatocytes. Lastly, they showed SIRT7 absence results in DNA damage accumulation in undifferentiated spermatogonia and impairs meiotic progression in an age-dependent manner. They concluded that SIRT7 links H3K36ac epigenetic regulation with genome maintenance in the aging mouse testis.
The study revealed the function of SIRT7 in the spermatogenesis. However, the conclusion concerning the age-dependent function is not necessarily supported by their data. The following points should be addressed.

Major points:
1. The author declared that SIRT7 links H3K36ac epigenetic regulation with genome maintenance in the aging mouse testis, however, I cannot see significant difference in the expression of H3K36ac levels with age in the figure 3A.
2. The author found that H3K36ac expression increased in pachynema and to a higher extent in diplonema in WT spermatocytes. Thus, GC-2 cell line was not appropriate in this study. Because The GC-2 cell line may be arrested in a premeiotic stage of differentiation, it was not recommended in the current study.
3. The author should check the expression of SIRT7 in the testicular sections using IF staining but not the analysis based on scRNA-seq.
4. The author declared that 9-12-month-old Sirt7-/- testes showed defective DNA damage repair and impaired meiotic progression. Does this deficiency could activate of DNA damage checkpoint, which could result in meiotic arrest ?
5. Line 234 I cannot see any significant accumulation of γH2AX in undifferentiated spermatogonia with age in the figure 6c first and third panel.
6. In the figure 7A, is there almost 10% of pachytene-like spermatocytes in the WT testis with 2-3 months old? The percentage was too high in the WT testis with 2-3 months old.
7. The author should check the CO formation and synapse in the meiosis using MLH1 and SYCE1 or SIX6OS1 antibodies.
8. The author did not decipher the mechanism of high sperm DNA fragmentation in the sperm of SIRT7 -/- epididymis. The DNA damage accumulation in undifferentiated spermatogonia or defective DNA damage repair in the meiosis? For the expression of SIRT7 was very low in the spermatids.

Minor points:
Line 160: western blot should be Western blot
Line 181 “in” should be in “Times New Roman” format
Line 225-236 the paragraph is not justified

Version 1:

Reviewer comments:

Reviewer #1

(Remarks to the Author)
1.There are still issues with unclear descriptions throughout the manuscript, which require a thorough review and revision. For example, in Figure 6C, what criteria are used to distinguish between early and middle pachytene stages? Based solely on the authors’ presented results, I see no morphological distinction between the two stages. Moreover, the early pachytene stage shown is not typical. I suggest the authors refer to more literature to accurately define the morphology of early pachytene and clarify the criteria used to differentiate it from the mid-to-late stages. Additionally, the authors should explain why the γH2AX signal on the XY chromosomes in Figure 6C appears diffuse rather than punctate. Is this an abnormal phenotype or due to other reasons? Please annotate this clearly.
2.The figure layout lacks rigor. For instance, the pachytene cell shown in Figure 6E is not representative and has poor morphology. In addition, multiple figures throughout the manuscript are unclear. Regarding Figure 4E, the authors did not clearly state which specific cell type the H3K36ac fluorescence intensity was measured from. This needs further clarification.
3.If the authors claim that knockout of this gene leads to increased H3K36ac levels and premature testicular degeneration, then why is the H3K36ac level in 9–12-month-old mice lower than in 2–3-month-old mice? According to the authors’ theory, the H3K36ac level in 9–12-month-old knockout mice should be higher than in younger mice, but the current results show the opposite. Please provide an explanation. Furthermore, there is no significant difference in H3K36ac levels between 9–12-month-old and 2–3-month-old wild-type mice, yet the authors claim that SIRT7-regulated H3K36ac is age-related. Considering that testicular tissue contains both somatic and germ cells, the authors are advised to replace the testis-based Western blot experiment with one performed on spermatogonia specifically. This may yield more accurate results. In addition, please provide the original Western blot images used, indicate the molecular weight of the target proteins, and explain how the quantification was performed for proteins of similar size. Lastly, in Figure 3A, the authors refer to the expression of SYCP3 protein. However, SYCP3 is commonly known to appear as two bands, while the authors only show one.
4.In the authors' response, it is stated that Sirt7 deficiency leads to impaired chromatin accessibility and increased accumulation of DSBs, thereby affecting spermatogonial homeostasis. The authors should explain the evidence supporting a link between the two them, according to existing results, no direct association between the two has been demonstrated
5.How were the concentration of DOI and the 16-hour treatment duration determined for the vivo testis culture experiments?
6. During months 9–12, KO mice exhibited a twofold increase in early pachytene stages and a reduction in middle and late stages relative to WT mice; however, why did the two groups show comparable diplotene stage counts?
7.The study's investigation of late spermatocytes is insufficient and does not align well with earlier findings.
8.The results seem more focused on showing that gene knockout–induced reproductive damage increases with age, rather than demonstrating a direct link to naturally aged mice.
9.Evaluating somatic cell numbers alone is not sufficient to conclude that the knockout has no effect on somatic cells.

Reviewer #2

(Remarks to the Author)
The authors have mostly addressed my questions. However, several points should be addressed before publication.
1. Figure 1d: It was weird that the testicular weight in 9-12 month was heavier than that in 2-3 month.
2. Figure 1e: right panel: “9-1-month-old”
3. Figure 3a: Please repeat the WB assay. For one thing, considerable within-group variability was observed. For another, the band (SIRT7) exhibits a hollow appearance. Furthermore, the expression of SIRT7 decreased sharply in Pnd 24 and Pnd 35. Please explain and add this part in discussion.
4. For the question “The author declared that 9-12-month-old Sirt7-/- testes showed defective DNA damage repair and impaired meiotic progression. Does this deficiency could activate of DNA damage checkpoint, which could result in meiotic arrest?”. Staining of CHK1/2 and P53 should be added to evaluate the activation of DNA damage checkpoint.

Version 2:

Reviewer comments:

Reviewer #1

(Remarks to the Author)
The authors have essentially addressed my concerns, and I suggest it to be published in Nature Communications.

Reviewer #2

(Remarks to the Author)
The authors have well addressed my comments.

Open Access This Peer Review File is licensed under a Creative Commons Attribution 4.0 International License, which permits use, sharing, adaptation, distribution and reproduction in any medium or format, as long as you give appropriate credit to the original author(s) and the source, provide a link to the Creative Commons license, and indicate if changes were made.
In cases where reviewers are anonymous, credit should be given to 'Anonymous Referee' and the source.
The images or other third party material in this Peer Review File are included in the article's Creative Commons license, unless indicated otherwise in a credit line to the material. If material is not included in the article's Creative Commons license and your intended use is not permitted by statutory regulation or exceeds the permitted use, you will need to obtain permission directly from the copyright holder.
To view a copy of this license, visit https://creativecommons.org/licenses/by/4.0/

We thank the reviewers for their constructive and insightful comments. We have carefully addressed all concerns and included substantial revisions that, in our view, significantly improve the clarity, coherence, and overall quality of the manuscript. The most significant changes include the addition of new ChIP-seq, RNA-seq, and ATAC-seq datasets generated from enriched spermatogonial cell populations; analyses of DNA damage and chromatin compaction in enriched spermatogonial cells; further characterization of meiotic prophase progression; and the inclusion of additional data points in Figure 1. All newly added or revised content is highlighted in yellow in the revised manuscript. We hope that the revised version will now be suitable for consideration for publication in *Nature Communications*.

Below are our point-by-point responses to reviewers. The reviewer's comments are in blue font and our responses are in black font.

Reviewer 1

In the present study, the authors conducted a systematic evaluation of the impact of Sirt7 gene knockout on spermatogenesis in mice. The results revealed that Sirt7-/- male mice exhibited an infertility phenotype associated with accelerated physiological aging. These mice exhibited a significant decrease in reproductive capacity, characterized by reduced litter size and decreased testicular size, alongside increased pathological changes in the seminiferous tubules and abnormalities in spermatogenesis. Consistent with the observed infertility, the Sirt7-/- mice exhibited an increased incidence of pathological alterations in the seminiferous tubules, along with histologically evident disruptions in the normal progression of spermatogenesis. To further characterize the molecular basis of these observations, the authors performed comprehensive transcriptomic profiling of the testes. The transcriptional landscape of the Sirt7-/- testes exhibited striking similarities to that of aged wild-type mice, indicative of premature testicular aging in the knockout mice. On note, the researchers identified persistent aberrations in the histone modification H3K36ac (H3K36 acetylation) in both spermatogonia and spermatocytes of the Sirt7-/- mice. These findings collectively suggest that Sirt7 plays a critical role in maintaining the integrity and functionality of the spermatogenesis process. This study provides novel insights into the mechanisms underlying Sirt7's function in spermatogenesis and opens new avenues for understanding the molecular basis of male fertility and age-related reproductive decline. In the peer-review process, I identified the following major issues in the manuscript:

1. Imprecise and Incoherent Writing: The manuscript suffers from issues of unclear expression and lack of coherence, which hinder the reader's ability to grasp the authors' intentions and create reading obstacles.

We thank the reviewer for highlighting this issue. We have thoroughly revised the manuscript to ensure clarity and coherence throughout. Furthermore, the manuscript has been professionally edited by an experienced English language editor.

2. Fragmented Experimental Results: The presentation of experimental results is disjointed, lacking logical flow and cohesion. This results in a fragmented narrative, making it difficult to follow the progression of the study.

We thank the reviewer for this valuable comment. In the revised manuscript, we have substantially reorganized the Results section to improve the logical flow and overall cohesion. The study now begins with the phenotypic characterization of *Sirt7*$^{-/-}$ males and testes (Fig. 1 and Extended Data

Fig. 1), the identification of H3K36ac deregulation in *Sirt7*$^{-/-}$ testes (Fig. 2 and Extended Data Fig. 2) and characterization of H3K36ac dynamics during spermatogenesis and aging (Fig. 3 and Extended Data Fig. 3). Subsequently, we focus on undifferentiated spermatogonia, presenting the genome-wide mapping of H3K36ac (Fig. 4 and Extended Data Fig. 4), chromatin accessibility analyses (Fig. 4 and Extended Data Fig. 4) and the examination of the DNA damage response (Fig. 5 and Extended Data Fig. 5). Finally, we assess the consequences of SIRT7 deficiency in undifferentiated spermatogonia (Fig. 5) and meiotic progression in aging testes (Fig. 6 and Extended Data Fig. 6). In addition, we have removed tangential results, including those previously shown in Figure 2 referring to whole testis RNA-sequencing, that contributed to a fragmented narrative. The revised manuscript now provides a clear and integrated narrative of how SIRT7 contributes to spermatogonia homeostasis and meiotic progression during aging.

3. Lack of Experimental Rigor: The experimental design and interpretation are overly speculative and insufficiently supported by rigorous logical reasoning and empirical evidence. Critical details, such as experimental conditions, sample sizes, and statistical methods, are inadequately described, undermining the reproducibility and robustness of the study.

We thank the reviewer for highlighting this important point. In response, we have refined and expanded the descriptions of all experimental conditions and statistical analyses in the Material and Methods and Results sections, and Figure legends to ensure full transparency and reproducibility. Additionally, we have increased sample sizes for some analyses to improve the robustness of the study.

4. Insufficient Mechanistic Analysis: The discussion of underlying mechanisms is inadequate, leaving the observed experimental phenomena poorly explained. This weakens the study's persuasiveness and scientific rigor.

We thank the reviewer for raising this important concern. In the revised manuscript, we have expanded our mechanistic analysis and incorporated new experimental data that provide key insights into how SIRT7 regulates spermatogonia homeostasis and meiotic progression. Specifically, we now include the genome-wide H3K36ac profiling, transcriptomics, chromatin accessibility analysis (Fig. 4 and Extended Data Fig. 4), and stress-induced DNA damage assays (Fig. 5) all conducted in *Wt* and *Sirt7*$^{-/-}$ primary spermatogonia, which together support a coherent mechanistic model for SIRT7 function. Our results support a critical role for SIRT7 in maintaining genome stability during male gametogenesis and aging. These new results substantially strengthen the mechanistic depth and scientific rigor of the study.

Based on these issues, the manuscript does not currently meet the acceptance criteria for this journal. To improve the manuscript for further review, the authors are strongly advised to address the following points:

1. Enhance Writing Quality: Improve the clarity and logical structure of the text to enhance readability and ensure that the scientific rationale is effectively communicated.
2. Refine Experimental Details: Provide detailed and precise descriptions of experimental conditions, sample sizes, and statistical methodologies to ensure the reproducibility of the findings and strengthen the validity of the study.
3. Deepen Mechanistic Insights: Conduct a more thorough investigation of the mechanisms underlying the observed experimental phenomena, with a focus on functional experiments and

mechanistic analysis, to improve the study's scientific persuasiveness. Only after substantial improvements in these areas will the manuscript be deemed suitable for further evaluation.

We thank the reviewer for highlighting these important issues. All three points have been carefully addressed in the revised manuscript. First, we have thoroughly revised the text to improve clarity, coherence, and the manuscript has been professionally edited by an English language editor. Second, we have refined and expanded the descriptions of experimental conditions, sample sizes, and statistical analyses throughout the Material and Methods, Results, and Figure legends to ensure reproducibility and rigor. Third, we have incorporated additional experimental data that provide deeper mechanistic insights into SIRT7 function. Further details are provided below on a point-by-point basis.

In the peer-review process, I found that the manuscript still has some room for improvement in terms of experimental design and data analysis, particularly in the aspects of confirmatory experiments and mechanism exploration. Based on the current study's shortcomings, it is recommended that the authors supplement the following experiments.

1. A systematic comparison of in vitro and in vivo models: Based on the in vitro culture of GC2 cells, design in vivo experiments to validate the observed changes in H3K36ac and their effects on chromatin accessibility and DNA damage repair, ensuring consistency in results between in vitro and in vivo settings.

We thank the reviewer for raising this excellent point. To validate *in vivo* the phenotypes observed in *Wt* and *Sirt7$^{-/-}$* GC-2spd(ts) cells (now in Fig. 5d, e and Extended Data Fig. 4g-j), we performed several experimental approaches using spermatogonia-enriched samples and organotypic testis cultures.

To explore the link between H3K36ac and chromatin accessibility *in vivo* (now shown in Fig. 4 and Extended Data Fig. 4), we carried out H3K36ac ChIP-seq and ATAC-seq in *Wt* and *Sirt7$^{-/-}$* spermatogonia, a cell population in which SIRT7 is highly expressed (Fig. 3a, b and Extended Data Fig. 3a,b). We used post-natal day (Pnd) 5 testes, which are largely composed of undifferentiated spermatogonia, thereby allowing us to obtain sufficient biological material. In particular, *Wt* and *Sirt7$^{-/-}$* testes were harvested, dissociated, and cultured for 3 hours in α-MEM 10% KSR in 10-cm cell culture dishes. After this incubation period, the supernatant fractions, now enriched for PLZF^{+} spermatogonia (Extended Data Fig. 4a) were collected and processed for H3K36ac ChIP-seq and ATAC-seq. All experimental details are now provided in the Materials and Methods section, lines 437-524.

The results of these experiments are interesting and informative. Furthermore, the new *in vivo* data supports the *in vitro* findings, thereby strengthening our interpretations. In *Wt* spermatogonia, H3K36ac distribution was predominantly associated with promoter regions, as previously reported in somatic cells[1]. Consistent with immunofluorescence analysis in testicular sections (now Fig. 3c-e), H3K36ac levels were markedly increased upon SIRT7 loss (Fig. 4a), resulting in stronger signal intensity at pre-existing H3K36ac peaks and the emergence of *de novo* peaks at distal regulatory regions and introns (Extended Data Fig. 4b). ATAC-seq analysis indicated that the absence of SIRT7 also led to changes in chromatin accessibility (Fig. 4c-e and Extended Data Fig. 4f). Notably, in *Wt* spermatogonia, H3K36ac levels positively correlated with chromatin accessibility, and this correlation was further enhanced in the absence of SIRT7 (Fig. 4e). Importantly, this relationship was additionally supported by MNase assays in GC-2spd(ts) cells expressing a constitutive acetylated mutant H3^{K36Q}

mutant, which exhibited increased chromatin digestibility compared to the $H3^{K36Wt}$ form (now shown in Fig. 4f-h and Extended Data Fig.4k). Together, these findings indicate that SIRT7-dependent regulation of H3K36ac serves to restrict chromatin openness. These results are now described in the Results section, lines 213-227 and 239-246, Figure 4 and Extended Data Fig. 4, and corresponding Figure legends.

Regarding the DNA damage repair findings, we analyzed γH2AX in tissue cultures of *Wt* and *Sirt7*$^{-/-}$ testes in the presence or absence of doxorubicin (now included in Fig. 5a-c). For this approach, we again used testicular samples derived from Pnd 5 pups, ensuring the enrichment of spermatogonia, where SIRT7 is highly expressed (Fig. 3a, b and Extended Data Fig. 3a). In this context, doxorubicin exposure led to increased γH2AX in *Wt* and *Sirt7*$^{-/-}$ spermatogonia, which was higher in knockout samples. Notably, under basal conditions, *Sirt7*$^{-/-}$ testes also displayed significantly increased γH2AX staining intensity. Because *in vivo* analysis of γH2AX in 2–3-month-old *Wt* and *Sirt7*$^{-/-}$ testes (Fig. 5f, i), which are collected and immediately fixed without any culture step, showed no differences between genotypes, we reasoned that *Sirt7*$^{-/-}$ undifferentiated spermatogonia may be unable to effectively cope with the genotoxic stress that arises under standard culture conditions. Our results are broadly consistent with the results obtained in GC-2spd(ts) cells (now in Extended Data Fig. 5a), in which *Sirt7*$^{-/-}$ samples displayed increased γH2AX signal compared to controls. These new findings are included in the Results section, lines 249-262, Figure 5 and corresponding Figure legend.

Importantly, our new results are consistent between *in vitro* and *in vivo* settings, providing a strong link between SIRT7 and H3K36ac in controlling chromatin accessibility and DNA damage repair in primary cells.

2. Analysis of downstream target genes of H3K36ac: Employ techniques such as ChIP-seq to identify the target genes associated with H3K36ac-enriched regions, followed by functional experiments to validate the roles of these genes in spermatogenesis and reproductive aging in male germ cells.

We thank the reviewer for this valuable insight. To address this suggestion, we analyzed both the genome-wide distribution of H3K36ac and the transcriptome using ChIP-seq and RNA-seq approaches in *Wt* and *Sirt7*$^{-/-}$ spermatogonia-enriched samples (Fig. 4 and Extended Data Fig. 4). As mentioned above, in *Wt* spermatogonia, H3K36ac distribution was predominantly associated with promoter regions (Extended Data Fig. 4b), as previously reported in somatic cells[1]. Consistent with immunofluorescence analysis in testicular sections (Fig. 3c, d), H3K36ac levels were dramatically increased upon SIRT7 loss (Fig. 4a), resulting in stronger signal intensity at pre-existing H3K36ac peaks and the emergence of *de novo peaks* at distal regulatory regions and introns (Extended Data Fig. 4b).

Transcriptomic analysis revealed that *Sirt7* deficiency resulted in 26 significantly upregulated and 22 significantly downregulated transcripts in *Sirt7*$^{-/-}$ spermatogonia (Extended Data Fig. 4c), suggesting a modest role for SIRT7 in transcriptional regulation in this cell type. Importantly, and despite the importance of histone acetylation in gene activation, intersection of ChIP-seq and RNA-seq datasets showed that the excess of H3K36ac did not correlate with gene expression upregulation in *Sirt7*$^{-/-}$ cells (Extended Data Fig. 4d, e). Together, our results suggest that SIRT7-dependent H3K36ac deacetylation does not directly participate in transcriptional regulation. This new data is now included in a new results section entitled “SIRT7-dependent H3K36ac regulation is not essential for gene expression“ in lines 188-208. Additionally, we have incorporated all relevant experimental details into the Materials and Methods section, lines 437-524. Because this approach more directly assesses

H3K36ac compared to the previous whole testes RNA-seq approach, we have elected to replace that data with new ChIP-seq and ATAC-seq data in the revision.

3. Additionally, investigate the direct connection between H3K36ac and the Sirt7 gene.

Our work is among the firsts to investigate the *in vivo* role of SIRT7-mediated H3K36ac deacetylation. However, the deacetylase activity of SIRT7 has been extensively characterized by several groups using *in vitro* approaches. In particular, the connection between SIRT7 and H3K36 deacetylation was first demonstrated by Wang and colleagues (J Am Chem Soc, 2019)[2]. Using *in vitro* reconstituted acylated nucleosomes incubated in the presence or absence of SIRT7, the authors observed strong deacetylase activity of SIRT7 toward the H3K36ac residue. More recently, Moreno-Yruela and colleagues (Nat Comms, 2024)[3] further characterized the substrate specificity of SIRT7 toward this epigenetic mark at the structural level. Their work revealed that SIRT7 establishes multivalent interactions with the nucleosome through its N-terminal domain, while the catalytic domain binds to the nucleosome near the H3 tail exit, precisely where lysine 36 is located. This information, including references, is included in Results section, lines 135-139.

Specific suggestions and questions are listed below:

1. Revise the entire manuscript to enhance language clarity and precision, ensuring that all expressions are unambiguous and aligned with academic standards. Review the terminology to confirm its appropriateness and consistency with established academic norms. Optimize sentence structure to improve logical coherence and readability, ensuring seamless transitions between paragraphs to facilitate readers' understanding of the research logic.

We thank the reviewer for pointing out this issue. We have revised the whole manuscript to ensure clarity and coherence throughout the text. Also, and as mentioned above, the manuscript has been professionally edited by an experienced English language editor.

(a) The issue of tense consistency. In the Introduction section, the tense usage in the following sentence is inconsistent:

Original sentence: "This lack of attention is due to the long-held view that aging exerted a minimal impact on male fertility." Issue: The previous sentence uses the present tense ("the impact of aging on male germ cell development have not received comparable attention"), while the following sentence uses the past tense ("exerted"), which is inconsistent with the context. Suggestion: Change "exerted" to "exerts" to maintain tense consistency and better align with the common way of expressing ongoing phenomena in scientific fields.

We thank the reviewer for this point. In the revised version this sentence has been updated to one with better flow and clarity, which now reads as: “However, the impact of aging on male germ cell development has not received comparable attention, as its impact on fertility rates is not as pronounced as it is in women”(lines 43-45).

(b) Improper use of articles and terminology.

Original sentence: "During meiosis, spermatocytes complete a series of unique events, including double-strand break (DSB) formation, recruitment of DNA damage response (DDR) proteins, homology search, chromosome pairing, and, in some cases, resolution of DSB as crossover events." Issue: The definite article "the" is missing, and "DSB" should be in the plural form "DSBs." Suggestion: Change to "the resolution of DSBs as crossover events" to ensure grammatical accuracy and proper terminology.

We have changed “resolution of DSB as crossover events” to “the resolution of the DSBs as crossover” to maintain tense consistency, as appropriately noted by the reviewer. This change can be found in line 54.

2. The manuscript mentions that "aging exerted a minimal impact on male fertility.," but this viewpoint lacks sufficient scientific evidence or citation support.

Issue: Although the decline in male reproductive ability is slower, male fertility is indeed significantly affected with age. Stating that "aging has a minimal impact on male fertility" may not be entirely accurate and lacks supporting references.
Suggestion: Please provide relevant scientific evidence or cite authoritative literature to support this claim. Alternatively, you could rephrase it, such as stating "compared to women, male fertility is less affected by age, but it is not unaffected," and cite related references.

We apologize for the misunderstanding and thank the reviewer for their valuable comment. Our intention was indeed to indicate that male fertility is affected by age. We have rephrased the sentence as suggested by the reviewer which now reads: “However, the impact of aging on male germ cell development has not received comparable attention, as its impact on fertility rates is not as pronounced as it is in women” (lines 43-45). Additionally, we have included related references including Kong and colleagues (Nature, 2012)[4], Sharma and colleagues (Reprod Biol Endocrinol, 2015)[5] and Chico-Sordo and colleagues (Mech Ageing Dev, 2021)[6].

3. Inquiries regarding experimental design and data analysis: (a) The bar chart of "litter size" in Figure 1b remains unclear regarding the specific data represented by each circle. The authors are requested to provide detailed explanations, particularly clarifying whether the circles correspond to the litter-specific data shown in Figure 1c (L1-L7).

The datasets used in Figures 1b and 1c are the same. Figure 1b shows the mean litter number sired by *Wt* and *Sirt7*$^{-/-}$ males crossed with *Wt* females, with each circle representing an individual mating. The mean litter number is 7 for *Wt* males (experiment end-point) and 5 for *Sirt7*$^{-/-}$ males. Figure 1c depicts the cumulative number of pups sired by *Wt* and *Sirt7*$^{-/-}$ males, accounting for all litters from each mating. To avoid confusion, we rephrased the sentence in the results section which now reads “First, fertility studies were conducted by crossing wild-type (*Wt*) females with either *Wt* or *Sirt7*$^{-/-}$ males, then counting the number of litters and cumulative pup production (Fig. 1a-c)” (lines 92-93). Additionally, we have replotted Figure 1c (cumulative pup production) to display both the cumulative average pup production (continuous highlighted lines in graph) and the cumulative individual pup production (dotted lines) for each *Wt* and *Sirt7*$^{-/-}$ male. We have also updated the figure legend accordingly.

Notably, the WT x WT group in Figure 1b shows 4 points, while the WT x Sirt7-/- group shows 6 points, which appears inconsistent with the data presented in Figure 1c. The authors are asked to explicitly

explain the reasons for the sample size discrepancy between these two groups, whether it results from experimental design differences, data collection omissions, or other potential factors that may influence data presentation.

We thank the reviewer for pointing out the fact that the number of *Wt* x *Wt* crosses was different from *Wt* x *Sirt7*$^{-/-}$ crosses. During this revision, we have included two additional fertility trials using *Wt* males so that the number of matings analyzed is now identical for each genotype (n = 6 per genotype). Figure 1b and 1c and corresponding figure legends have been updated accordingly. As described above, Figure 1b shows the mean litter number sired by *Wt* and *Sirt7*$^{-/-}$ males with *Wt* females and Figure 1c reflects the cumulative pup production.

Additionally, it is crucial to ensure that the annotation method of data points aligns with the experimental design logic. Therefore, the authors are requested to thoroughly review whether Figure 1b and Figure 1c present data with logical inconsistencies. It is strongly recommended to clearly explain the relationship between the two figures and ensure the accuracy and consistency of the data to avoid potential misunderstandings by readers due to the apparent disparity in the number of points between the WT x WT and WT x Sirt7-/- groups in Figure 1b. Provide further details in results, figure legend and methodology (where apropiate).

We thank the reviewer for pointing out there may be a potential misunderstanding for readers. As described above, Figure 1b represents the average number of litters sired by *Wt* and *Sirt7*$^{-/-}$ males over the course of seven litters. *Wt* males produced a total of 7 litters (experimental end-point), whereas *Sirt7*$^{-/-}$ males produced an average of 5. Figure 1c shows the cumulative number of pups produced in each litter, which naturally increases as additional litters are produced. To avoid any confusion, we have rephrased the sentence which now reads "First, fertility studies were conducted by crossing wild-type (*Wt*) females with either *Wt* or *Sirt7*$^{-/-}$ males, then counting the number of litters and cumulative pup production (Fig. 1a-c)" (lines 92-93). We have also updated corresponding figure legends and included additional information in the Material and Methods to increase clarity (line 617).

(b) Issues with Figure 1f: Sample Size and Replicates. In Figure 1f, the number of samples and replicates for each group and time point is not clearly specified. To ensure the scientific rigor and reliability of the experiment, it is strongly recommended that the authors explicitly state the sample sizes for each group and time point. For example, if the WT group used 9 testes while the Sirt7-/- group only used 3 testes, this discrepancy in sample size may impact the accuracy of the experimental results. It is essential for the authors to explain the reasons behind this sample size imbalance and assess whether it could introduce potential biases in the statistical analysis outcomes.

We appreciate the reviewer's comment on this point. We have expanded our histological analysis in *Wt* and *Sirt7*$^{-/-}$ testicular sections which now corresponds to a n = 9 per genotype and age groups. Figure 1f and corresponding figure legend and Results sections (lines 105-117) have been updated.

Additionally, based on the statistical results in Figure 1f, 33.33% of mice in the 9-12 months Sirt7-/- group were categorized as "Mildly affected," whereas 40% of mice in the 4-6 months Sirt7-/- group fell into the same category. However, the morphological results presented in the corresponding supplementary figure do not fully align with these statistical findings. The authors are requested to provide a detailed explanation for this observed inconsistency.

We thank the reviewer for this important observation. Our new quantification (n = 9 per genotype) indicates that 22% and 11% of *Sirt7* $^{-/-}$ mice aged 4–6 months exhibit mild and severe testicular vacuolation, respectively. In contrast, 22% of testes from 9–11-month-old *Wt* males display mild

vacuolation. The previous image used for 4-6-month-old *Sirt7*$^{-/-}$ testis in Figure 1e was representative of the majority of testes of that group (60% in that moment). To avoid confusion, we have updated the image for this group, which now better represents the affected testis quantified in Fig 1f. To avoid any confusion, we have added arrowheads in the *Wt* image to highlight vacuolation in the aged group. We have also added additional images in Extended Data Fig.1a representing all histological groups (healthy, mildly and severely affected) identified in *Wt* and *Sirt7*$^{-/-}$ testes.

(c) Issues with Figure 1g: Statistical Details and Sample Size Discrepancy. In Figure 1g, the specific types of reproductive cells (e.g., spermatogonia, spermatocytes, sperm, etc.) being counted and the specific details of the statistical methods are not clearly stated by the authors. Additionally, there remains an inconsistency in the sample sizes between the WT group and the Sirt7-/- group. It is strongly recommended that the authors provide a detailed explanation of the statistical methods used and clarify the potential reasons for the observed sample size discrepancy.

We thank the reviewer for their careful revision and insightful comment. We have expanded our analysis, which now corresponds to n = 5 per genotype and age groups. Figure 1g and the corresponding Figure Legend have been updated. We have also included a dedicated section in Material and Methods describing the methods used to count germ cells in testicular sections (lines 413-418). Additionally, we have included the details of the statistical approaches used in each panel in the Figure legends.

(d) In Figure 1i, it is unclear whether the statistical analysis refers to the number of bright spots marked by white arrows in Figure 1h. Additionally, the authors are requested to clarify the criteria used to determine whether a sperm has "high DNA damage" and provide detailed information about the statistical approach applied in this analysis.

We thank the reviewer for requesting clarification on this point. Comet assays were analyzed using two complementary approaches, as shown in Figure 1h-j. First, the percentage of DNA in the comet tail was quantified using the CometScore software (TriTek Corp), which uses an algorithm to measure the amount of DNA present in the head and tail of each cell after gel electrophoresis. Cells analyzed with CometScore are now indicated by yellow arrows in Figure 1h and quantification is shown in Figure 1i. Highly damaged cells, however, are not detected by CometScore due to excessive DNA fragmentation and diffuse intensity signal that prevents accurate comet shape recognition by the software. Therefore, these cells were manually quantified and represented in Figure 1j. These cells are indicated with white arrows in Figure 1h. To avoid any confusion, we have rephrase the sentence in the Results section, which now reads: "Importantly, ~15% of sperm from 9-12-month-old Sirt7-/- mice presented a characteristic 'hedgehog' comet morphology, indicative of a severely damaged DNA (Fig. 1h, j)", in lines 129-130. Additional details of this analysis have been included in the Materials and Methods section (lines 431-435).

4. The basis for selecting the time points for transcriptome sequencing. In Figure 2, the authors performed transcriptome sequencing on testes from WT and Sirt7-/- mice, selecting the 2–3 months and 4–6 months points. However, based on the results shown in Figure 1, no significant differences were observed between the WT and Sirt7-/- groups at the 2–3 months time point, whereas significant differences were evident at the 4–6 month and 9–12 months time points.

Therefore, we request the authors to explain why these earlier time points (2–3 months and 4–6 months) were selected for transcriptome sequencing instead of the later time points (4–6 months and 9–12 months), which might better reflect the early features of reproductive aging in Sirt7-/- mice.

We thank the reviewer for this excellent observation. Because our bulk RNA-sequencing was performed on whole testis samples, we focused on young (2–3 months) and middle-aged (4–6 months) *Wt* and *Sirt7*$^{-/-}$ testes, as the cellular composition at these ages is comparable between genotypes. We reasoned that these stages would best capture the early molecular alterations preceding the testicular degeneration observed in *Sirt7*$^{-/-}$ animals.

During the revision of the manuscript, and in order to directly assess the role of SIRT7-dependent H3K36ac in transcriptional regulation, we performed bulk RNA-sequencing and ChIP-sequencing on spermatogonia-enriched samples from *Wt* and *Sirt7*$^{-/-}$ testes (Fig. 4 and Extended Data Fig. 4). These analyses indicate that SIRT7 and H3K36ac do not play a major role in gene transcription in these early stage spermatogenic cells.

Given these findings and the inherent difficulties of interpreting bulk RNA-sequencing from whole testis (previous Fig. 2), where cellular heterogeneity may mask cell-type–specific regulatory events, we have decided to remove the whole-testis RNA-seq dataset from the revised manuscript. We believe that focusing on spermatogonia-enriched populations provides a clearer and more biologically meaningful assessment of SIRT7's role in H3K36ac-mediated regulation.

Additionally, in later experiments, the authors analyzed acetylation levels at the 2–3 months and 9–12 months points. We kindly ask the authors to provide the rationale for selecting these specific time points and to clarify whether they considered age-related changes in testicular function when making these selections.

We thank again the reviewer for the opportunity to clarify this point. Following the initial characterization presented in Figure 1d-g using testes derived from young, middle-age and old animals, our subsequent analyses were conducted on testes from animals at 2–3 months (young) and 9–12 months (old). This allowed us to compare the epigenetic landscape of young testes with that of older animals, in which the phenotype was fully manifested in the *Sirt7*$^{-/-}$ testes. This rationale is now included in the Results section, lines 135-139.

5. The contradiction between the number of differentially expressed genes in transcriptome analysis and the morphological phenotype differences. In Figure 2b, the authors performed GSEA (Gene Set Enrichment Analysis) on the upregulated and downregulated genes between the WT and Sirt7-/- groups at the 2-3 months time point. However, no GSEA analysis was conducted on the differentially expressed genes in the 4-6 months group. Furthermore, the supplementary figures indicate that the number of differentially expressed genes in the 4-6 months group is fewer than in the 2-3 months group, despite the fact that the histomorphological phenotypic differences are more pronounced in the 4-6 months group. The authors are requested to provide a detailed explanation for why GSEA analysis was not performed on the 4-6 months group and to clarify how they interpret the phenomenon of decreasing numbers of differentially expressed genes with advancing age, particularly in light of the fact that knockout mice typically exhibit more pronounced phenotypes after reaching adulthood.

We thank the reviewer for this insightful and constructive observation. As correctly noted, GSEA was only performed on the 2-3-month-old samples, as no differentially expressed genes were detected in the 4-6-month-old group. Interestingly, histological degeneration in *Sirt7*$^{-/-}$ testes began at 4-6 months of age, a stage at which no significant transcriptional alterations were observed. Although we initially

discussed the possibility that early transcriptional changes in 2-3-month-old *Sirt7*$^{-/-}$ testes might influence later degeneration, we realized that interpreting bulk RNA-seq from whole testis samples (previous Fig. 2), which contain highly heterogeneous cell populations, is more complex than previously anticipated.

To maintain a clear mechanistic focus, in the revised manuscript we removed the whole-testis RNA-seq dataset (previous Fig.2 and previous Extended Data Fig. 2) and instead performed H3K36ac ChIP-seq and RNA-seq in *Wt* and *Sirt7*$^{-/-}$ spermatogonia-enriched samples. This approach enabled us to directly interrogate the role of SIRT7-dependent H3K36ac deacetylation in transcriptional regulation within a cell population where SIRT7 is highly expressed (Fig. 3a and Extended Data Fig. 3a). Differential expression analysis identified only 26 upregulated and 22 downregulated genes in *Sirt7*$^{-/-}$ spermatogonia, indicating that SIRT7 does not play a major role in transcriptional regulation at this developmental stage. Importantly, despite the dramatic increase in H3K36ac levels at promoter regions in *Sirt7*$^{-/-}$ cells (Fig. 4a and Extended Data Fig. 4b), transcriptomic analyses revealed no preferential gene upregulation neither in spermatogonia (Extended Data Fig. 4c) nor in 2-3-month-oold Sirt$^{-/-}$ whole testes (previous Fig. 2). These results strongly suggest that SIRT7 dependent H3K36ac deacetylation in this context functions outside classical transcriptional regulation. As already mentioned, previous Figure 2 and previous Extended Data Fig. 2 have been removed from the revised manuscript. New H3K36ac and RNA-seq datasets in spermatogonia-enriched samples are now included in new Figure 4 and Extended Data Fig. 4, corresponding Figure legends and Results sections (lines 188-208).

6. Discrepancy between acetylation levels and transcriptomic analysis results.In the manuscript, it is suggested that Sirt7 knockout leads to increased acetylation levels, yet in the transcriptomic analysis, no significant enrichment of pathways related to acetylation was observed. The authors are requested to explain this discrepancy.

We thank the reviewer for raising this important point. As discussed earlier, and in order to maintain a coherent narrative in the manuscript, we have removed the RNA-seq dataset generated from whole testicular samples. In the revised submission, we instead provide RNA-seq data from *Wt* and *Sirt7*$^{-/-}$ spermatogonia, which offer a more accurate vision of the transcriptomic consequences of SIRT7 loss in the cell population where it is most highly expressed.

SIRT7 is a histone deacetylase[2,7] and, as expected, its transcript was among the most significantly downregulated genes in our new analysis (Extended Data Fig. 4c). Lysine acetylation is known to influence chromatin architecture and is often associated with transcriptional activation particularly when enriched at promoters. However, despite the marked accumulation of H3K36ac at gene promoters in *Sirt7*$^{-/-}$ testes, we did not observe activation of acetylation-related pathways or broad transcriptional upregulation. This is consistent with our conclusion that SIRT7-dependent regulation of H3K36ac does not play a major role in transcriptional regulation in spermatogonia. The limited number of deregulated genes identified in the absence of SIRT7 further supports this interpretation.

Furthermore, in the transcriptomic analysis, the chromatin-associated factors in the testes of Sirt7-/- mice at the 2-3 months time point were primarily associated with methylation rather than acetylation, despite the subsequent WB (Western Blot) experiments showing no significant changes in methylation levels. The authors are kindly asked to provide a detailed explanation for this inconsistency and to explore potential underlying mechanisms, as well as to consider whether alternative regulatory mechanisms might be at play.

This is an excellent observation. Although global levels of H3K9me3 remain unchanged, as shown in our Western Blot analysis (Extended Data Fig. 2a), further characterization using high-resolution immunofluorescence reveals that the distribution of H3K9me3 is altered in *Sirt7$^{-/-}$* germ cells (data not shown). While these findings are indeed intriguing, we consider them beyond the scope of the current manuscript. In fact, we are actively investigating the potential role of SIRT7 in regulating the spatial organization of this epigenetic mark and its possible crosstalk with H3K36ac-mediated regulation. We hope to publish these results in the near future. Nevertheless, we have included a sentence in the discussion section to acknowledge the importance of these findings (lines 346-348).

7. Impact of Sirt7 knockout on somatic cells. In the transcriptomic analysis, the Leydig Cell Dysfunction pathway showed significant enrichment. The authors are kindly requested to address whether they considered the potential impact of Sirt7 knockout on testicular somatic cells, such as Leydig cells, and to explore whether the changes in these somatic cells might indirectly affect spermatogenesis.

We thank the reviewer for this important observation. Leydig cells are responsible for testosterone production, which is essential for supporting spermatogenesis through multiple mechanisms, including the maintenance of Sertoli cell function[8]. To evaluate the potential impact of SIRT7 deficiency on the somatic cell compartment, we undertook two approaches including the measurement of serum testosterone levels, and the quantification of Sertoli cell numbers.

We analyzed testosterone levels in serum samples of 2–3 and 9–11-months-old *Wt* and *Sirt7$^{-/-}$* male mice. Although the results do not show statistically significant differences between genotypes at either age group (see figure below), there was considerable inter-individual variability in the *Sirt7$^{-/-}$* older cohort, precluding drawing consistent conclusions. Unfortunately, due to time constraints and limited available biological material, we were unable to repeat the measurements with additional serum samples and therefore we prefer not to include these data in the manuscript.

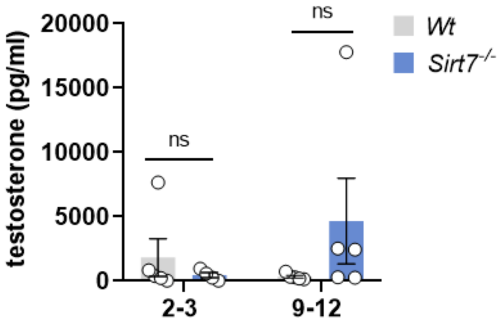

Levels of serum testosterone levels in *Wt* and *Sirt7$^{-/-}$* animals. Serum was obtained after blood samples were collected in tubes for serum collection (Sarsted, 32.311.001), left for 30 min at RT to allow clotting and centrifuged at 2500 g for 10 min. Serum was aliquoted and kept at -80ºC until processed for analysis. "Laboratori d'Anàlisi d'Indicadors Hormonals, d'Estrès, Benestar i Reproducció Animal" from the Autonomous University of Barcelona performed the ELISA tests for 5 samples per group. 1 sample from the 2-3-month-old group was omitted due to failed detection. Two-way ANOVA was used to test statistical significance.

Regarding Sertoli cells, we quantified their number in testicular sections from 9–11-month-old *Wt* and *Sirt7$^{-/-}$* mice using immunostaining for SOX9, a specific Sertoli cell marker[9]. Our analysis shows that Sertoli cell numbers are similar between *Wt* and *Sirt7$^{-/-}$* testes, further supporting a direct role for SIRT7 in germ cells rather than an indirect effect mediated by somatic support cells. These results are now included in Results section, lines 118-120 and in Extended Data Fig. 1c,d.

8. Mechanism of Sirt7 in Spermatogonia and Spermatocyte Function. The authors observed that Sirt7 exhibits high expression levels in spermatogonia and spermatocytes and identified its critical role in maintaining the population of undifferentiated spermatogonia cells during aging. Notwithstanding these observations, the study does not provide a detailed elucidation of the specific molecular mechanisms by which the Sirt7 gene maintains the population of undifferentiated spermatogonia. Nevertheless, the study fails to provide a detailed explanation of the specific molecular mechanisms of the Sirt7 gene in maintaining the population of undifferentiated spermatogonia, nor does it reveal its potential role in the events of spermatocyte meiosis during prophase.

We thank the reviewer for highlighting this important question. Indeed, understanding the molecular mechanism by which SIRT7 preserves the undifferentiated spermatogonia pool and supports spermatocyte progression is central to understanding its role in testicular aging. To address this, we have performed different experiments, including H3K36ac ChIP-seq, RNA-seq and ATAC-seq in spermatogonia-enriched populations (Fig. 4 and Extended Data Fig. 4) and organotypic testis cultures to analyze DNA-damage accumulation upon stress (Fig. 5).

First, because SIRT7 expression is highest in spermatogonia (Figure 3a and Extended Data Fig. 3a) and because H3K36ac levels are constitutively elevated in *Sirt7*$^{-/-}$ testes (Fig. 2 and Fig. 3b), we performed spermatogonial enrichment from Pnd 5 *Wt* and *Sirt7*$^{-/-}$ testes. Neonatal testes are enriched for spermatogonia, allowing us to isolate sufficient material to analyze the genome wide distribution of H3K36ac and its correlation with transcriptomics and chromatin accessibility changes. All details of this experimental approach are included in the Material and Methods section, lines 437-524.

H3K36ac genome-wide profiling revealed that in *Wt* spermatogonia, this mark is primarily enriched at gene promoters (Extended Data Fig. 4b). As expected, H3K36ac levels were significantly higher in *Sirt7*$^{-/-}$ spermatogonia (Fig. 4a). This increase took place not only at promoters already bearing H3K36ac but also led to the *de novo* appearance of H3K36ac at distal regulatory regions and introns (Extended Data Fig. 4b).

Despite this prominent increase of H3K36ac at gene promoters, differential gene expression analysis of the same samples revealed only a very small number of dysregulated genes in *Sirt7*$^{-/-}$ spermatogonia (Extended Data Fig. 4c). Importantly, the pronounced gain of H3K36ac at promoter regions did not result in transcriptional activation, indicating that H3K36ac does not function as a major driver of gene expression in this context (Extended Data Fig. 4d, e). These results strongly suggest that SIRT7 dependent H3K36ac deacetylation plays a role distinct from classical transcriptional regulation, which are now included in Results section, lines 188-208.

Given that studies in yeast have implicated H3K36 acetylation in promoting chromatin accessibility[10], we next examined chromatin accessibility in primary *Wt* and *Sirt7*$^{-/-}$ spermatogonia using ATAC-seq. Differential accessibility analysis identified 417 regions with increased accessibility and 249 regions with decreased accessibility in *Sirt7*$^{-/-}$ cells (Fig. 4c). To assess whether H3K36ac contributes to these changes, we compared mean H3K36ac signal with mean ATAC-seq signal across genomic regions from both genotypes. In both *Wt* and *Sirt7*$^{-/-}$ spermatogonia, H3K36ac positively correlated with chromatin accessibility, indicating that regions enriched for H3K36ac tend to exhibit higher accessibility (Fig. 4e). Notably, linear regression analysis revealed a modest but significant difference in slope between genotypes, suggesting that loss of SIRT7 enhances the coupling between H3K36ac and chromatin openness. These new results are now included in the Results section, lines 213-227.

Given these findings, and the absence of broad transcriptional changes in *Sirt7*$^{-/-}$ spermatogonia (Extended Data Fig. 4c), we looked for alternative mechanisms that might explain the premature loss of undifferentiated spermatogonia (Fig. 5f, g). In yeast, an acetylation switch in H3K36ac participates in efficient DNA damage repair pathway choice and increases homologous recombination[10]. Therefore, we hypothesized that chromatin accessibility changes in *Sirt7*$^{-/-}$ spermatogonia may alter DNA damage repair dynamics and may predispose them to DSB accumulation. To test this hypothesis, we cultured *Wt* and *Sirt7*$^{-/-}$ testes in the presence of the genotoxin agent doxorubicin. Under organotypic culture conditions, basal levels of γH2AX were significantly higher in *Sirt7*$^{-/-}$ testes, which were further exacerbated upon doxorubicin exposure compared to *Wt* controls. These data suggest that SIRT7 is required to maintain genome integrity under stress conditions, potentially by facilitating correct DNA-repair dynamics during spermatogonial proliferation and during spermatocyte prophase I. These new results are included in Fig. 5a-c, corresponding Figure legend and Results section, lines 249-262.

Together, our results point to a mechanistic model in which loss of SIRT7 leads to constitutive H3K36 hyperacetylation without major transcriptional consequences, impaired chromatin accessibility and increased DBS accumulation upon environmental stress, which likely compromise the homeostasis of undifferentiated spermatogonia and the successful completion of meiotic prophase I during aging.

Reviewer #2 (Remarks to the Author)

In this manuscript, Guitart-Solanes et al. investigated effects of depletion of Sirtuin 7 (SIRT7) gene in spermatogenesis. First, they found that loss of SIRT7 causes premature male reproductive defects and results in transcriptomic changes resembling premature aging. Furthermore, SIRT7 regulates H3K36ac in spermatogonia and spermatocytes. Lastly, they showed SIRT7 absence results in DNA damage accumulation in undifferentiated spermatogonia and impairs meiotic progression in an age-dependent manner. They concluded that SIRT7 links H3K36ac epigenetic regulation with genome maintenance in the aging mouse testis. The study revealed the function of SIRT7 in the spermatogenesis. However, the conclusion concerning the age-dependent function is not necessarily supported by their data. The following points should be addressed.

Major points:

1. The author declared that SIRT7 links H3K36ac epigenetic regulation with genome maintenance in the aging mouse testis, however, I cannot see significant difference in the expression of H3K36ac levels with age in the figure 3A.

We thank the reviewer for raising this important point. Please note that, Figure 3 is now Figure 2 in the revised manuscript. As correctly noted, the new Figure 2a shows no apparent differences in H3K36ac levels with age in *Wt* samples. However, these results are based on Western Blot analyses using protein extracts from whole testicular tissue, which includes both germ cells and somatic cells. Our data suggest that H3K36ac levels increase specifically in germ cells, with the most pronounced effect observed in spermatogonia (Fig. 3h). This cell-type specificity, in addition to the fact that spermatogonia abundance is low in adult testes, may be masking spermatogonia-specific effects in whole tissue extracts, explaining the apparent lack of difference in the Western blot showed in Figure 2a.

2. The author found that H3K36ac expression increased in pachynema and to a higher extent in diplonema in WT spermatocytes. Thus, GC-2 cell line was not appropriate in this study. Because The GC-2 cell line may be arrested in a premeiotic stage of differentiation, it was not recommended in the current study.

We agree with the reviewer that GC-2spd(ts) cells are transformed cells and may not reflect the molecular mechanisms occurring in primary cells. Additionally, GC-2spd(ts) cells divide mitotically and therefore are not suitable for studying meiotic events, such as those taking place in spermatocytes. To address the reviewer's concern, we repeated key experiments, originally performed in this cell line, in *Wt* and *Sirt7*$^{-/-}$ spermatogonia-enriched samples (Extended Data Fig. 4a), including chromatin accessibility assays (Fig. 4b-e) and the response to environmental stress (Fig. 5a-c).

As also discussed with Reviewer 1, because SIRT7 expression is high in spermatogonia (Fig. 3a,b and Extended Data Fig. 3a-d) and because H3K36ac levels are constitutively elevated in *Sirt7*$^{-/-}$ testes (Fig. 2 and Fig. 3c-e), we isolated spermatogonia from Pnd 5 *Wt* and *Sirt7*$^{-/-}$ testes. Neonatal testes are largely composed of spermatogonia, allowing us to isolate sufficient material to analyze the genome-wide distribution of H3K36ac and its correlation with transcriptomics and chromatin accessibility changes. All experimental details are included in the Material and Methods section, lines 437- 524.

Using this system, we first mapped the genome-wide distribution of H3K36ac in enriched spermatogonia samples. In *Wt* cells, H3K36ac was predominantly enriched at gene promoters (Extended Data Fig. 4b). Consistent with Western blot and immunofluorescence results (Fig. 2 and Fig. 3), *Sirt7*$^{-/-}$ spermatogonia displayed a global increase in H3K36ac (Fig. 4a), characterized by higher peak intensity at promoter regions and the appearance of *de novo* peaks at distal regulatory regions and introns (Extended Dat Fig. 4b). Despite this prominent increase of H3K36ac at gene promoters, differential gene expression analysis of the same samples revealed only a very small number of dysregulated genes in *Sirt7*$^{-/-}$ spermatogonia (Extended Data Fig. 4c). Importantly, the pronounced gain of H3K36ac at promoter regions did not result in transcriptional activation (Extended Data Fig. 4d, e), indicating that H3K36ac does not function as a major driver of gene expression in this context. These results strongly support that SIRT7 dependent H3K36ac deacetylation plays a role distinct from classical transcriptional regulation. H3K36ac ChIP-seq and RNA-seq findings are now included in Figure 4 and Extended Data Fig. 4a-e, corresponding Figure legend and Results section, lines 188-208.

Because we identified changes in chromatin accessibility in *Wt* and *Sirt7*$^{-/-}$ GC-2spd(ts) cells (now Extended Data Fig. 4g-j), we next conducted ATAC-seq in primary *Wt* and *Sirt7*$^{-/-}$ spermatogonia. Comparison of mean H3K36ac and ATAC-seq signals across genomic regions showed that, in both *Wt* and *Sirt7*$^{-/-}$ spermatogonia, H3K36ac positively correlated with chromatin accessibility, indicating that regions enriched for H3K36ac tend to be more accessible (Fig. 4e). Notably, linear regression analysis revealed a modest but significant difference in slope between genotypes, suggesting that loss of SIRT7 enhances the coupling between H3K36ac and chromatin openness. These results are now included in Figure 4b-e and Extended Data Fig. 4f, corresponding Figure legend and Results section, 213-227.

Finally, we tested whether the increased γH2AX formation observed under environmental stress in *Sirt7*$^{-/-}$ GC-2spd(ts) cells (now in Extended Data Fig. 5a) also occurred in primary germ cells (new Fig. 5a-c). To this end, we cultured *Wt* and *Sirt7*$^{-/-}$ neonatal testes in the presence of the genotoxic agent doxorubicin. Basal γH2AX levels were significantly higher in *Sirt7*$^{-/-}$ spermatogonia and were further

exacerbated upon doxorubicin exposure compared with *Wt* controls (Fig. 5 a, b). Notably, we also observed a decrease in total number of $PLZF^{+}$ undifferentiated spermatogonia after treatment (Fig. 5c). These findings indicate that SIRT7 is required to maintain genome integrity under stress conditions in both germ cell lines and primary germ cells. All these results are now included in the Results section, lines 249-262, Figure 5a-c and corresponding Figure legend, and Material and Methods section, lines 526-532.

This approach allowed us to validate the previous results in GC-2spd(ts) cells in primary spermatogonia. As accurately noted by the reviewer, our results show that SIRT7 is also highly expressed in primary spermatocytes (Fig. 3a, b), where lack of SIRT7 and aging lead to H3K36ac deregulation (Fig.3 c-e and Extended Data Fig. 3i, j). In this study, the lack of *in vitro* models to modulate meiosis and the limited amount of animal samples precluded us to further characterize the role of SIRT7-dependent H3K36ac regulation in the progression of meiotic prophase I. Future experiments will be needed to fully understand the contribution of SIRT7 during meiosis.

3. The author should check the expression of SIRT7 in the testicular sections using IF staining but not the analysis based on scRNA-seq.

We thank the reviewer for highlighting this important issue. Unfortunately, despite testing several commercially available antibodies, our unpublished analyses indicate that current SIRT7 antibodies are not suitable for immunofluorescence and immunohistochemistry, as signal is observed in control knockout samples (see figure below). To address this limitation, we have analyzed SIRT7 protein levels during the first wave of spermatogenesis by Westen blot. Because developmental staged populations (spermatogonia, spermatocytes, spermatids and sperm) appear in a timely manner during the first wave, we collected testis samples at different Pnd, including Pnd 5 and Pnd 9 (containing mostly spermatogonia), Pnd 18 (containing spermatogonia and early spermatogenic cells), Pnd 24 (when round spermatids appear) and Pnd 35 (when elongated sperm appears). Consistent with the scRNA-seq analyses (now in Extended Data 3a-d), Western blot results show that SIRT7 is highest at Pnd 5, 9, and 18 and declines at Pnd 24 and 35, again suggesting a role for SIRT7 in early germ cell development. Using this approach, we have also observed an inverse pattern for H3K36ac levels, which were low at early stages (Pnd 5 and Pnd 9) but gradually increased with spermatogenesis progression. Western blot results are now included in Figure 3a,b and in the results section, lines 156-163.

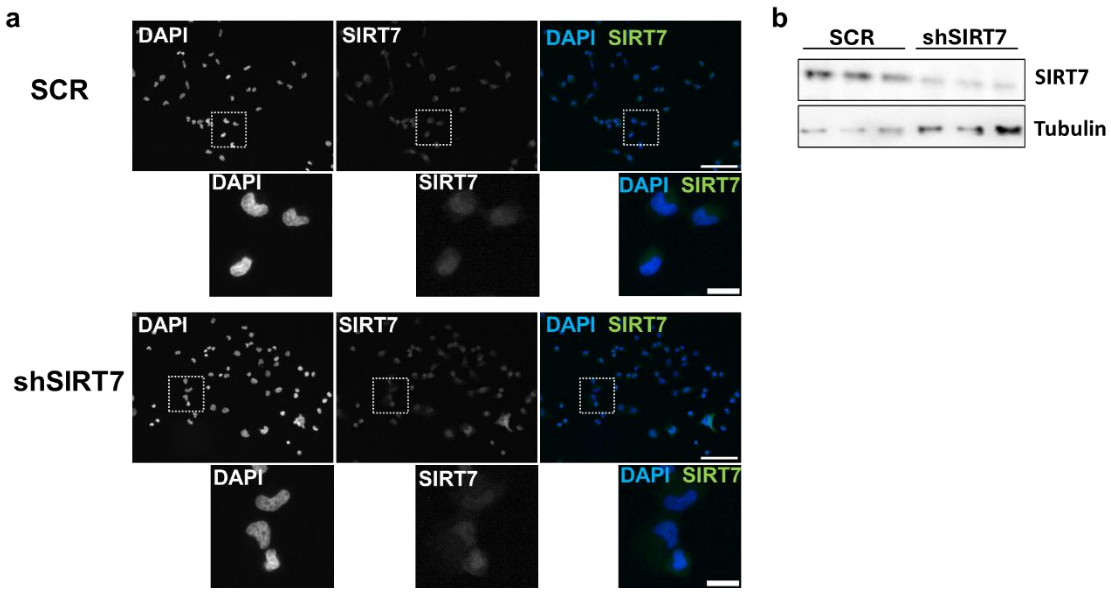

Immunofluorescence of SIRT7 in SIRT7 knock-down H1299 cells. **a)** Immunofluorescence of SIRT7 (Abcam, ab259968 1:100) in H1299 cells after infection with Scrambled hairpin RNA (SCR) or shSIRT7. Scale bars = 100 µm and 20 µm (zoom in). **b)** Western blot of 3 different H1299 clones, confirming the reduction in SIRT7 levels after shRNA introduction.

4. The author declared that 9-12-month-old Sirt7$^{-/-}$ testes showed defective DNA damage repair and impaired meiotic progression. Does this deficiency could activate of DNA damage checkpoint, which could result in meiotic arrest?

We thank the reviewer for this insightful comment. To address the reviewer's question, we analyzed the expression of the histone variant H1T, a testis-specific linker histone variant expressed from mid pachynema onwards only if DSB are processed[11]. Analysis of meiotic populations using γH2AX, SYCP3 and H1T staining revealed an increase of H1T^{-} spermatocytes in 9-12-month-old *Sirt7*$^{-/-}$ testes, indicating a developmental arrest or delay in early pachynema (Fig 6h and Extended Data Fig 6b). Additionally, no increase in γH2AX signal outside the XY body was found in H1T^{+} *Sirt7*$^{-/-}$ cells compared with *Wt* controls. Together, these results suggest the existence of intact surveillance mechanisms in *Sirt7*$^{-/-}$ spermatocytes. These results are now included in the Results section, lines 297-302, Figure 6h and Extended Data Fig. 6b and corresponding Figure legends

5. Line 234 I cannot see any significant accumulation of γH2AX in undifferentiated spermatogonia with age in the figure 6c first and third panel.

We thank the reviewer for the careful evaluation. We have updated the figure image (now Fig. 5h), which now more accurately represents the quantification of the increase in γH2AX in *Wt* undifferentiated spermatogonia (now Fig. 5i).

6. In the figure 7A, is there almost 10% of pachytene-like spermatocytes in the WT testis with 2-3 months old? The percentage was too high in the WT testis with 2-3 months old.

We greatly appreciate the reviewer's insightful comments on this point. The reviewer is correct that pachytene-like cells shouldn't be present in *Wt* testes. In our initial analysis, we defined "pachytene-

like" cells as spermatocytes displaying synapsed SYCP3 axes together with high γH2AX signal in autosomes. However, further meiotic progression examination using detection of the testis-specific histone variant H1T, which is expressed from mid-pachynema onwards, revealed that these cells were negative for H1T (Fig. 6h and Extended Data Fig. 6b). This indicates that, rather than representing true pachytene-like cells, they most likely correspond to early pachytene spermatocytes which are undergoing DSB repair. Accordingly, we have updated the labelling of meiotic populations throughout Figure 6a-c, which now reads as early pachynema, and Results section (lines 284-291).

7. The author should check the CO formation and synapse in the meiosis using MLH1 and SYCE1 or SIX6OS1 antibodies.

We thank the reviewer for this valuable suggestion. We analyzed MLH1 foci and SYCE1 in spermatocytes from 9-11-month-old *Wt* and *Sirt7*$^{-/-}$ mice. Regarding MLH1, *Wt* spermatocytes displayed an average of 23 MLH1 foci, whereas *Sirt7*$^{-/-}$ spermatocytes showed a significant reduction, with an average of 18 foci (Fig. 6i, j). This result suggests that SIRT7 deficiency is associated with partial defects in crossover formation in spermatocytes, similar to findings in oocytes[12]. These results are now included in the Results section, lines 302-304, Figure 6i, j and the corresponding Figure legend. Regarding the analysis of synaptonemal complex formation, we analyzed the central protein SYCE1 in spreads derived from 9-11-month-old *Wt* and *Sirt7*$^{-/-}$ mice (Extended Data Fig. 6a). SCYE1 was detected along autosomes with fully synapsed SYCP3 axes, as well as in the pseudoautosomal region of the sex chromosomes in both genotypes suggesting that the formation of the synaptonemal complex central element is not affected in the absence of SIRT7. SCYE1 results are now included in the Results section, lines 294-297, Extended Data Fig. 6a and the corresponding Figure legend.

8. The author did not decipher the mechanism of high sperm DNA fragmentation in the sperm of SIRT7-/- epididymis. The DNA damage accumulation in undifferentiated spermatogonia or defective DNA damage repair in the meiosis? For the expression of SIRT7 was very low in the spermatids.

This is an excellent observation. Our results indicate that SIRT7 deficiency is associated with genomic instability in both spermatogonia and spermatocytes (Fig. 5 and Fig. 6). On one hand, we found that undifferentiated *Sirt7*$^{-/-}$ spermatogonia were more susceptible to environmental stress and declined prematurely compared with *Wt* controls (Fig. 5). On the other hand, we also observed a partial meiotic prophase I arrest at early pachynema and defective crossover formation in older *Sirt7*$^{-/-}$ spermatocytes (Fig. 6). Together, these findings suggest that the DNA fragmentation observed in *Sirt7*$^{-/-}$ sperm may originate at multiple developmental stages of spermatogenesis.

However, determining the precise developmental origin of this damage is a complex question that will require future studies. In particular, stage-specific conditional mouse models, such as Ddx4-Cre or Spo11-Cre–mediated *Sirt7* deletion, will be essential to dissect the distinct contributions of H3K36ac deregulation and genome instability arising specifically in spermatogonia versus spermatocytes to the overall aging phenotype.

As mentioned by the reviewer, the expression of SIRT7 is low in spermatids (Fig. 3a and Extended Data Fig. 3a), which suggests that spermiogenesis may not be affected by the absence of SIRT7. Our results, however, indicate that H3K36ac is mildly deregulated in *Sirt7*$^{-/-}$ spermatids (Fig. 3h), potentially impacting the process of chromatin remodeling and compaction. Although addressing these mechanistic questions is beyond the scope of the current manuscript, we now explicitly acknowledge this point in the Discussion section, lines 357-359 and 373-381.

Minor points:

We really appreciate the reviewer's careful assessment.

Line 160: western blot should be Western blot.

western blot has been substituted for Western blot

Line 181 "in" should be in "Times New Roman" format

The format in this item has been changed to Times New Roman.

Line 225-236 the paragraph is not justified

The paragraph is now justified.

REFERENCES

1 Lu, X. *et al.* Loss of LOXL2 Promotes Uterine Hypertrophy and Tumor Progression by Enhancing H3K36ac-Dependent Gene Expression. *Cancer Res* **82**, 4400-4413, doi:10.1158/0008-5472.CAN-22-0848 (2022).

2 Wang, W. W. *et al.* A Click Chemistry Approach Reveals the Chromatin-Dependent Histone H3K36 Deacylase Nature of SIRT7. *J Am Chem Soc* **141**, 2462-2473, doi:10.1021/jacs.8b12083 (2019).

3 Moreno-Yruela, C. *et al.* Structural basis of SIRT7 nucleosome engagement and substrate specificity. *Nat Commun* **16**, 1328, doi:10.1038/s41467-025-56529-y (2025).

4 Kong, A. *et al.* Rate of de novo mutations and the importance of father's age to disease risk. *Nature* **488**, 471-475, doi:10.1038/nature11396 (2012).

5 Sharma, R. *et al.* Effects of increased paternal age on sperm quality, reproductive outcome and associated epigenetic risks to offspring. *Reprod Biol Endocrinol* **13**, 35, doi:10.1186/s12958-015-0028-x (2015).

6 Chico-Sordo, L. *et al.* Reproductive aging and telomeres: Are women and men equally affected? *Mech Ageing Dev* **198**, 111541, doi:10.1016/j.mad.2021.111541 (2021).

7 Barber, M. F. *et al.* SIRT7 links H3K18 deacetylation to maintenance of oncogenic transformation. *Nature* **487**, 114-118, doi:10.1038/nature11043 (2012).

8 Zhou, R. *et al.* The roles and mechanisms of Leydig cells and myoid cells in regulating spermatogenesis. *Cell Mol Life Sci* **76**, 2681-2695, doi:10.1007/s00018-019-03101-9 (2019).

9 Sekido, R., Bar, I., Narvaez, V., Penny, G. & Lovell-Badge, R. SOX9 is up-regulated by the transient expression of SRY specifically in Sertoli cell precursors. *Dev Biol* **274**, 271-279, doi:10.1016/j.ydbio.2004.07.011 (2004).

10 Pai, C. C. *et al.* A histone H3K36 chromatin switch coordinates DNA double-strand break repair pathway choice. *Nat Commun* **5**, 4091, doi:10.1038/ncomms5091 (2014).

11 Inselman, A., Eaker, S. & Handel, M. A. Temporal expression of cell cycle-related proteins during spermatogenesis: establishing a timeline for onset of the meiotic divisions. *Cytogenet Genome Res* **103**, 277-284, doi:10.1159/000076813 (2003).

12 Vazquez, B. N., Blengini, C. S., Hernandez, Y., Serrano, L. & Schindler, K. SIRT7 promotes chromosome synapsis during prophase I of female meiosis. *Chromosoma* **128**, 369-383, doi:10.1007/s00412-019-00713-9 (2019).

We thank the reviewers for their constructive and insightful comments. We have carefully addressed all concerns and introduced new revisions that, in our view, substantially improve the coherence and overall quality of the manuscript. The most significant changes include a re-examination and clarification of prophase I and pachytene staging; a new analysis of meiotic recombination checkpoint activation; the addition of original Western blots; and the clarification of potential discrepancies in the age-dependent H3K36ac datasets and late spermatocyte population results.

All newly added or revised content is highlighted in yellow in the revised manuscript. We hope that the updated version will now be suitable for consideration for publication in *Nature Communications*.

Below are our point-by-point responses to reviewers. The reviewer's comments are in blue font, and our responses are in black font.

Reviewer #1 (Remarks to the Author):

1.There are still issues with unclear descriptions throughout the manuscript, which require a thorough review and revision. For example, in Figure 6C, what criteria are used to distinguish between early and middle pachytene stages? Based solely on the authors' presented results, I see no morphological distinction between the two stages. Moreover, the early pachytene stage shown is not typical. I suggest the authors refer to more literature to accurately define the morphology of early pachytene and clarify the criteria used to differentiate it from the mid-to-late stages.

Additionally, the authors should explain why the γH2AX signal on the XY chromosomes in Figure 6C appears diffuse rather than punctate. Is this an abnormal phenotype or due to other reasons? Please annotate this clearly.

We thank the reviewer for this important comment. The reviewer is correct that the early pachytene cell shown in the previous Figure 6c is not representative, as the γH2AX signal is diffuse, which is more characteristic of an earlier stage. To improve staging accuracy, we reanalyzed prophase I progression in *Wt* and *Sirt7*$^{-/-}$ spermatocytes by immunostaining for SYCP3 and γH2AX(Page et al., 2012), in addition to H1T(Inselman, Eaker, & Handel, 2003; Lam, Brick, Cheng, Pratto, & Camerini-Otero, 2019), whose expression begins in mid pachynema and is maintained onwards, allowing us to discriminate between early, mid, and late pachytene stages (new Fig. 6a–c and Extended Fig. 6a). Moreover, we used an additional criteria based on the positioning of sex chromosomes to improve discrimination between late zygotene and early pachytene stages as previously described(Page et al., 2012). The criteria used to define each prophase I substage is described in detail in Results section, lines 286-298, and representative images are now included in the new Extended Fig. 6a. The criteria used to identify now reads as: "Using co-immunostaining of γH2AX, SYCP3, a lateral element protein of the synaptonemal complex(Lammers et al., 1994), and H1T, a testis-specific linker histone that is expressed from mid-pachynema onwards(Inselman et al., 2003), we identified and quantified the distinct prophase I stages in *Wt* and *Sirt7*$^{-/-}$ testes (Extended Data Fig. 6a). In *Wt* spermatocytes, γH2AX signal is dispersed during leptonema and zygonema, coinciding with DSB formation and the onset of chromosome pairing. In early pachynema, autosomal SYCP3 axes are largely synapsed, although discrete γH2AX foci persist on some autosomes. At this stage, the sex chromosomes initiate synapsis through the pseudoautosomal region (PAR), which exhibit a

strong γH2AX enrichment, marking the onset of the sex body formation(Page et al., 2012). The developing sex body frequently displays an elongated configuration at this point. In mid pachynema, H1T expression is detectable for the first time and is further increased in late pachynema. Additionally, in mid and late pachynema, γH2AX disappears from autosomes and remains only in the sex body, displaying a rounded configuration and promoting meiotic sex chromosome inactivation(Fernandez-Capetillo et al., 2003). Finally, diplotene cells are identified by the appearance of discontinuous SYCP3 staining due to chromosome desynapsis, together with strong SYCP3 signal persisting at chromosome ends."

Based on this analysis, young *Wt* and *Sirt7*$^{-/-}$ spermatocytes displayed similar prophase I stages percentages (new Fig. 6a). Notably, 9–11-month-old *Sirt7*$^{-/-}$ spermatocytes had a significant increase in the percentage of zygotene and early pachytene cells (new Fig. 6b). These results are now included in Results sections, lines 299-306, Figure 6a-d and Extended Data Figure 6a.

2.The figure layout lacks rigor. For instance, the pachytene cell shown in Figure 6E is not representative and has poor morphology.

We are not completely sure which figures the reviewer is referring to, as Figure 6e in the previous version corresponded to a graph. However, if the reviewer was referring to the MLH1 Foci images (now Fig. 6j in the revised version), we felt this could be improved, and we have replaced the image with a higher-quality one accordingly.

In addition, multiple figures throughout the manuscript are unclear. Regarding Figure 4E, the authors did not clearly state which specific cell type the H3K36ac fluorescence intensity was measured from. This needs further clarification.

We thank the reviewer for raising this important point. We have carefully revised all figures and figure legends to ensure that the relevant information is clearly and accurately indicated. Regarding Figure 4e, the plot represents mean H3K36ac ChIP-seq peak signal versus the mean ATAC-seq peak signal in spermatogonia-enriched populations. Importantly, the H3K36ac signal shown in this figure correspond exclusively to ChIP-seq data and not to H3K36ac fluorescence microscopy data. To avoid any ambiguity, we have updated both the axis labels and the figure legend (line 924-926) to clearly include this information.

3.If the authors claim that knockout of this gene leads to increased H3K36ac levels and premature testicular degeneration, then why is the H3K36ac level in 9–12-month-old mice lower than in 2–3-month-old mice? According to the authors' theory, the H3K36ac level in 9–12-month-old knockout mice should be higher than in younger mice, but the current results show the opposite. Please provide an explanation.

The reviewer is correct that H3K36ac levels do not increase in lysates from 9–11-month-old *Sirt7*$^{-/-}$ mouse testes (Fig. 2a–b) compared to 2–3-month-old *Sirt7*$^{-/-}$ samples. However, these results are based on Western blot analyses performed on protein extracts from whole testicular tissue, which comprise a mixture of germ cells and somatic cells.

In contrast, our immunofluorescence analyses shown in Figure 3c-e specifically examine germ cell populations and reveal an age-associated increase in H3K36ac in *Sirt7*$^{-/-}$samples, with the most pronounced effect observed in spermatogonia. Notably, this age-dependent

increase is also observed in *Wt* samples (Fig. 3c-e and Extended Data Fig. 3j, k). This cell type–specific increase may be masked in whole-testis tissue extracts, particularly given the relatively low abundance of spermatogonia in adult testes, thereby explaining the apparent lack of difference detected by Western blot in Figure 2a.

Furthermore, although Western blotting shows an apparent decrease in H3K36ac levels in 9–12-month-old *Sirt7*$^{-/-}$ testes relative to 2–3-month-old *Sirt7*$^{-/-}$ samples, statistical analysis performed during this revision indicates that this decline is not significant and therefore may not represent a biologically meaningful reduction. To prevent any potential misinterpretation, we have removed the sentence "In 2–3-month-old *Sirt7*$^{-/-}$ testes, there was a significant ~6-fold increase in H3K36ac levels, compared with a significant ~4-fold increase in the testes of older mice (Fig. 2a, b)" from the revised manuscript. We have also updated Figure 2b to include this statistical analysis. Finally, we have now explicitly discussed the limitation of Western Blot results (Fig. 2) in the Discussion section, lines 343-345.

Furthermore, there is no significant difference in H3K36ac levels between 9–12-month-old and 2–3-month-old wild-type mice, yet the authors claim that SIRT7-regulated H3K36ac is age-related. Considering that testicular tissue contains both somatic and germ cells, the authors are advised to replace the testis-based Western blot experiment with one performed on spermatogonia specifically. This may yield more accurate results.

We thank the reviewer for this valuable suggestion. However, Western blot analysis requires a large number of cells, which is particularly challenging for spermatogonia. Based on our experience, FACS isolation of adult spermatogonia yields very low cell numbers, making it difficult to obtain sufficient material for protein extraction. Performing this experiment would therefore require pooling samples from multiple animals, which is not feasible within the time constraints of the revision.

Importantly, H3K36ac levels in spermatogonia are already shown by immunofluorescence in Figure 3c-e, directly addressing the reviewer's question and supporting our conclusions. The Western blot in Figure 2 was intended as an initial, broad epigenetic mark screen rather than a cell-type–specific analysis. For these reasons, we believe that adding the proposed Western blot experiment is not feasible at this stage, but we sincerely appreciate the reviewer's thoughtful suggestion. We have also clarified this rationale in the Results section, line 138.

In addition, please provide the original Western blot images used, indicate the molecular weight of the target proteins, and explain how the quantification was performed for proteins of similar size. Lastly, in Figure 3A, the authors refer to the expression of SYCP3 protein. However, SYCP3 is commonly known to appear as two bands, while the authors only show one.

We thank the reviewer for bringing up this important point regarding manuscript transparency. We have now included a new Source Data Figures named Unprocessed WBs containing all Western blots presented in the manuscript, with the corresponding molecular weights and the proteins analyzed on each membrane. In addition, we have added the molecular weights to all Western blot images shown in the manuscript (Fig. 2a, Fig. 3a, Fig. 5d, Extended Data Fig. 2a, Extended Data Fig. 3e, and Extended Data Fig. 5a).

Regarding the question about how proteins of similar molecular weight (such as H3K36ac and total H3) were analyzed, samples were run the same day in duplicate on two

independent gels and transferred to separate membranes. This information has now been added to the Materials and Methods section, lines 600-602.

Concerning SYCP3 analysis, the reviewer is correct that SYCP3 is expected to appear as two bands. The second band was not clearly visible in the original Western blot image due to low exposure time during membrane development. We have now replaced the initial image with a higher-exposure version that shows both bands (new Fig. 3a)

4.In the authors' response, it is stated that Sirt7 deficiency leads to impaired chromatin accessibility and increased accumulation of DSBs, thereby affecting spermatogonial homeostasis. The authors should explain the evidence supporting a link between the two them, according to existing results, no direct association between the two has been demonstrated.

We thank the reviewer for the opportunity to clarify this point. As noted, our current model suggests that SIRT7 deficiency leads to altered chromatin accessibility and increased accumulation of DSBs. The functional link between chromatin accessibility and DSBs is well established in the context of DNA damage repair signaling. Indeed, previous evidence indicates that chromatin relaxation occurs at early stages after DSB formation, which is then followed by chromatin re-compaction at later stages of the DSB repair process (Burgess, Burman, Kruhlak, & Misteli, 2014). Moreover, we have previusly shown that both histone acetylation and deacetylation are essential for efficient DSB repair (Vazquez et al., 2016), underscoring that chromatin accessibility is dynamic during the repair process.

[editorial note: confidential, unpublished information redacted]

Although these findings further suggest the proposed mechanistic link between chromatin accessibility defects and DSB accumulation in the absence of SIRT7, we believe that a

detailed mechanistic analysis falls beyond the scope of the current manuscript. Indeed, we are currently exploring these observations in greater detail and plan to publish them in a follow-up study. Nevertheless, we have included this important information in the Discussion section, lines 391-395.

5.How were the concentration of DOI? And the 16-hour treatment duration determined for the vivo testis culture experiments?

We thank the reviewer for this question. The concentration of doxorubicin (DOX) and the 16-hour treatment duration used in the *ex vivo* testis culture experiments (Fig. 5a-c) were selected based on previously established and well-characterized protocols in the field(Allen, Lopes, Mitchell, & Spears, 2018; Lopes et al., 2021; Smart et al., 2018). In particular, these experimental conditions have been extensively optimized and validated by the laboratory of the co-author Professor Norah Spears (University of Edinburgh), which has broad expertise in gonadotoxicity studies using prepubertal mouse gonads. The selected conditions were previously reported to induce measurable DNA damage while preserving spermatogonia survival in *Wt* tissues as previously reported by her group(Smart et al., 2018). This information is now included in the Material and Methods section, lines 552-554.

6. During months 9–12, KO mice exhibited a twofold increase in early pachytene stages and a reduction in middle and late stages relative to WT mice; however, why did the two groups show comparable diplotene stage counts?

As correctly pointed out by the reviewer, 9-11-month-old *Sirt7*$^{-/-}$ testes displayed a significant increase in early pachytene cells (new Fig. 6b), accompanied by a significant reduction in mid/late pachytene cells. The observation that the percentage of diplotene cells remains comparable between genotypes suggests that *Sirt7*$^{-/-}$ spermatocytes most likely undergo a developmental delay during early pachynema but eventually progress to subsequent stages. We believe that the number of *Sirt7*$^{-/-}$ spermatocytes reaching mid/late pachynema is sufficient to sustain normal progression to diplonema. These conclusions are now incorporated into the Results section, lines 315-318.

7.The study's investigation of late spermatocytes is insufficient and does not align well with earlier findings.

We thank the reviewer for this valuable comment. Our earlier findings (Fig. 1) indicate that *Sirt7*$^{-/-}$ mice display an age dependent subfertility (Fig. 1a-c) associated with an increase of sperm DNA fragmentation (Fig. 1h-j). As correctly noted by the reviewer, analysis of prophase I progression indicates that *Sirt7*$^{-/-}$ spermatocytes display a developmental delay (Fig. 6b) but finally progress to diplotene stage, as indicated by similar percentage of these cells in both genotypes. MLH1 foci, however, were mildly reduced in older *Sirt7*$^{-/-}$ spermatocytes (Fig. 6j, k) raising the possibility of potential defects at later stages, which were not reported in this manuscript. Additionally, our results indicate that H3K36ac is mildly deregulated in *Sirt7*$^{-/-}$ spermatids (Fig. 3e), potentially impacting the process of chromatin remodeling, compaction and DNA damage repair. Although addressing these questions is beyond the scope of the current manuscript, we now explicitly acknowledge this point in the Discussion section, lines 397-402.

8.The results seem more focused on showing that gene knockout–induced reproductive damage increases with age, rather than demonstrating a direct link to naturally aged mice.

We thank the reviewer for this thoughtful comment. We agree that our study primarily defines the role of SIRT7 in early germ cells and during gametogenesis. Our data demonstrate that SIRT7 deficiency impacts male reproductive fitness with age. However, although we provide evidence that H3K36ac, the epigenetic target of SIRT7, becomes deregulated during testicular aging (Fig. 3c-e), we do not directly test SIRT7 activity in naturally aged testis samples. To avoid overinterpretation, we have toned down our conclusions in the Discussion section and included a sentence acknowledging the importance of understanding the role of SIRT7-dependent H3K36ac regulation in the testes of naturally aged mice (lines 350-351). In addition, we have revised the title to better reflect the scope of the study and removed the term "aging". The revised title now reads: "SIRT7 links H3K36ac epigenetic regulation with genome maintenance in the mouse testis".

9.Evaluating somatic cell numbers alone is not sufficient to conclude that the knockout has no effect on somatic cells.

We agree with the reviewer that we cannot rule out a potential impact of SIRT7 deficiency on the somatic cell compartment. As correctly noted, although Sertoli cell numbers were similar between genotypes, functional alterations may still be present. We have now included a sentence in the Discussion section acknowledging this limitation (lines 380-382).

Reviewer #2 (Remarks to the Author):

The authors have mostly addressed my questions. However, several points should be addressed before publication.

1. Figure 1d: It was weird that the testicular weight in 9-12 month was heavier than that in 2-3 month.

We thank the reviewer for this important comment. In the previous version of the manuscript, testis weight results were derived from two independent SIRT7 mouse colonies: one housed at the CMCiB (Badalona, Spain) and the other at the Rutgers University animal facility (Piscataway, USA). Although both colonies display the same phenotype, a detailed reexamination of the data revealed that absolute testis weight in *Wt* animals was higher in the CMCiB colony. We believe these differences are most likely attributable to variations in husbandry conditions between the two facilities.

Importantly, the conclusions regarding testis weight in the previous version were influenced by the combined dataset, which was biased toward the colony that had recorded a larger number of measurements for each time-point and therefore did not accurately represent the trends within each individual colony. Because the dataset from the Rutger University animal facility includes a larger number of animals in the young, middle aged, and old groups, we decided to update Figure 1d with only the testis weight measurements from this colony. The updated Figure 1d shows that in *Wt* mice, testis weight significantly increases between 2–3 and 4–6 months of age, and then remains stable at 9–11 months, which is

consistent with previous reports (Endo et al., 2024). In addition, we have modified the text in the Results section accordingly (lines 101-104).

2. Figure 1e: right panel: “9-1-month-old”.

We thank the reviewer for the careful evaluation. The error in the panel of Figure 1e has been corrected to “9-11-month-old”.

3. Figure 3a: Please repeat the WB assay. For one thing, considerable within-group variability was observed. For another, the band (SIRT7) exhibits a hollow appearance. Furthermore, the expression of SIRT7 decreased sharply in Pnd 24 and Pnd 35. Please explain and add this part in discussion.

We thank the reviewer for this insightful comment. Due to time constraints, we were able to collect three additional independent samples at Pnd 18 and Pnd 24. Western blot analysis of these samples is consistent with our previous observations, showing a decline in SIRT7 protein levels coinciding with the onset of spermatid appearance. Additionally, no variability was observed within group samples. A similar decreasing trend is also observed for SYCP3 protein, although the decline is less pronounced. H3K36ac showed an opposite trend, being higher in testis samples from Pnd 24 mice. This new Western blot analysis has now been included in Extended Data Figure 3e. Because SIRT7 protein levels in the first wave of spermatogenesis are similar to those observed in scRNA-seq datasets from adult human and mouse testes (Extended Data Figure 3a-c), where *Sirt7* mRNA is highly expressed in spermatogonia and spermatocytes, we believe that the observed dynamics of SIRT7 protein likely reflects its predominant role in early germ cell populations. However, at this moment we cannot rule out the possibility that SIRT7 protein dynamics may differ between the first wave of spermatogenesis and subsequent spermatogenic cycles. We have now included this point in the revised Discussion section, lines 337-341.

Regarding the hollow appearance of the SIRT7 band in the original Western blot shown in Figure 3a, we repeated the experiment using the same protein samples. In the updated blot, the SIRT7 band appears clearly defined and no longer shows the hollow morphology previously noted. Importantly, the overall expression pattern remains fully consistent with the initial results. We have updated the Western blot panel accordingly, which is now presented as the new Figure 3a.

4. For the question “The author declared that 9-12-month-old Sirt7-/- testes showed defective DNA damage repair and impaired meiotic progression. Does this deficiency could activate of DNA damage checkpoint, which could result in meiotic arrest?”. Staining of CHK1/2 and P53 should be added to evaluate the activation of DNA damage checkpoint.

In the revised version, we analyzed DNA damage checkpoint activation by performing p53 immunostaining in testes from 9-11-month-old *Wt* and *Sirt7*$^{-/-}$ mice (now shown in Fig. 6i and Extended Data Fig. 6c). As shown below, and to ensure that the staining was working properly, we first stained testes from *Trip13* mutant mice (*Trip13*$^{mod/mod}$) as a positive control. As expected, these samples showed p53 accumulation in a subset of spermatocytes, consistent with previous reports (Marcet-Ortega et al., 2017).

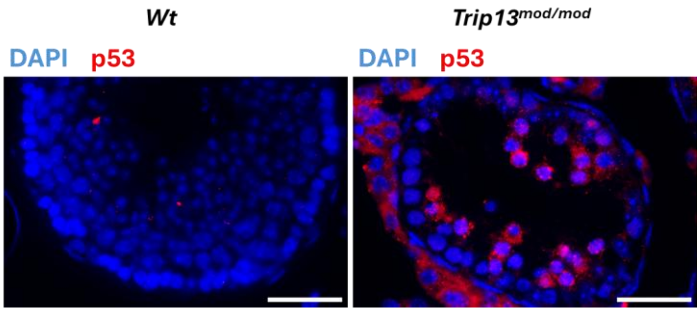

Validation of p53 staining in testicular sections. Representative images of p53 staining (Cell signaling, cat: 2524) in *Wt* and *Trip13*$^{mod/mod}$ testis sections. Scale bar = 20 µm.

In contrast, p53 staining in both *Wt* and *Sirt7*$^{-/-}$ testes was barely detectable, with approximately one positive cell per ~20 seminiferous tubules analyzed (Fig. 6i and Extended Data Fig. 6d). Although our prophase I progression analysis shows a significant increase in zygotene and early pachytene spermatocytes in 9-11-month-old *Sirt7*$^{-/-}$ testes (Fig. 6b), the low p53 signal suggests that the DNA damage checkpoint is not activated in the absence of SIRT7. Consistent with this, the proportion of spermatocytes reaching diplotene is similar between genotypes (Fig. 6b). Collectively, these findings support the interpretation that loss of SIRT7 leads to a developmental delay rather than to a developmental arrest during meiotic prophase I. These conclusions are included in the Results section, lines 311-318. In addition, we have revised the abstract to replace the term "arrest" with "delay" (line 38).

REFERENCES

Allen, C. M., Lopes, F., Mitchell, R. T., & Spears, N. (2018). How does chemotherapy treatment damage the prepubertal testis? *Reproduction, 156*(6), R209-R233. doi:10.1530/REP-18-0221

Burgess, R. C., Burman, B., Kruhlak, M. J., & Misteli, T. (2014). Activation of DNA damage response signaling by condensed chromatin. *Cell Rep, 9*(5), 1703-1717. doi:10.1016/j.celrep.2014.10.060

Endo, T., Kobayashi, K., Matsumura, T., Emori, C., Ozawa, M., Kawamoto, S., . . . Ikawa, M. (2024). Multiple ageing effects on testicular/epididymal germ cells lead to decreased male fertility in mice. *Commun Biol, 7*(1), 16. doi:10.1038/s42003-023-05685-2

Fernandez-Capetillo, O., Mahadevaiah, S. K., Celeste, A., Romanienko, P. J., Camerini-Otero, R. D., Bonner, W. M., . . . Nussenzweig, A. (2003). H2AX is required for chromatin remodeling and inactivation of sex chromosomes in male mouse meiosis. *Dev Cell, 4*(4), 497-508. doi:10.1016/s1534-5807(03)00093-5

Inselman, A., Eaker, S., & Handel, M. A. (2003). Temporal expression of cell cycle-related proteins during spermatogenesis: establishing a timeline for onset of the meiotic divisions. *Cytogenet Genome Res, 103*(3-4), 277-284. doi:10.1159/000076813

Lam, K. G., Brick, K., Cheng, G., Pratto, F., & Camerini-Otero, R. D. (2019). Cell-type-specific genomics reveals histone modification dynamics in mammalian meiosis. *Nat Commun, 10*(1), 3821. doi:10.1038/s41467-019-11820-7

Lammers, J. H., Offenberg, H. H., van Aalderen, M., Vink, A. C., Dietrich, A. J., & Heyting, C. (1994). The gene encoding a major component of the lateral elements of synaptonemal

complexes of the rat is related to X-linked lymphocyte-regulated genes. *Mol Cell Biol, 14*(2), 1137-1146. doi:10.1128/mcb.14.2.1137-1146.1994

Lopes, F., Tholeti, P., Adiga, S. K., Anderson, R. A., Mitchell, R. T., & Spears, N. (2021). Chemotherapy induced damage to spermatogonial stem cells in prepubertal mouse in vitro impairs long-term spermatogenesis. *Toxicol Rep, 8*, 114-123. doi:10.1016/j.toxrep.2020.12.023

Marcet-Ortega, M., Pacheco, S., Martinez-Marchal, A., Castillo, H., Flores, E., Jasin, M., . . . Roig, I. (2017). p53 and TAp63 participate in the recombination-dependent pachytene arrest in mouse spermatocytes. *PLoS Genet, 13*(6), e1006845. doi:10.1371/journal.pgen.1006845

Page, J., de la Fuente, R., Manterola, M., Parra, M. T., Viera, A., Berrios, S., . . . Rufas, J. S. (2012). Inactivation or non-reactivation: what accounts better for the silence of sex chromosomes during mammalian male meiosis? *Chromosoma, 121*(3), 307-326. doi:10.1007/s00412-012-0364-y

Pai, C. C., Deegan, R. S., Subramanian, L., Gal, C., Sarkar, S., Blaikley, E. J., . . . Humphrey, T. C. (2014). A histone H3K36 chromatin switch coordinates DNA double-strand break repair pathway choice. *Nat Commun, 5*, 4091. doi:10.1038/ncomms5091

Smart, E., Lopes, F., Rice, S., Nagy, B., Anderson, R. A., Mitchell, R. T., & Spears, N. (2018). Chemotherapy drugs cyclophosphamide, cisplatin and doxorubicin induce germ cell loss in an in vitro model of the prepubertal testis. *Sci Rep, 8*(1), 1773. doi:10.1038/s41598-018-19761-9

Vazquez, B. N., Thackray, J. K., Simonet, N. G., Kane-Goldsmith, N., Martinez-Redondo, P., Nguyen, T., . . . Serrano, L. (2016). SIRT7 promotes genome integrity and modulates non-homologous end joining DNA repair. *EMBO J, 35*(14), 1488-1503. doi:10.15252/embj.201593499
